# Single-cell analysis revealed that IL4I1 promoted ovarian cancer progression

**DOI:** 10.1186/s12967-021-03123-7

**Published:** 2021-10-30

**Authors:** Hongyu Zhao, Yu Teng, Wende Hao, Jie Li, Zhefeng Li, Qi Chen, Chenghong Yin, Wentao Yue

**Affiliations:** Central Laboratory, Beijing Obstetrics and Gynecology Hospital, Capital Medical University, Beijing, 100026 China

**Keywords:** Ovarian cancer, Single-cell RNA-sequencing, Heterogeneity, M2-like TAMs, Malignant epithelial cluster, Prognosis, IL4I1

## Abstract

**Background:**

Ovarian cancer was one of the leading causes of female deaths. Patients with OC were essentially incurable and portends a poor prognosis, presumably because of profound genetic heterogeneity limiting reproducible prognostic classifications.

**Methods:**

We comprehensively analyzed an ovarian cancer single-cell RNA sequencing dataset, GSE118828, and identified nine major cell types. Relationship between the clusters was explored with CellPhoneDB. A malignant epithelial cluster was confirmed using pseudotime analysis, CNV and GSVA. Furthermore, we constructed the prediction model (i.e., RiskScore) consisted of 10 prognosis-specific genes from 2397 malignant epithelial genes using the LASSO Cox regression algorithm based on public datasets. Then, the prognostic value of Riskscore was assessed with Kaplan–Meier survival analysis and time-dependent ROC curves. At last, a series of in-vitro assays were conducted to explore the roles of IL4I1, an important gene in Riskscore, in OC progression.

**Results:**

We found that macrophages possessed the most interaction pairs with other clusters, and M2-like TAMs were the dominant type of macrophages. C0 was identified as the malignant epithelial cluster. Patients with a lower RiskScore had a greater OS (log-rank P < 0.01). In training set, the AUC of RiskScore was 0.666, 0.743 and 0.809 in 1-year, 3-year and 5-year survival, respectively. This was also validated in another two cohorts. Moreover, downregulation of IL4I1 inhibited OC cells proliferation, migration and invasion.

**Conclusions:**

Our work provide novel insights into our understanding of the heterogeneity among OCs, and would help elucidate the biology of OC and provide clinical guidance in prognosis for OC patients.

**Supplementary Information:**

The online version contains supplementary material available at 10.1186/s12967-021-03123-7.

## Introduction

Ovarian cancer (OC) was one of the most fatal and aggressive tumors of the female reproductive system and had emerged at an increased incidence in recent years. Generally acknowledged treatment for OC is surgery followed by platinum and taxane-based combination chemotherapy. However, nearly 25 percent of OC patients were found to relapse within six months after combination therapy [1]. Most patients finally died from metastasis, due to a lack of other treatments aimed at improving the prognosis of OC patients. Thus, it is necessary to recognize OC-associated risks and make accurate prognostic of OC.

Effort made in understanding prognosis [2, 3] and response to platinum-based chemotherapy [4–6] in OC was highly focused on profiling gene expression and genetic aberrations. A prior report pointed out that OC patients studied by TCGA, revealed that critical gene mutations drive the pathogenesis of OC, which include the TP53 driver mutation (95%), and other major target genes including CCNE1, MYC, TERT, and NF1[7]. Known risk factors that can accelerate ovarian carcinoma progression include BRCA1/BRCA2 mutations, family history, pregnancy, and other factors [8, 9]. Although carcinogenic- and metastatic-specific mutations were confirmed to accelerate carcinogenesis, dysregulated signal transduction or genetic variation of tumor cells were also critical for cancer progression.

Exploring the mechanisms of OC progression using bulk transcriptomics are confounded by a variety of factors, including copy number variation and infiltration by non-cancerous cells. Understanding the relationship of cancer cells and the tumor microenvironment (TME) is dependent on the accurate identification and characterization of individual cell states. In addition, intra-tumorigenic heterogeneity represents a key mechanism for both OS and progression of cancer [10, 11]. Moreover, the extensive intra-tumor heterogeneity that exists between OC cells, makes accurate identification of genetic diversity based on bulk mRNA sequencing highly controversial. Actionable diagnostic markers and identified therapeutic targets were based on bulk profiling technologies, with a complete disregard for intra-tumoral heterogeneity, which was not suitable for all patients. Recent advances in single-cell sequencing provide powerful tools to explore genetic and functional heterogeneity, which should assist in resolving the problem. In addition, scRNA-seq studies have revealed new insights into intra-tumor heterogeneity and have identified distinct sub-populations, which have provided novel mechanisms in our understanding of both carcinogenesis and in revealing strategies for treatment [12–15]. However, few studies have explored OC at the single cell level.

One recent scRNA-seq study of OC performed by Shih et al., [16] investigated intra- and inter-tumorigenic heterogeneity at the cellular resolution with a large number of samples. That study not only identified several cell clusters, but also found that specific cell types were correlated with a well-known cancer subtype. Their single-cell assessment of patient samples enhanced our cognition of OC, and provided critical information that was helpful in advancing our understanding of OC progression. Regretfully, the clinical application of markers across OC progression at the single cell level was not explored, and these markers might display greater precision in the context of personalized anti-cancer therapy with consideration for intra-tumoral heterogeneity.

**Fig. 1:**
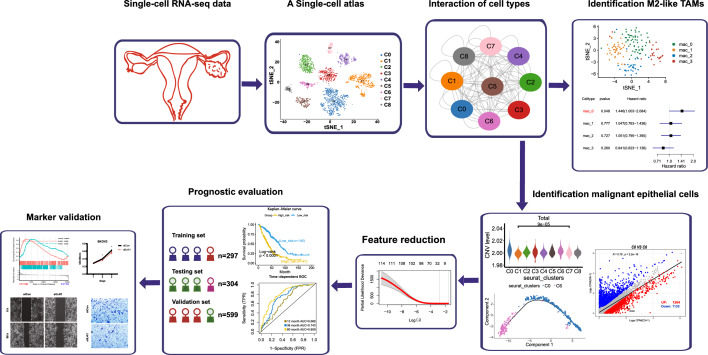
Design of experiment and work diagram of this study

To overcome this limitation, in present study we performed further bioinformatics analysis using the data from the Shih et al., [16] study, with the aim of identifying several diverse clusters. The design of this study and workflow was summarized in Fig. 1. Significantly, we identified the dominant M2-like TAMs in OC and explored the critical pathways across tumor progression in OC. In addition, we developed a RiskScore that was associated with robust prognosis in OCs based on markers of OC progression. Interleukin 4 Induced 1 (IL4I1) was an important gene in Riskscore. Notably, a variety of bioinformatic methods and experimental assays were conducted, revealing that IL4I1 accelerated cell proliferation, migration and invasion. Our work would help elucidate the biology of OC based on single-cell RNA-sequencing, which might provide clinical guidance in prognosis for OC patients.

## Methods

### Ovarian cancer and other cancer datasets

Single-cell RNA-seq for ovarian cancers (GSE118828) was downloaded from GEO database (https://www.ncbi.nlm.nih.gov/); bulk RNA-seq data and corresponding clinic-pathological data of multiple cancer patients in TCGA were obtained from UCSC Xena (https://xenabrowser.net/datapages/). Data retrieved from multiple GEO databases was used for integrated analysis using the Combat with the sva package [17]. All public data used in this study is described in Additional file 1: Table S1.

### Single-cell RNA-seq data preprocessing

The barcode matrix was processed with Seurat v3 [18], a toolkit for single-cell RNA-seq data analysis. All functions were run with default parameters, unless otherwise specified. Low quality cells (< 300 genes/cell, and < 3 cells/gene) were excluded. The UMI count data was normalized by log-transformation. The top 2000 highly variable genes (HGVs) were selected to aggregate samples into a merged dataset. Next, the merged cells-by-genes matrix was scaled by dividing the centered expression by the standard deviation. Batch effects among patients were eliminated using the “RunHarmony” function with the harmony package [19]. The top 20 principal components, along with HGVs were used in this process.

Subsequently, the main cell clusters were identified using the “FindClusters” function of Seurat and visualized using the t-distributed stochastic neighbor embedding (tSNE) function. DEGs were appraised using the “FindMarkers” or “FindAllMarkers” function with the default parameter. For sub-clustering analysis, we applied the same procedure of finding variable genes, dimensionality reduction, and clustering.

Cluster annotation was based on classical markers. We characterized the identities of cell types based on known markers: EPCAM and KRT18 [20] (epithelium), ACTA2 [20] (mesenchyme), CD163 and CD68 [21] (macrophage), CCL5 [22] and IL7R [23] (T cell), VWF and CDH5 [21] (endothelium), CD79A and MS4A1 [23] (B cell).

### The chromosomal copy number variation (CNV) estimation

Initial CNVs for each region were estimated by the infer-CNV package [24]. The CNV of total cell types were calculated by expression levels from single-cell sequencing data for each cell with a cut-off 0.1. The CNV score of each cell was calculated as the mean of the CNV region.

### Pseudotime analysis

Single cell trajectories were performed to explore cell-state transitions using the Monocle2 package [25]. Differentially expressed genes over the Pseudo-time among cluster cell transitions were calculated by the “differentialGeneTest” function (q value < 0.1). The “DDRTree” was applied to reduce dimensions and visualization functions, and the “plot_cell_trajectory” was used to plot the minimum spanning tree on cells.

### Recognition of malignant transcription factors (TFs)

In order to identify malignant TFs, we extracted a list of all identified TFs from Animal TFDB 2.0 (http://bioinfo.life.hust.edu.cn/AnimalTFDB2/about.shtml). We compared the TFs list with 2397 DEGs from 2 different epithelial cells (C0 and C6), and identified the malignant TFs.

### Gene set functional analysis

Gene set functional analysis was done by the clusterProfiler package [26], gsva package [27], and DAVID (https://david.ncifcrf.gov/home.jsp). The h.all.v7.0.symbols.gmt and c2.cp.reactome.v7.0.symbols.gmt were down-loaded from the Molecular Signatures Database (http://www.broad.mit.edu/gsea/msigdb/). To investigate IL4I1 mediated biological parameters in OC, gene set enrichment analysis (GSEA) was conducted by clusterProfiler package. False discovery rate (FDR) < 0.05 and P < 0.05 were utilized as the enriched terms. In addition, 308 OC patients in the TCGA dataset were divided into high-expression group and low-expression group according to the median value of IL4I1.

### Construction a single-cell transcriptome network

To explore the relationship between the clusters, a Python-based computational analysis tool for single-cell RNA-seq data analysis—CellPhoneDB [28] was used to construct the single-cell transcriptome network. Namely, ligand-receptor pairs for each cluster with other clusters were generated.

### Construction a RiskScore with malignant marker genes

To build a novel RiskScore associated with survival in OC, firstly, malignant marker genes associated with survival were selected at P < 0.05 with the univariate Cox proportional hazards regression model, 117 of 2397 genes were identified with statistical significance in the GEO OC meta-dataset1 (an integrated OC cohort: GSE14764, GSE23554, and GSE26712 with GPL96). Subsequently, the 117 genes were narrowed down using the penalized logistical least absolute shrinkage and selector operation (LASSO) algorithm. The GEO OC meta-dataset1 as the training cohort and TCGA OC dataset used as the testing cohort. Using OS as the predictor variable, this procedure was repeated 10,000 times to construct the RiskScore, which generated with a linear combination of expression values and LASSO coefficients of signature genes using the following formula:

$$ {\text{Y}}\, = \,[{\text{ZNF44}}0\, \times \,\left( { - 0.{237}} \right)\, + \,{\text{USP53}}\, \times \,\left( { - 0.{455}} \right)\, + \,{\text{TSPAN12}}\, \times \,0.{469}\, + \,{\text{RARS}}\, \times \,\left( { - 0.{153}} \right)\, + \,{\text{NFX1}}\, \times \,\left( { - 0.{246}} \right)\, + \,{\text{LRRC6}}\, \times \,\left( { - 0.{369}} \right)\, + \,{\text{IL4I1}}\, \times \,\left( { - 0.{258}} \right)\, + \,{\text{GPC1}}\, \times \,\left( {0.{269}} \right)\, + \,{\text{CD59}}\, \times \,\left( { - 0.{386}} \right)\, + \,{\text{ARID5B}}\, \times \,0.{334}]. $$

Kaplan Meier survival analysis and time-dependent ROC curves were used to assess the performance of gene signatures. Patients were divided into a high- and low-RiskScore group based on the median value of RiskScore. The Kaplan–Meier survival curves of the RiskScore were generated using the log-rank test. The time-dependent receiver operating characteristic (ROC) curve was generated with the timeROC package. The prognostic value of the RiskScore was externally validated with GEO OC meta-dataset2 (an integrated OC cohort: GSE18520, GSE26193, GSE30161, GSE63885, GSE54388, and GSE9891 with GPL570), and other TCGA cancers.

### Cell culture and siRNA transfection

All OC cell lines (SKOV3, A2780 and CAOV8) were obtained from ATCC. SKOV3 and A2780 were cultured in Roswell Park Memorial Institute (RPMI)-1640 medium supplemented with 10% fetal bovine serum (FBS) and 100 U/ml penicillin/streptomycin. CAOV8 was cultured in high-glucose Dulbecco’s modifed Eagle’s medium (DMEM) with 10% FBS and 100 U/ml penicillin/streptomycin. All cell lines were incubated at 37 ℃ with 5% CO_2_.

All cell lines are transfected with Lipofectamine™ RNAmax according to the manufacturers’ instructions. IL4I1-target specific small interfering RNA (siRNA) is synthesized by JTSBIO Co., Ltd. (Wuhan, China). The sequences of IFI6-target-specifc-siRNA (siIL4I1) were as follows: siRNA1, 5ʹ-GCAUGCAGGAUCCUGACUATTUAGUCAGGAUCCUGCAUGCTT-3ʹ; siRNA2, 5ʹ-GCGAUGAAGAAGUUUGAAATTUUUCAAACUUCUUCAUCGCTT-3ʹ, siRNA3, 5ʹ-GCUUCUUCUAUCUCAGCUUTTAAGCUGAGAUAGAAGAAGCTT-3ʹ; and the sequence of control is 5ʹ-UUCUCCGAACGUGUCACGUTTACGUGACACGUUCGGAGAATT-3ʹ. The transfected cells were cultured in a fresh medium for 24 h for the subsequent assays.

### Western blot analysis

Total protein was extracted using RIPA buffer (Thermo Fisher Scientific, Waltham, MA, USA) and detected utilizing BCA assay (Thermo Fisher Scientific, Waltham, MA, USA). 30 μg of protein per sample was separated by SDS-PAGE, then transferred onto PVDF membrane (Gene Molecular Biotech, Inc., Shanghai, China). After obturated with 5% milk for 2 h at room temperature, the membrane was incubated overnight at 4 °C with primary antibodies as follow: GAPDH (1:1000, CST), MMP2 (1:1000, CST), MMP9 (1:1000, CST). Afterwards, the membrane was incubated with HRP-conjugated rabbit IgG secondary antibodies (1:7500, CST) for 1 h at room temperature, the expression level was measured with an ECL kit (Roche Diagnostics, Basel, Switzerland) by Western blot imaging system.

### Cell proliferation assays

1 × 10^3^ cells were incubated in 96-well plates. Then, 10 uL Cell Counting Kit-8 (CCK-8; Dojindo, Rockville, MD, USA) solution was added to each well and incubated for 2 h for evaluating cell proliferation. The absorbance of each well was measured at OD450 with a Tecan Infinite M1000 PRO (Tecan, Switzerland) from days 1 to 4.

### Cell wound healing assay

The transfected SKOV3 were cultured in 6-well plates with 1 × 10^6^ cells/well for 24 h. Then, the wounds were produced using a 100 μL pipette tip. The images were photographed with a microscope after 0 and 96 h. The scratch area was measured to evaluate cell migration ability by image J software.

### Transwell assays

The transwell migration and invasion assays were conducted with Corning Transwell Inserts (8.0 μm). For the transwell migration assay, 1.5 × 10^4^ transfected cells suspended in 50 μL serum-free medium were placed in the upper chamber and 600 μL medium (10% FBS) was filled in the lower compartment. The cells were incubated at 37 ℃ for 24 h. The successfully translocated cells were fixed with 4% paraformaldehyde (PFA) and stained with 0.1% crystal violet, and counted in four randomly chosen fields (200×) under a microscope.

For the transwell invasion assay, 1.2 × 10^5^ cells were seeded on transwell coated with 50 μL Matrigel (dilution of 1:4 with 0.2% BSA). It is worth noting that Matrigel was used to coat membranes for 12 h at 37 °C prior to invasion assays. The culture condition was the same as the transwell migration assay. The cells on the lower surface were fixed, stained, and photographed microscopically after 48 h.

All the statistical analyses were performed using R 3.6.1 and Graphpad prism 8.0 software. The assay was repeated at least three times and the data were presented as mean ± standard deviation (SD). Two-tailed Student’s t-test was used to assess the differences between two groups. *P* < 0.05 was considered statistically significant.

## Results

### A single-cell atlas of ovarian cancers and control samples

**Fig. 2:**
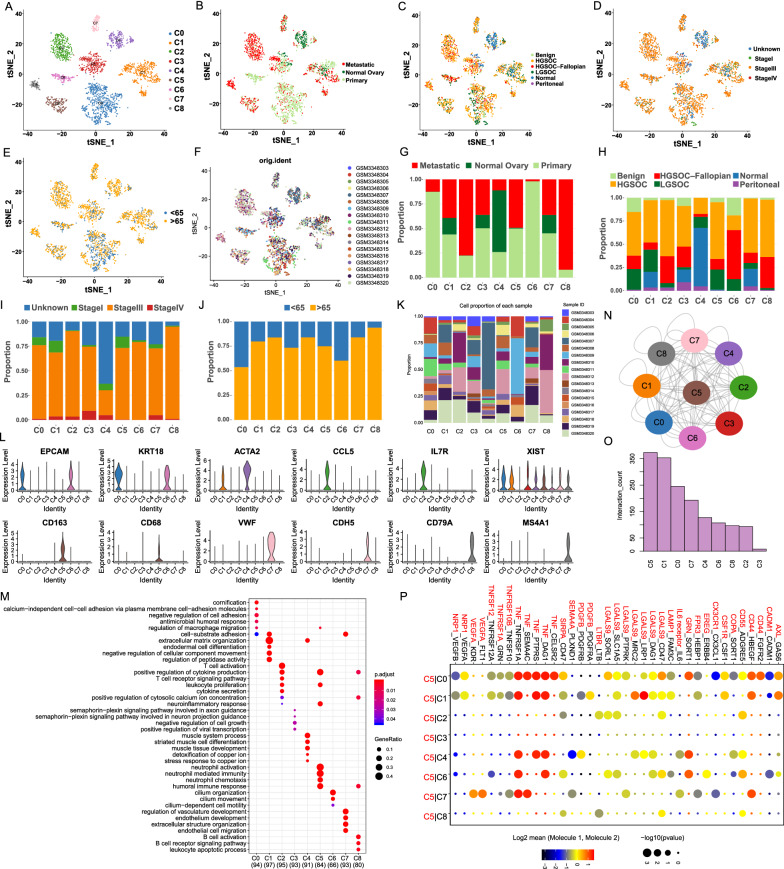
Overview of single-cell transcriptomes derived from OCs. **A**–**F**) TSNE plot of all single cells, with each color coded for the following: **A** 9 major cell types; **B** sample origin (normal, primary or metastatic); **C** type of neoplasm; **D** tumor stage; **E** age and **F** samples. The proportion of diverse cell types across different **G** sample origins, **H** type of neoplasm, **I** tumor stage, **J** age and **K** samples. **L** Violin plots exhibiting the expression of representative markers across diverse cell types. The y axis was the normalized read count. **M** Functional analysis of each cluster was illustrated with GO analysis. **N** Interaction network constructed by CellPhoneDB. **O** The barplot illustrating the ranks of ligand-receptor pairs with cell types interactions. **P** Heatmap of ligand-receptor interactions between diverse cell types and C5. Point size indicates *p*-value (CellPhoneDB). Colour indicates the mean expression level of ligand and receptor (Mol1/2)

Eighteen samples obtained from GSE118828 were studied, namely a total of 17 neoplasms including 1 benign cancer, 1 peritoneal cancer, 3 LGSOC, 12 HGSOC, and 1 control ovarian. After removing low quality cells, a total of 3066 cells were finally acquired. In Shih’s [16] paper, they only studied 14 samples and captured 2911 cells. A reason for disparate cell count between our study and Shih’s might be the inequable samples we analyzed. These cells were classified into 9 main cell lineages namely C0-C8 (Fig. 2A). The clinical information of each cell population was illustrated in Fig. 2B–F. The corresponding proportions for each cluster with different clinical characteristics were discrepant (Fig. 2G–K). Based on the expression of well-known markers (Fig. 2L), we found that the atlas mainly comprised two different types of epithelial cell (i.e., C0 and C6), two mesenchyme clusters (i.e., C1 and C4), a T cell cluster (C2), a macrophage cluster (C5), an endothelium cluster (C7), and a B cell cluster (C8). C3 was an XIST highly expressed cluster. The gene ontology (GO) analysis for each cluster also confirmed the cluster annotation (Fig. 2M).

To explore the relationship between these clusters in OC, we used CellPhoneDB to calculate potential ligand-receptor pairs of each cluster and construct the single-cell transcriptome network. Network was visible using Cytoscape (Fig. 2N). Notably, macrophages possessed the most interaction pairs with other clusters, revealing the dominant role of macrophages in OC (Fig. 2O). As shown in Fig. 2O, TNF, TNFRSF10B, LGALS9, CX3CR1, VEGFA, and LAMP1 secreted by C5 interact with receptors expressed on epithelial cells, mesenchymal cells, endothelial cells, and other immune cells. These ligand-receptor pairs may be related to immune, angiogenesis, and CAF proliferation.

### Macrophages exhibit M2 polarization in OC

**Fig. 3:**
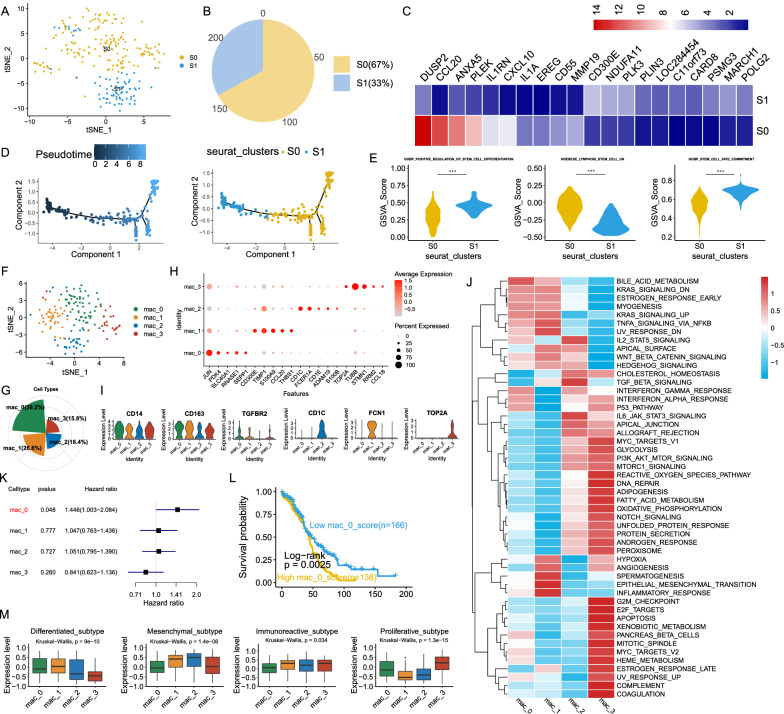
Macrophages exhibited M2 Polarization in the in OCs. **A **TSNE representation of two subgroups generated from macrophages. **B** Proprotions of S0 and S1. **C** Heatmap illustrated top 10 genes of each subcluster. **D** Pseudotime graph illustrated the differentiated trajectory between S1 and S0. **E** Violin plots illustrating that higher stem score enriched in S1. **F** TSNE representation of four subgroups from S0. **G** Proprotions of four subgroups in S0. **H** Dotplot shown top 5 genes of each subgroup. **I** Violin plots shown the representative markers of four subgroups. **J** GSVA analysis with hallmark terms for mac_0 to mac_3. **K** Forest plot illustrating the survival associated with gsvascore of four subgroups in OC, mac_0 was associated with survival. **L** Kaplan–Meier plot illustrating that OC patients with higher mac_0 gsvascore demonstrated worse survival. **M** Gsvascore of four subgroups based on top 5 markers in different clinical phenotypes

For discussing the heterogeneity among macrophages, 236 macrophages were reclustered into 2 subclusters basis on tSNE analysis, including 158 cells in S0 and 78 cells in S1 (Fig. 3A, B). Top 10 genes of each subcluster were shown Fig. 3C. Pseudotime analysis suggested that a differential process existed from S1 to S0 (Fig. 3D). In addition, we explored the stem score with GSVA based on multiple terms, it showed that higher stem score enriched in S1 (Fig. 3E). Thus, we inferred that S0 might originate from S1. Based on the trajectory graph, it seemed that S0 had different branches of differentiation, meaning that cells in S0 had different differential fate, and the heterogeneity existed in S0. Thus, we reclustered S0 cells to 4 cluster (Fig. 3F), mac_0 was consisted of most S0 cells (Fig. 3G). Subsequently, we explored their characteristics, top 5 markers of each cell types were shown in Fig. 3H. Obviously, the monolike marker, FCN1, was highly expressed in mac_1; the DC markers, CD1C/E, were highly expressed in mac_2; markers associated with proliferation related genes and functions were highly enriched in mac_3, such as TOP2A, G2M_CHECKPOINT, and etc. (Fig. 3H–J). To deeply expounded their clinical features in OC, we calculated the GSVA score using top 5 genes of each celltype. As a results, OC patients with higher mac_0 gsvascore demonstrated worse survival (Fig. 3K, L). According to violinplot, the M2-like TAMs marker, TGFBR2, was highly expressed in mac_0. Thus, we inferred that mac_0 represented an M2-like TAMs cluster, which was the dominant type of macrophages in OC. Furthermore, we explored the gsvascore of four celltypes in different clinical phenotypes (Fig. 3M). As a results, mac_3 gsvascore was highest in proliferation_subtype, which was accorded with our previous finds that mac_3 was associated with proliferation.

### Malignant epithelial cells were distinguished in OCs

**Fig. 4:**
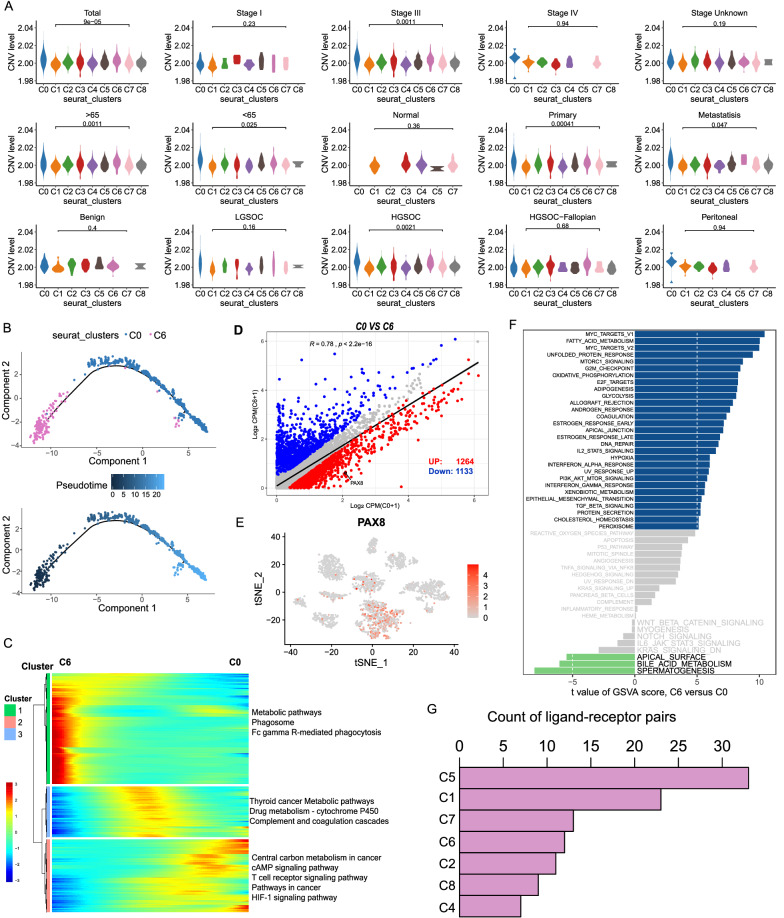
C0 was a malignant epithelial cluster in OCs. **A** Violin plots showing CNV scores across different cell types in OCs. **B** Pseudotime of epithelial cells with abnormal gene expression profiles and malignant epithelial cells that were inferred by Monocle2. Each point corresponds to a single cell. **C** Heat-map showing DEGs (rows) along the pseudo-time (columns), which was clustered into three profiles. Color key differentially coding from blue to red that indicated the relative expression levels from low to high. **D** Scatter plots showing relative gene expression levels of C0 (x axis) and C6 (y axis) in OCs. **E** TSNE plot illustrated that PAX8 was only existed in C0. **F** GSVA analysis for C0 and C6. **G** The ranks of ligand-receptor pairs interactions between C0 and other cell types

As we were aware, ovarian cancer mainly originated from the epithelium. In the present study, the presence of two different types of epithelial cells (C0 and C6) encouraged us to investigate their malignant status. The chromosomal copy number variation (CNV) score of each cell help to identify the malignant clusters. First, we calculated large-scale CNV in each cell type based on averaged expression patterns across intervals of the genome. For all cell groups and different subtypes, we found that C0 exhibited remarkably higher CNV levels than C6 and other types of cells (Fig. 4A). The CNV score of each subtype and each sample was shown in Additional file 1: Fig. S1.

Then, we analyzed the gene expression profiles along the trajectory of C0 and C6 with pseudotime analysis and demonstrated a differentiated process from C6 to C0 (Fig. 4B). Heatmap analysis showed dynamically changed genes from C6 to C0 and identified 3 clusters (Fig. 4C). Functional enrichment analysis illustrated that multiple oncogenesis pathways were activated during OC progression, such as the cAMP signaling pathway, the T cell receptor signaling pathway, pathways in cancer development and progression, and the HIF-1 signaling pathway (Fig. 4C). We also explored the expression of multiple TFs that associated with the tumorigenesis of OC, such as the oncogene MYC (Additional file 1: Fig. S2).

We compared discrepant genes between C0 and C6, identifying 1264 upregulated and 1133 down-regulated genes in C0 (Fig. 4D, Additional file 1: Table S2). The peculiar marker used in the clinical identification of the malignant OCs, PAX8, was highly expressed in C0 (Fig. 4D, E). In addition, GSVA demonstrated that C0 was significantly related to carcinogenic terms, such as MYC_TARGETS, G2M_CHECKPOINT, EPITHELIAL_MESENCHYMAL_TRANSITION, etc. (Fig. 4F). Thus, C0 was the malignant epithelial cluster. In addition, we explored the ligand-receptor pairs interactions between C0 and other cell types. As a results, C0 had the most relationship with C5 (Fig. 4G), certifying the close contact between tumor cells and macrophages in OC carcinogenesis.

### Distinct subgroups in the epithelial cluster

**Fig. 5:**
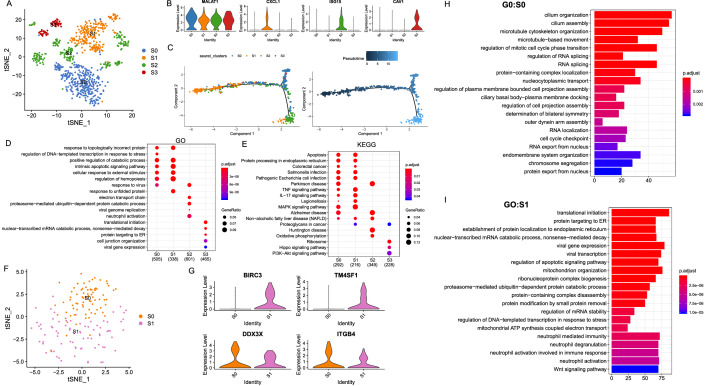
Diverse subgroups in 2 epithelial clusters. **A** TSNE representation of 4 subgroups generated from malignant epithelial cells, C0. **B** Expression levels of representative genes in each subgroup were plotted in the violin plots. **C** Pseudo-time of 4 subtypes in C0 that were inferred by Monocle2. Functional analysis of 4 subtypes in C0 illustrated with GO (**D**) and KEGG (**E**) analysis. **F** TSNE illustrated 2 subgroups that were generated from C6. **G** Violin plots showing the expression levels of representative genes in each subgroup. GO terms of S0 (**H**) and S1 (**I**)

To identify the heterogeneity of epithelial cells in OC, we reclustered the two former epithelial clusters. Four distinct subgroups in the malignant epithelial cluster were identified on basis of tSNE analysis (Fig. 5A). We noticed that specific markers of S0-S2 were related to immunity; CAV1 was related to carcinogenesis and was specifically shown in S3 (Fig. 5B). Pseudotime graphs demonstrated that S1 represented the original cells that could differentiate into S0 and S2 (Fig. 5C). Functional terms confirmed that S0-S2 were related to immunity, and S3 was related to carcinogenesis, including the Hippo signaling pathway and the PI3K-AKT signaling pathway (Fig. 5D, E).

Another epithelial cluster, C6, was reclustered to two subtypes (Fig. 5F). S0 was found to highly express ITGB4 and DDX3X, and was associated with the functions of RNA splicing and cell cycle checkpoint (Fig. 5G, H); and S1 specifically expressed BIRC3 and TM4SF1, which was related with protein targeting functions to the ER and neutrophil-mediated immunity (Fig. 5G, I). The above observations indicated that epithelial cells were closely associated with host immunity.

### Construction a robust prognostic model based on malignant epithelial markers in OC

To explore the clinical application of gene expression patterns in malignant epithelial cells, we used univariate Cox proportional hazards regression and LASSO algorithms to narrow down 2397 malignant genes. The GEO OC meta-dataset1 (an integrated OC cohort: GSE14764, GSE23554, and GSE26712 with GPL96) was used as the training set and the TCGA OC was used as the testing set. Ultimately, we selected 10 prognosis-specific genes to construct the RiskScore. The formula of the RiskScore was Y = [ZNF440 × (− 0.237) + USP53 × (− 0.455) + TSPAN12 × 0.469 + RARS × (− 0.153)  + NFX1 × (− 0.246) + LRRC6 × (− 0.369) + IL4I1 × (− 0.258) + GPC1 × (0.269) + CD59 × (− 0.386) + ARID5B × 0.334]. In addition, the prognostic value of the RiskScore was externally validated with GEO meta-dataset2 (an integrated OC cohort: GSE18520, GSE26193, GSE30161, GSE63885, GSE54388, and GSE9891 with GPL570).

**Fig. 6:**
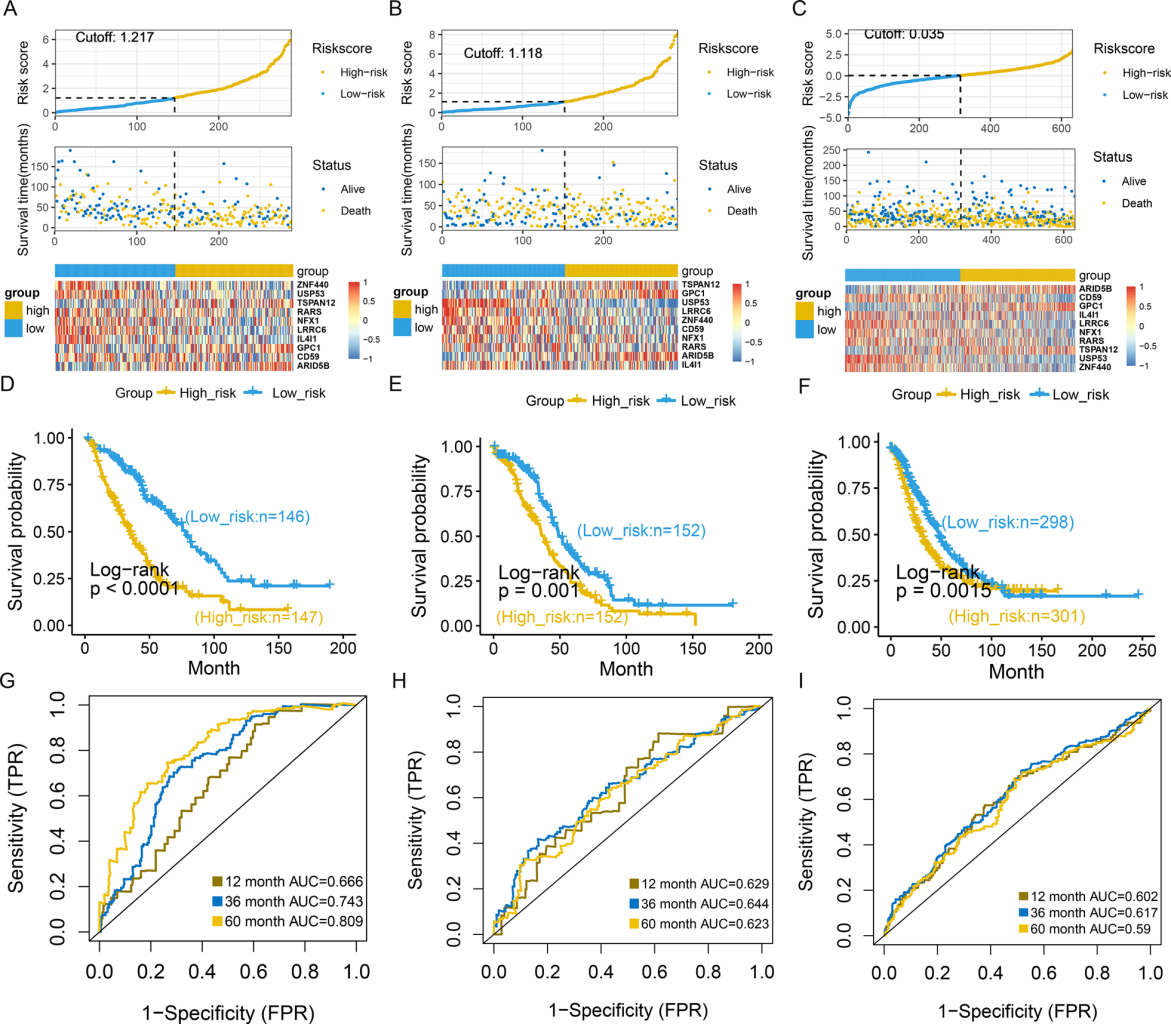
Excellent prognosis of RiskScore based on carcinogenic genes. (**A**–**C**) RiskScore distribution, survival status, and gene expression profile, (**D**–**F**) Kaplan–Meier survival curves, (**G**–**I**) time-dependent ROC curve analyses for patients in high- and low-RiskScore groups in GEO OC meta-dataset1 training set, TCGA OC testing set, and GEO OC meta-dataset2 validation set

RiskScore analysis for 10 specific biomarkers in OC patients was shown in Fig. 6A–C. Significantly, RiskScore was negative association with survival in OC patients (log-rank P < 0.01; Fig. 6D–F). RiskScore was reliable and robust to predict the survival of OCs based on time-dependent ROC curves (Fig. 6G–I). As a result, the area under curve (AUC) in GEO OC meta-dataset1 validation set was 0.666, 0.743 and 0.809 in 1-year, 3-year and 5-year survival, respectively. In TCGA testing set, the AUC was 0.629, 0.644 and 0.623 for 1-year, 3-year and 5-year survival, respectively. Meanwhile, we found that RiskScore also had a high predictive accuracy of survival in GEO OC meta-dataset2 validation set. Furthermore, we explored the prognostic capability of the RiskScore of multiple cancers in TCGA, and the forest plot demonstrated the diverse OS across multiple cancers (Additional file 1: Fig. S3). This indicated the good potential of RiskScore in survival monitoring.

### IL4I1 accelerated OC cells proliferation, invasion and migration

**Fig. 7:**
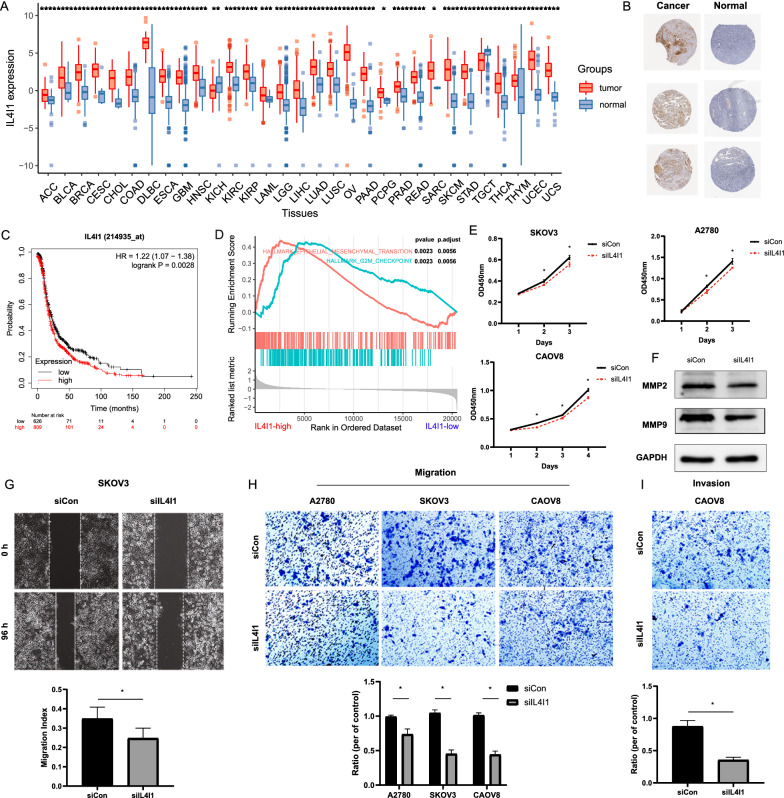
IL4I1 was a carcinogenesis gene in OC. **A** The mRNA expression level of IL4I1 was higher in OCs than paired normal samples. **B** Representative protein expression level of IL4I1 was high in OCs based on the Human Protein Atlas database. **C** Kaplan–Meier curve illustrating higher IL4I1 accompanied by poor OS. **D** Functions of IL4I1 with GSEA in OC. **E** Expression of MMP2, MMP9 were lower in siIL4I1 cells in protein level. **F **Downregulation of IL4I1 slow downed OC cell lines proliferation. **G** siIL4I1 significantly inhibited wound closure compared to the corresponding controls. Migratory (**H**) and invasive (**I**) cells were dramatically reduced in OC cells transfected with siIL4I1

IL4I1, an important gene in Riskscore model, was reported to play important roles in immunoregulation and tumor progression [29–32]. In present study, IL4I1 was higher in multiple cancers than paired normal samples in GTEx (Fig. 7A). Noteworthy was the observation that the representative protein expression level of IL4I1 was positive in OCs based on the Human Protein Atlas database (HPA) (Fig. 7B). In truth, IL4I1 upregulation was associated with poor OS in OC (Fig. 7C). GSEA shown that high-IL4I1 group were mainly associated with G2M Checkpoint and EMT (Fig. 7D). As validation, downregulation of IL4I1 notably inhibited the proliferation of SKOV3, A2780 and CAOV8 (Fig. 7E).

Given the effect of IL4I1 on OC cells metastasis, the expression of MMP2 and MMP9 were lower in siIL4I1 group (Fig. 7F). Furthermore, siIL4I1 significantly inhibited wound closure in SKOV3 compared to the control cells based on wound healing assay (Fig. 7G). In the transwell migration and invasion assay, migrated (Fig. 7H) and invasive (Fig. 7I) cells transfected with siIL4I1 were remarkably decreased, as compared to the control groups. Thus, IL4I1 was carcinogenesis in OC.

## Discussion

Ovarian cancer was characterized as having a poor rate of survival and being deficient in an effective treatment because of the inherent intra-tumoral heterogeneity. Studies have attempted to identify distinct sub-populations and to explore mechanisms in disease carcinogenesis and strategies for the treatment of multiple cancers with scRNA-seq [12–15]. However, the gene expression profiles of different gene clusters in OC are unclear. It is highly desirable to explore the heterogeneity of OC and to explore the underlying mechanisms that are crucial in improving OC prognosis.

According to Shih’s [16] study, they captured 2911 cells from 14 samples. However, we downloaded 18 samples from the GEO datasets and ultimately obtained 3066 cells which was different from them. In this study, we conducted a comprehensive single-cell expression atlas of the 3066 cells and identified nine diverse cell types of OC, including epithelial cluster, mesenchyme cluster, macrophage cluster, T cell cluster, endothelial cluster, and B cell cluster. Despite our cluster number was disparate with Shih’s 16 clusters, the cell types in our study were similar with that described by Shih et al., demonstrating the reliability of our methods. In our study, we undertook a more thorough analysis using their OC single-cell datasets. We identified M2-like TAMs were the dominant type of macrophages in OC; in addition, we explored the potential mechanism of tumorigenesis and clinical application of malignant epithelial markers in OC.

In this study, macrophages exhibited the most ligand-receptor pairs with other clusters, revealing its’ important role in OC. Macrophages were reclustered to two subtypes that revealed the heterogeneity of macrophages. Studies revealed that TAMs could promote tumorigenesis through TGFβ signaling in tumor cells [33–35]. In present study, we observed some carcinogenesis genes such as TGFBR2 were highly expressed in S0. GSVA demonstrated that more carcinogenesis hallmark terms existed in S0, such as EMT, angiogenesis, and PI3K_AKT_MTOR_SIGNALING, etc. In addition, pseudotime analysis illustrated a differentiated trajectory from S1 to S0. Thus, we inferred that S0 was M2-like TAMs, which accounted for a greater proportion of macrophages in OC. Crosstalk between high EMT, angiogenesis and TAMs might exist in OC, influencing OC progression. TAM-derived exosomes enriched miRNA, lncRNA, and specific proteins that were contribute to tumor cell dissemination in gastric cancer [36]. A high density of CD206 + TAM was significantly associated with worse survival in colon cancer [37]. CAF might have crosstalk with TME and contribute to cancer biology by inducing the EMT process [38]. Tumour angiogenesis could be indirectly regulated by promoting M2 polarization of macrophages in liver cancer [39].

Epithelial ovarian cancer (EOC) accounts for the majority OC cases and advanced EOC eventually develops into a recurrent platinum-resistant disease. In the present study, we identified two epithelial cell types with different gene expression patterns, namely C0 and C6, which demonstrated heterogeneity in epithelial cells. C0 predominantly comprised HGSOC cells and C6 mainly comprised fallopian cells. Pseudotime analysis demonstrated a differentiated trajectory from C6 to C0; furthermore, CNV-based analysis demonstrated that C0 was a relatively malignant epithelial cluster. GSVA identified some carcinogenic terms that were enriched in C0, and the classical clinical marker for identification of malignant OCs, PAX8, was only found in C0. The above observations collectively demonstrated that C0 belonged to malignant epithelial cells and displayed wide divergence in diverse epithelial cells because of their differential origins. A similar study exploring an OC differentiated trajectory with single-cell sequencing has not previously been reported. Furthermore, our study found that the malignant epithelial cells possessed the most interaction pairs with macrophages, and macrophages play important roles in tumorigenesis and had crosstalk with tumor cells. Macrophages M2 polarization was related to malignancy events in cancers [40, 41]. Meaningfully, some immune-associated subtypes were identified for two epithelial clusters, which considered the heterogeneity of epithelial cells in OC and supported the important roles of immunity in OC disease progression.

To explore the clinical application of markers in malignant epithelial cells, we construct a Riskscore consisted of ten genes, which presented encouraging prognostic value in predicting survival in OCs. During the past years, several signatures have been identified for prognostic prediction based on bulk mRNA transcription dataset [42, 43]. However, they ignored the heterogeneity of tumors. In this study, RiskScore generated with carcinogenic genes in single-cell levels which was more credible. Interleukin-4 Induced gene 1 (IL4I1), an important gene in Riskscore model, was reported to play important roles in immunoregulation and tumor progression [29–32].

IL4I1 was strongly detected in the tumor bed of most human tumor types [44] and was identified as a prognostic biomarker [45, 46]. Our results were similar with them. In the current research, prognostic IL4I1 was higher in ovarian tumors compared to the normal ovary. Dysregulated signal transduction of tumor cells was critical for cancer progression, and carcinogenic- and metastatic-specific genes that were confirmed to accelerate carcinogenesis. In our study, we linked IL4I1 to tumor intrinsic malignant properties. As a result, we disclosed that IL4I1 promotes OC cell proliferation, migration and invasion. IL4I1 was a secreted L-phenylalanine oxidase expressed by antigen-presenting cells. IL4I1 increased the threshold of T-cell activation, inhibiting T-cell proliferation or differentiation [29, 44, 47], thus influencing immune microenvironment. Imbalance of tumor cell and immune cell might promote cancer progression. By enhancing systemic Trp-catabolism, IL4I1 contributed to a systemic tumor-promoting environment that allowed tumor cells to migrate and protected them from immune destruction [29]. Thus, IL4I1 might promote tumor progression through influencing tumor cell motility and adaptive immunity, and regulating the priming of tumor-specific immune cell, such as T cells [29, 31, 32, 47], B cell [48, 49] and macrophage [48].

## Conclusion

Our findings provided a new perspective for understanding the progression of OC. We identified the dominant M2-like TAMs in OC and recognized some novel markers of tumorigenesis. In addition, we developed a RiskScore that was associated with robust prognosis in OCs based on markers of OC progression. Furthermore, we demonstrated that IL4I1 was an oncogene and promoted OC progression. This approach was potentially helpful for personalized anti-cancer strategies in the setting of OC.

## Supplementary Information

**Additional file 1.** Supplementary Figures and Tables.

## Data Availability

All the datasets used in the present research are summarized in Additional file 1: Table S1.

## Abbreviations

AUC: The area under curve
CNV: Chromosomal copy number variation
DMEM: Dulbecco’s modifed Eagle’s medium
EOC: Epithelial ovarian cancer
FDR: False discovery rate
GSEA: Gene set enrichment analysis
GSVA: Gene set variation analysis
HPA: The Human Protein Atlas database
IL4I1: Interleukin 4 Induced 1
LASSO: The penalized logistical least absolute shrinkage and selector operation
OC: Ovarian cancer
ROC: Receiver operating characteristic
RPMI: Roswell Park Memorial Institute
FBS: Fetal bovine serum
SD: Standard deviation
TAMs: Tumour-associated macrophages
TFs: Transcription factors
TME: Tumor microenvironment
tSNE: The t-distributed stochastic neighbor embedding
