# Supplementary material for: Single-cell analysis revealed that IL4I1 promoted ovarian cancer progression
Source: J Transl Med. 2021 Oct 30;19:454. doi: 10.1186/s12967-021-03123-7 (PMC8557560; doi:10.1186/s12967-021-03123-7)
Supplement: Supplementary file 1 — Additional file 1. Supplementary Figures and Tables. [file 12967_2021_3123_MOESM1_ESM.docx]

**Single-cell analysis revealed that IL4I1 promoted ovarian cancer progression**

Hongyu Zhao^1^, Yu Teng^1^, Wende Hao^1^, Jie Li^1^, Zhefeng Li^1^, Qi Chen^1^, Chenghong Yin ^1#^, Wentao Yue^1#^

1 Central Laboratory, Beijing Obstetrics and Gynecology Hospital Capital Medical University, Capital Medical University, Beijing 100026, China

*These authors contribute equal to this work

^#^ Corresponding authors

Email: yinchh@ccmu.edu.cn

E-mail: yuewt@ccmu.edu.cn (WTY)

**Fig. S1 (A)** **Violin plots showing CNV scores across different clinical characteristics in OCs. (B) Heatmap showing large-scale CNVs of each OC sample.**


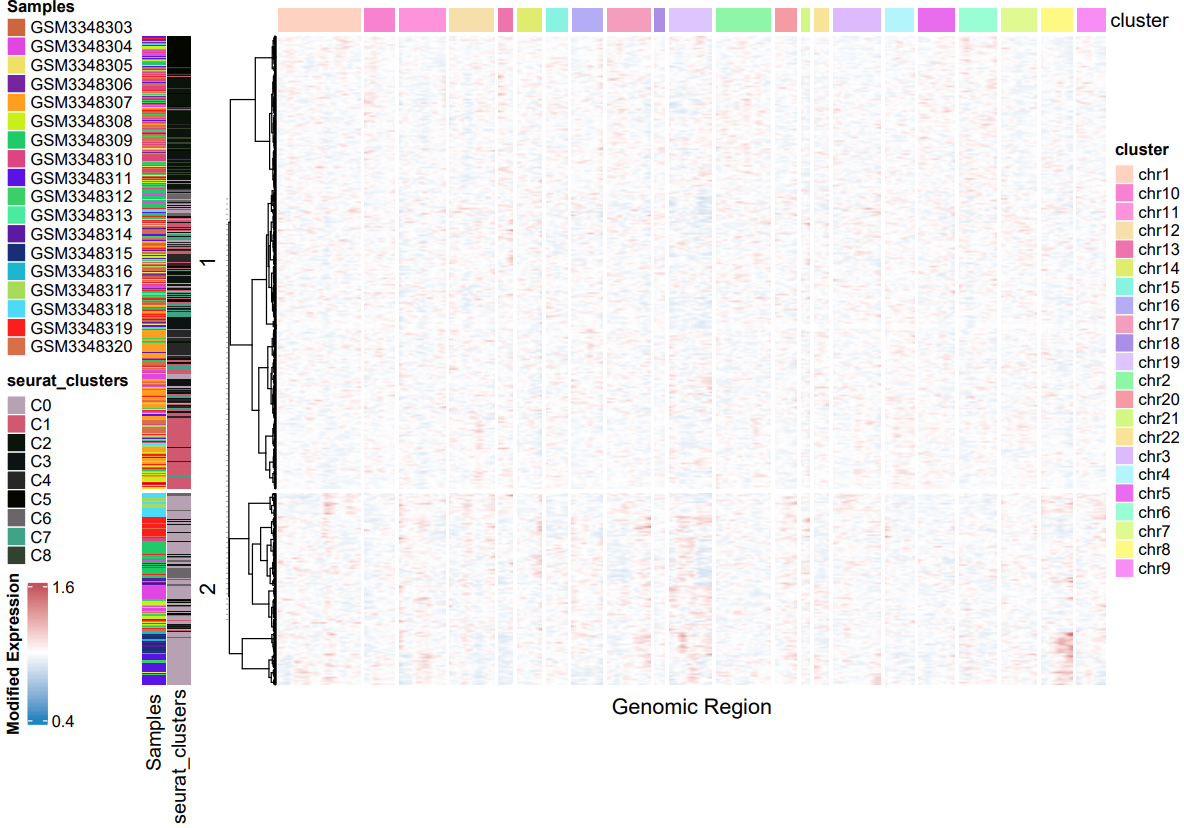


**Fig. S2 Heat-map showing key TFs (rows) along the pseudo-time (columns).**


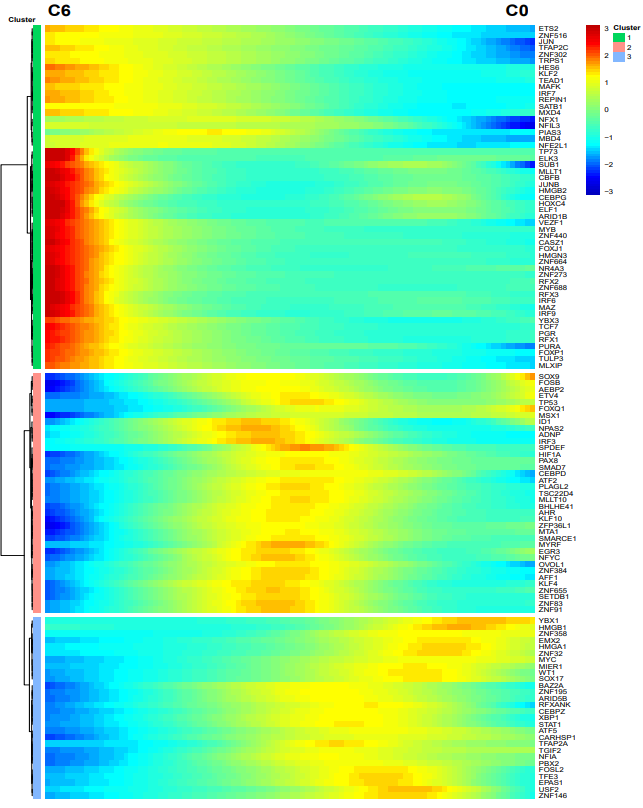


**Fig. S3** **Forest plot illustrating the survival associated with Riskscore in multiple cancers.**


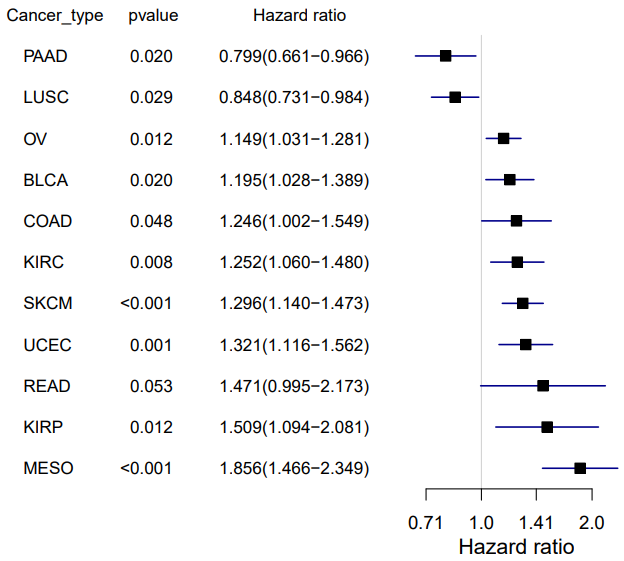


**Table S1. All the datasets used in the manuscript.**

1. The Cancer Genome Atlas (TCGA) datasets download from UCSC Xena.

| Cancer | Samples |
| --- | --- |
| Bladder Cancer (BLCA) | 430 |
| Colon Cancer (COAD) | 512 |
| Endometrioid Cancer (UCEC) | 583 |
| Kidney Clear Cell Carcinoma (KIRC) | 607 |
| Kidney Papillary Cell Carcinoma (KIRP) | 321 |
| Lung Squamous Cell Carcinoma (LUSC) | 550 |
| Melanoma (SKCM) | 472 |
| Mesothelioma (MESO) | 86 |
| Ovarian Cancer (OV) | 308 |
| Pancreatic Cancer (PAAD) | 182 |
| Rectal Cancer (READ) | 177 |
| Pan-Cancer (PANCAN) | 10535 |
| GTEX | 9783 |
| 1. The Gene Expression Omnibus (GEO) OC datasets |  |

| GSE ID | platform | samples |
| --- | --- | --- |
| GSE18520 | Affymetrix HG-U133 Plus 2.0 (GPL570) | 63 |
| GSE26193 | Affymetrix HG-U133 Plus 2.0 (GPL570) | 107 |
| GSE30161 | Affymetrix HG-U133 Plus 2.0 (GPL570) | 58 |
| GSE63885 | Affymetrix HG-U133 Plus 2.0 (GPL570) | 75 |
| GSE9891 | Affymetrix HG-U133 Plus 2.0 (GPL570) | 278 |
| GSE54388 | Affymetrix HG-U133 Plus 2.0 (GPL570) | 22 |
| GSE14764 | Affymetrix Human Genome U133A Array (GPL96) | 80 |
| GSE23554 | Affymetrix Human Genome U133A Array (GPL96) | 28 |
| GSE26712 | Affymetrix Human Genome U133A Array (GPL96) | 195 |

1. Oncomine OC datasets

| cancer type | samples | website |
| --- | --- | --- |
| Lu Ovarian cancer | 5 normal samples and 45 tumor samples | Oncomine (http://www.oncomine.org) |
| Yoshihara Ovarian cancer | 10 normal samples and 43 tumor samples | Oncomine |
| Bonome Ovarian cancer | 10 normal samples and 185 tumor samples | Oncomine |
| Hendrix Ovarian cancer | 4 normal samples and 99 tumor samples | Oncomine |

**Table S2. List of marker information for C0 verse C6, Related to Figure 3E.**

| Gene | p_val | avg_logFC | pct.1 | pct.2 | p_val_adj |
| --- | --- | --- | --- | --- | --- |
| SST | 1.58E-05 | 2.587291 | 0.119 | 0.006 | 0.267892 |
| H19 | 5.52E-18 | 1.620609 | 0.47 | 0.094 | 9.37E-14 |
| RPS18 | 1.16E-37 | 1.426501 | 0.853 | 0.428 | 1.96E-33 |
| RPS19 | 2.82E-39 | 1.38382 | 0.895 | 0.484 | 4.78E-35 |
| CXCL1 | 8.67E-06 | 1.359555 | 0.201 | 0.057 | 0.147202 |
| RPS12 | 2.33E-35 | 1.339089 | 0.873 | 0.591 | 3.95E-31 |
| PIGR | 4.09E-07 | 1.288278 | 0.164 | 0.013 | 0.006946 |
| SEMA3B | 3.59E-13 | 1.274227 | 0.33 | 0.044 | 6.09E-09 |
| GPX3 | 1.48E-08 | 1.274082 | 0.286 | 0.082 | 0.000251 |
| CRABP2 | 1.15E-14 | 1.258536 | 0.391 | 0.075 | 1.95E-10 |
| RPLP0 | 6.42E-32 | 1.210788 | 0.846 | 0.465 | 1.09E-27 |
| SPON1 | 1.68E-16 | 1.201226 | 0.399 | 0.05 | 2.85E-12 |
| COL18A1 | 5.71E-18 | 1.192084 | 0.512 | 0.119 | 9.70E-14 |
| RPL12 | 1.06E-30 | 1.19118 | 0.851 | 0.497 | 1.80E-26 |
| RPL37 | 8.02E-33 | 1.174942 | 0.86 | 0.553 | 1.36E-28 |
| RPS16 | 1.21E-34 | 1.161064 | 0.888 | 0.509 | 2.05E-30 |
| MUC5B | 2.03E-07 | 1.149978 | 0.189 | 0.025 | 0.003453 |
| RPS21 | 9.51E-30 | 1.149343 | 0.843 | 0.478 | 1.62E-25 |
| ATHL1 | 2.94E-07 | 1.147231 | 0.251 | 0.069 | 0.004993 |
| FTL | 9.44E-24 | 1.137156 | 0.744 | 0.321 | 1.60E-19 |
| RPS5 | 1.27E-22 | 1.115168 | 0.754 | 0.377 | 2.16E-18 |
| RPSA | 1.03E-21 | 1.110857 | 0.664 | 0.239 | 1.75E-17 |
| MSLN | 1.45E-21 | 1.107832 | 0.621 | 0.182 | 2.46E-17 |
| RPL27A | 7.19E-32 | 1.103506 | 0.885 | 0.56 | 1.22E-27 |
| PDZK1IP1 | 5.61E-11 | 1.094211 | 0.323 | 0.069 | 9.53E-07 |
| GPNMB | 8.63E-07 | 1.092371 | 0.136 | 0 | 0.014655 |
| RPL3 | 5.30E-25 | 1.088004 | 0.783 | 0.377 | 9.00E-21 |
| BCAT1 | 4.10E-10 | 1.073668 | 0.283 | 0.05 | 6.96E-06 |
| RPL26 | 3.92E-19 | 1.072809 | 0.646 | 0.27 | 6.67E-15 |
| XBP1 | 3.80E-09 | 1.068843 | 0.338 | 0.107 | 6.45E-05 |
| RPLP1 | 8.14E-36 | 1.062979 | 0.935 | 0.698 | 1.38E-31 |
| SNHG5 | 4.07E-17 | 1.058972 | 0.504 | 0.126 | 6.92E-13 |
| LY6E | 1.03E-18 | 1.056148 | 0.472 | 0.088 | 1.75E-14 |
| GAS5 | 2.37E-23 | 1.048265 | 0.712 | 0.252 | 4.02E-19 |
| RPS2 | 1.01E-24 | 1.047771 | 0.847 | 0.497 | 1.72E-20 |
| MARCKSL1 | 3.85E-16 | 1.046994 | 0.409 | 0.063 | 6.53E-12 |
| TMSB4X | 1.41E-19 | 1.03079 | 0.768 | 0.421 | 2.40E-15 |
| LRRC75A.AS1 | 1.87E-18 | 1.029823 | 0.586 | 0.189 | 3.17E-14 |
| TM4SF1 | 2.50E-11 | 1.027456 | 0.456 | 0.17 | 4.25E-07 |
| PAX8 | 2.00E-13 | 1.021967 | 0.399 | 0.088 | 3.39E-09 |
| RPL32 | 1.90E-27 | 1.021053 | 0.851 | 0.528 | 3.23E-23 |
| RPL18A | 1.42E-21 | 1.020042 | 0.726 | 0.34 | 2.41E-17 |
| RPL28 | 4.34E-27 | 1.018822 | 0.826 | 0.421 | 7.38E-23 |
| RPLP2 | 9.07E-31 | 1.0172 | 0.919 | 0.629 | 1.54E-26 |
| RPL35 | 4.46E-23 | 1.015362 | 0.794 | 0.434 | 7.57E-19 |
| MYC | 1.62E-10 | 1.013875 | 0.337 | 0.082 | 2.75E-06 |
| KRT17 | 1.98E-07 | 1.012849 | 0.31 | 0.107 | 0.00337 |
| LCN2 | 5.55E-12 | 1.009437 | 0.486 | 0.17 | 9.42E-08 |
| LDHB | 2.75E-14 | 1.009003 | 0.481 | 0.151 | 4.68E-10 |
| RPL13 | 7.43E-24 | 1.003522 | 0.851 | 0.547 | 1.26E-19 |
| RPL8 | 2.71E-30 | 0.998704 | 0.891 | 0.629 | 4.60E-26 |
| RPL27 | 5.54E-22 | 0.996626 | 0.737 | 0.333 | 9.42E-18 |
| LRIG1 | 1.95E-08 | 0.994416 | 0.325 | 0.101 | 0.000331 |
| RPL11 | 1.46E-23 | 0.98735 | 0.852 | 0.61 | 2.47E-19 |
| RPL37A | 1.43E-27 | 0.978944 | 0.875 | 0.591 | 2.43E-23 |
| RPL5 | 1.28E-20 | 0.975598 | 0.733 | 0.365 | 2.17E-16 |
| EEF1G | 1.64E-20 | 0.973515 | 0.698 | 0.289 | 2.78E-16 |
| C3 | 1.16E-10 | 0.973399 | 0.428 | 0.151 | 1.96E-06 |
| RPS14 | 4.20E-25 | 0.971812 | 0.865 | 0.597 | 7.13E-21 |
| RPS15A | 7.26E-19 | 0.971515 | 0.558 | 0.157 | 1.23E-14 |
| RPS11 | 5.11E-28 | 0.969795 | 0.9 | 0.56 | 8.69E-24 |
| SPOCK2 | 4.28E-08 | 0.968838 | 0.256 | 0.057 | 0.000726 |
| RPS28 | 4.05E-23 | 0.967922 | 0.733 | 0.296 | 6.89E-19 |
| CXCL17 | 1.48E-07 | 0.956454 | 0.191 | 0.025 | 0.002519 |
| KRT7 | 1.00E-18 | 0.955086 | 0.638 | 0.226 | 1.70E-14 |
| RPL7A | 1.89E-20 | 0.953845 | 0.82 | 0.604 | 3.22E-16 |
| IFITM1 | 7.85E-15 | 0.953689 | 0.61 | 0.258 | 1.33E-10 |
| RPL36 | 3.03E-20 | 0.952009 | 0.721 | 0.327 | 5.15E-16 |
| RPS9 | 1.97E-22 | 0.950558 | 0.805 | 0.421 | 3.35E-18 |
| CAPN13 | 2.56E-06 | 0.940635 | 0.137 | 0.006 | 0.043533 |
| XIST | 5.68E-15 | 0.940533 | 0.627 | 0.264 | 9.64E-11 |
| RPS29 | 8.57E-19 | 0.935845 | 0.727 | 0.39 | 1.46E-14 |
| SLC25A6 | 1.09E-18 | 0.932571 | 0.644 | 0.258 | 1.85E-14 |
| GNB2L1 | 5.72E-22 | 0.929675 | 0.828 | 0.484 | 9.71E-18 |
| EEF1B2 | 2.56E-15 | 0.928813 | 0.589 | 0.258 | 4.34E-11 |
| RPS15 | 2.86E-19 | 0.926398 | 0.742 | 0.346 | 4.86E-15 |
| RPS6 | 7.44E-18 | 0.926321 | 0.833 | 0.616 | 1.26E-13 |
| RPS23 | 9.75E-20 | 0.925183 | 0.749 | 0.403 | 1.66E-15 |
| RPS24 | 2.51E-21 | 0.924248 | 0.768 | 0.428 | 4.26E-17 |
| RPS8 | 3.47E-24 | 0.923961 | 0.886 | 0.667 | 5.89E-20 |
| RPL19 | 5.85E-23 | 0.914093 | 0.832 | 0.459 | 9.93E-19 |
| RPL18 | 3.64E-23 | 0.906425 | 0.837 | 0.497 | 6.18E-19 |
| RPL30 | 5.15E-23 | 0.904858 | 0.87 | 0.604 | 8.76E-19 |
| LYPD1 | 3.73E-10 | 0.901536 | 0.249 | 0.025 | 6.33E-06 |
| RPL14 | 2.29E-19 | 0.899472 | 0.738 | 0.371 | 3.88E-15 |
| MMP7 | 3.60E-05 | 0.89616 | 0.173 | 0.044 | 0.611702 |
| VEGFA | 1.16E-12 | 0.89372 | 0.431 | 0.113 | 1.97E-08 |
| RPS3A | 6.69E-17 | 0.890358 | 0.572 | 0.208 | 1.14E-12 |
| RPL35A | 2.90E-21 | 0.888064 | 0.804 | 0.459 | 4.92E-17 |
| RPL29 | 8.12E-19 | 0.884603 | 0.738 | 0.415 | 1.38E-14 |
| IFITM3 | 4.12E-18 | 0.883747 | 0.807 | 0.459 | 6.99E-14 |
| PABPC1 | 1.66E-15 | 0.881744 | 0.656 | 0.321 | 2.81E-11 |
| RPS25 | 1.60E-16 | 0.876949 | 0.636 | 0.283 | 2.72E-12 |
| ASS1 | 1.90E-17 | 0.873123 | 0.411 | 0.05 | 3.22E-13 |
| GRN | 3.51E-12 | 0.872921 | 0.342 | 0.063 | 5.96E-08 |
| DEFB1 | 5.07E-06 | 0.865328 | 0.13 | 0.006 | 0.086063 |
| DHCR24 | 5.59E-13 | 0.86404 | 0.357 | 0.063 | 9.50E-09 |
| RPL6 | 8.94E-13 | 0.863677 | 0.527 | 0.208 | 1.52E-08 |
| RPL13A | 2.69E-14 | 0.863023 | 0.588 | 0.245 | 4.56E-10 |
| TPM4 | 4.64E-12 | 0.858456 | 0.381 | 0.088 | 7.87E-08 |
| FOLR1 | 1.86E-12 | 0.857138 | 0.373 | 0.082 | 3.15E-08 |
| LMO4 | 3.38E-11 | 0.854278 | 0.338 | 0.069 | 5.74E-07 |
| RPSAP58 | 7.23E-17 | 0.847122 | 0.64 | 0.289 | 1.23E-12 |
| RPL23 | 1.34E-18 | 0.84646 | 0.783 | 0.421 | 2.28E-14 |
| TFPI2 | 3.84E-05 | 0.845116 | 0.132 | 0.019 | 0.651807 |
| LYNX1 | 1.10E-05 | 0.841892 | 0.133 | 0.013 | 0.186183 |
| SNHG8 | 7.26E-11 | 0.840847 | 0.428 | 0.151 | 1.23E-06 |
| RPL4 | 1.77E-15 | 0.840842 | 0.688 | 0.371 | 3.00E-11 |
| RPS27A | 2.57E-20 | 0.839146 | 0.791 | 0.459 | 4.37E-16 |
| TFF3 | 0.068542 | 0.837529 | 0.131 | 0.082 | 1 |
| CXCL2 | 0.000125 | 0.830061 | 0.253 | 0.113 | 1 |
| NME1 | 9.23E-11 | 0.827195 | 0.327 | 0.075 | 1.57E-06 |
| S100A14 | 5.54E-10 | 0.824203 | 0.244 | 0.025 | 9.42E-06 |
| WFDC2 | 3.77E-19 | 0.820332 | 0.872 | 0.572 | 6.40E-15 |
| RPL13AP20 | 6.38E-18 | 0.818063 | 0.616 | 0.239 | 1.08E-13 |
| CHI3L1 | 2.10E-08 | 0.816521 | 0.181 | 0.006 | 0.000356 |
| RPS3 | 7.56E-20 | 0.811199 | 0.864 | 0.597 | 1.28E-15 |
| ATP5G3 | 2.46E-12 | 0.808104 | 0.563 | 0.258 | 4.17E-08 |
| APOL1 | 1.15E-06 | 0.806231 | 0.291 | 0.107 | 0.019466 |
| RPL10 | 1.03E-13 | 0.805752 | 0.577 | 0.252 | 1.75E-09 |
| TPT1 | 2.63E-17 | 0.804281 | 0.895 | 0.698 | 4.47E-13 |
| PPA1 | 1.82E-09 | 0.802582 | 0.34 | 0.094 | 3.10E-05 |
| RPL7 | 5.08E-19 | 0.800396 | 0.702 | 0.333 | 8.63E-15 |
| NACA | 2.43E-16 | 0.798527 | 0.715 | 0.384 | 4.12E-12 |
| LDLR | 8.20E-07 | 0.791535 | 0.215 | 0.05 | 0.013931 |
| BHLHE41 | 6.18E-07 | 0.788648 | 0.354 | 0.151 | 0.010492 |
| DAPL1 | 5.56E-09 | 0.787335 | 0.207 | 0.013 | 9.45E-05 |
| TMEM205 | 3.81E-12 | 0.779844 | 0.431 | 0.138 | 6.47E-08 |
| KLK8 | 2.17E-10 | 0.767404 | 0.386 | 0.119 | 3.69E-06 |
| RPL10A | 1.92E-17 | 0.767021 | 0.753 | 0.409 | 3.25E-13 |
| S100A16 | 7.94E-09 | 0.76336 | 0.296 | 0.075 | 0.000135 |
| ST6GAL1 | 2.05E-08 | 0.759872 | 0.267 | 0.057 | 0.000349 |
| C6orf48 | 1.97E-13 | 0.752576 | 0.573 | 0.233 | 3.34E-09 |
| RPS4X | 3.17E-15 | 0.752262 | 0.828 | 0.635 | 5.38E-11 |
| THSD4 | 0.000204 | 0.751941 | 0.188 | 0.069 | 1 |
| RPL23A | 1.13E-13 | 0.750225 | 0.638 | 0.308 | 1.92E-09 |
| HSPA1A | 1.42E-09 | 0.749443 | 0.441 | 0.176 | 2.42E-05 |
| RPS20 | 4.37E-18 | 0.748195 | 0.788 | 0.453 | 7.42E-14 |
| RPL9 | 1.40E-13 | 0.745233 | 0.722 | 0.447 | 2.38E-09 |
| SLC40A1 | 5.01E-06 | 0.744796 | 0.364 | 0.182 | 0.085127 |
| EEF1D | 4.96E-14 | 0.741939 | 0.668 | 0.327 | 8.42E-10 |
| DUSP2 | 8.24E-05 | 0.74117 | 0.247 | 0.107 | 1 |
| ADIRF | 7.61E-10 | 0.739214 | 0.388 | 0.126 | 1.29E-05 |
| KLK7 | 1.46E-07 | 0.73773 | 0.289 | 0.088 | 0.002475 |
| PTGS1 | 1.11E-07 | 0.737001 | 0.247 | 0.057 | 0.001879 |
| LDHA | 2.15E-11 | 0.734015 | 0.478 | 0.176 | 3.65E-07 |
| WT1 | 3.71E-06 | 0.730759 | 0.249 | 0.082 | 0.062952 |
| SPRY1 | 7.15E-06 | 0.729621 | 0.214 | 0.063 | 0.121426 |
| UBA52 | 1.87E-14 | 0.727618 | 0.778 | 0.509 | 3.18E-10 |
| RPS14P3 | 4.88E-14 | 0.725391 | 0.711 | 0.409 | 8.29E-10 |
| TMEM147 | 6.75E-11 | 0.723376 | 0.347 | 0.082 | 1.15E-06 |
| TNFAIP2 | 2.35E-13 | 0.722456 | 0.598 | 0.239 | 4.00E-09 |
| SLC44A2 | 2.11E-08 | 0.719748 | 0.304 | 0.088 | 0.000358 |
| RPL24 | 6.16E-15 | 0.71919 | 0.756 | 0.453 | 1.05E-10 |
| CDKN1C | 5.60E-06 | 0.718954 | 0.336 | 0.151 | 0.095169 |
| KLK6 | 1.04E-07 | 0.718906 | 0.225 | 0.044 | 0.001764 |
| EIF3E | 1.05E-07 | 0.715238 | 0.322 | 0.113 | 0.00179 |
| SERPINH1 | 5.85E-09 | 0.71495 | 0.259 | 0.044 | 9.94E-05 |
| NPM1 | 1.17E-12 | 0.713516 | 0.572 | 0.258 | 1.98E-08 |
| SOD2 | 1.44E-07 | 0.711849 | 0.352 | 0.132 | 0.002442 |
| ARPC5 | 2.78E-08 | 0.710939 | 0.278 | 0.069 | 0.000472 |
| C2orf88 | 1.33E-08 | 0.709265 | 0.253 | 0.044 | 0.000226 |
| RBM3 | 2.35E-10 | 0.70802 | 0.374 | 0.113 | 3.99E-06 |
| PSME1 | 2.24E-10 | 0.706408 | 0.402 | 0.126 | 3.81E-06 |
| NDRG1 | 1.24E-07 | 0.70542 | 0.4 | 0.17 | 0.002103 |
| EIF3K | 1.06E-09 | 0.704532 | 0.436 | 0.176 | 1.79E-05 |
| RNASET2 | 7.58E-13 | 0.704099 | 0.51 | 0.176 | 1.29E-08 |
| FAU | 1.93E-16 | 0.704044 | 0.832 | 0.566 | 3.28E-12 |
| EGR3 | 0.000602 | 0.703971 | 0.199 | 0.088 | 1 |
| CFI | 2.24E-08 | 0.701679 | 0.253 | 0.05 | 0.000381 |
| UBB | 2.02E-07 | 0.700412 | 0.325 | 0.113 | 0.003439 |
| ASRGL1 | 6.71E-13 | 0.699997 | 0.533 | 0.189 | 1.14E-08 |
| DEPTOR | 1.41E-07 | 0.699972 | 0.242 | 0.057 | 0.002396 |
| ST3GAL1 | 1.18E-07 | 0.699636 | 0.185 | 0.019 | 0.002005 |
| DDIT4 | 5.51E-07 | 0.694268 | 0.474 | 0.264 | 0.00936 |
| MAL2 | 5.43E-10 | 0.691124 | 0.477 | 0.195 | 9.22E-06 |
| KLK10 | 2.32E-07 | 0.690937 | 0.299 | 0.101 | 0.003939 |
| MYADM | 1.02E-08 | 0.689349 | 0.399 | 0.157 | 0.000173 |
| RASSF7 | 1.76E-07 | 0.685145 | 0.369 | 0.145 | 0.002984 |
| ADAM28 | 2.33E-05 | 0.68034 | 0.126 | 0.013 | 0.395231 |
| CDH6 | 1.83E-07 | 0.677831 | 0.151 | 0 | 0.003113 |
| ISG15 | 7.74E-06 | 0.6775 | 0.268 | 0.094 | 0.131483 |
| NRBP2 | 1.58E-05 | 0.677099 | 0.173 | 0.038 | 0.267616 |
| GAPDH | 3.67E-18 | 0.675502 | 0.88 | 0.629 | 6.23E-14 |
| TPI1 | 3.78E-12 | 0.673968 | 0.654 | 0.352 | 6.41E-08 |
| NHP2 | 4.51E-11 | 0.673725 | 0.36 | 0.088 | 7.67E-07 |
| RPL19P12 | 2.13E-12 | 0.673305 | 0.43 | 0.132 | 3.62E-08 |
| RPL34 | 4.81E-15 | 0.672802 | 0.778 | 0.478 | 8.17E-11 |
| RPL22 | 1.39E-10 | 0.672765 | 0.416 | 0.132 | 2.37E-06 |
| RNASE1 | 7.48E-05 | 0.672275 | 0.207 | 0.075 | 1 |
| EMP1 | 8.17E-08 | 0.670999 | 0.246 | 0.05 | 0.001388 |
| GOLGA8A | 1.77E-05 | 0.666755 | 0.206 | 0.063 | 0.300936 |
| VTCN1 | 1.85E-06 | 0.66643 | 0.128 | 0 | 0.03142 |
| FGF18 | 9.53E-07 | 0.664598 | 0.189 | 0.031 | 0.016195 |
| PTMA | 5.58E-11 | 0.659419 | 0.64 | 0.346 | 9.47E-07 |
| JUP | 4.07E-09 | 0.659332 | 0.411 | 0.151 | 6.91E-05 |
| STAT1 | 2.33E-05 | 0.659312 | 0.193 | 0.057 | 0.39498 |
| RPS7 | 1.78E-11 | 0.658098 | 0.664 | 0.403 | 3.02E-07 |
| EEF2 | 1.58E-12 | 0.658039 | 0.656 | 0.327 | 2.69E-08 |
| AK2 | 4.74E-10 | 0.656874 | 0.281 | 0.044 | 8.05E-06 |
| SMARCD3 | 7.57E-06 | 0.654823 | 0.148 | 0.019 | 0.128641 |
| MT1E | 9.65E-07 | 0.653912 | 0.231 | 0.057 | 0.016388 |
| TNNT1 | 1.08E-07 | 0.652104 | 0.296 | 0.094 | 0.001837 |
| RBBP7 | 1.86E-06 | 0.652066 | 0.317 | 0.132 | 0.031635 |
| THBS1 | 0.005052 | 0.651069 | 0.126 | 0.05 | 1 |
| NPR1 | 3.96E-09 | 0.649367 | 0.207 | 0.013 | 6.72E-05 |
| TMEM176A | 1.94E-05 | 0.647659 | 0.178 | 0.044 | 0.329032 |
| XAF1 | 4.47E-06 | 0.647078 | 0.144 | 0.013 | 0.07593 |
| MYL9 | 5.79E-08 | 0.646623 | 0.295 | 0.082 | 0.000983 |
| RPS13 | 1.99E-13 | 0.643198 | 0.714 | 0.365 | 3.37E-09 |
| DUT | 5.02E-09 | 0.642588 | 0.358 | 0.113 | 8.52E-05 |
| ZNF83 | 1.40E-05 | 0.639993 | 0.238 | 0.082 | 0.238158 |
| COL27A1 | 4.51E-05 | 0.638338 | 0.13 | 0.019 | 0.766122 |
| RPLP0P2 | 9.41E-13 | 0.638019 | 0.54 | 0.22 | 1.60E-08 |
| MYRF | 2.81E-05 | 0.63777 | 0.135 | 0.019 | 0.477296 |
| CYC1 | 1.79E-11 | 0.635457 | 0.457 | 0.164 | 3.04E-07 |
| THAP9.AS1 | 1.37E-06 | 0.63294 | 0.207 | 0.044 | 0.023235 |
| MDK | 8.94E-16 | 0.632654 | 0.674 | 0.308 | 1.52E-11 |
| GPT2 | 4.26E-05 | 0.629665 | 0.141 | 0.025 | 0.723061 |
| SCD | 1.57E-05 | 0.628785 | 0.119 | 0.006 | 0.266452 |
| ZFP36L1 | 1.95E-12 | 0.624862 | 0.743 | 0.421 | 3.30E-08 |
| KRT19 | 5.58E-13 | 0.623599 | 0.723 | 0.39 | 9.47E-09 |
| TRIB1 | 4.06E-06 | 0.622732 | 0.315 | 0.126 | 0.069005 |
| EIF3H | 3.35E-09 | 0.621984 | 0.472 | 0.201 | 5.69E-05 |
| RAB34 | 1.70E-08 | 0.616913 | 0.272 | 0.057 | 0.000288 |
| THEM6 | 3.35E-06 | 0.616416 | 0.207 | 0.05 | 0.056948 |
| CD81 | 3.93E-11 | 0.61481 | 0.773 | 0.503 | 6.68E-07 |
| SNHG3 | 2.87E-10 | 0.614743 | 0.386 | 0.119 | 4.88E-06 |
| PHLDA2 | 4.78E-08 | 0.608845 | 0.294 | 0.082 | 0.000812 |
| NFIA | 2.02E-07 | 0.608786 | 0.222 | 0.044 | 0.003428 |
| NDUFS8 | 1.12E-09 | 0.608731 | 0.4 | 0.132 | 1.90E-05 |
| MYO1C | 6.03E-09 | 0.607024 | 0.236 | 0.031 | 0.000102 |
| LSM4 | 1.54E-07 | 0.606826 | 0.301 | 0.101 | 0.002623 |
| PEA15 | 1.61E-08 | 0.606542 | 0.286 | 0.075 | 0.000274 |
| MUC1 | 4.41E-11 | 0.606044 | 0.756 | 0.421 | 7.49E-07 |
| PPFIBP1 | 7.05E-06 | 0.603931 | 0.181 | 0.038 | 0.119823 |
| RAPGEF3 | 3.00E-06 | 0.602721 | 0.169 | 0.025 | 0.050988 |
| BST2 | 6.25E-12 | 0.602584 | 0.414 | 0.107 | 1.06E-07 |
| IP6K2 | 7.58E-09 | 0.601246 | 0.37 | 0.126 | 0.000129 |
| RAN | 9.03E-09 | 0.59778 | 0.467 | 0.208 | 0.000153 |
| PRSS8 | 1.74E-08 | 0.597598 | 0.207 | 0.019 | 0.000296 |
| DANCR | 4.11E-08 | 0.596822 | 0.306 | 0.088 | 0.000699 |
| SLC25A38 | 2.77E-05 | 0.595088 | 0.177 | 0.044 | 0.471025 |
| C19orf48 | 1.18E-08 | 0.594528 | 0.188 | 0.006 | 0.0002 |
| CCNG1 | 9.48E-07 | 0.592833 | 0.205 | 0.044 | 0.016108 |
| LYPD6B | 3.77E-06 | 0.592321 | 0.19 | 0.038 | 0.064107 |
| IGFBP2 | 2.41E-06 | 0.58997 | 0.241 | 0.075 | 0.040996 |
| C2 | 3.56E-07 | 0.589493 | 0.157 | 0.006 | 0.006052 |
| RPL38 | 1.43E-17 | 0.588819 | 0.732 | 0.371 | 2.43E-13 |
| MPZL2 | 2.99E-07 | 0.585862 | 0.241 | 0.057 | 0.005086 |
| WTAP | 3.39E-06 | 0.585848 | 0.228 | 0.063 | 0.057652 |
| HLA.DMB | 2.44E-05 | 0.585431 | 0.186 | 0.05 | 0.414121 |
| SORL1 | 2.55E-05 | 0.585363 | 0.346 | 0.176 | 0.433164 |
| HMGA1 | 2.64E-11 | 0.58509 | 0.422 | 0.138 | 4.49E-07 |
| METRNL | 7.41E-06 | 0.584796 | 0.216 | 0.063 | 0.125882 |
| LSM7 | 1.38E-08 | 0.584624 | 0.31 | 0.082 | 0.000234 |
| FAM69B | 2.36E-05 | 0.584378 | 0.125 | 0.013 | 0.40144 |
| IMPDH2 | 3.56E-09 | 0.583351 | 0.396 | 0.145 | 6.05E-05 |
| CLDN10 | 2.08E-06 | 0.583212 | 0.215 | 0.05 | 0.035272 |
| CHCHD10 | 2.36E-08 | 0.582668 | 0.384 | 0.145 | 0.0004 |
| HLA.DMA | 0.000123 | 0.582535 | 0.231 | 0.094 | 1 |
| BIN1 | 1.55E-05 | 0.581131 | 0.13 | 0.013 | 0.263596 |
| SPDEF | 3.94E-06 | 0.579965 | 0.121 | 0 | 0.066853 |
| VASN | 1.29E-06 | 0.5795 | 0.231 | 0.063 | 0.021982 |
| GLTSCR2 | 1.10E-08 | 0.578223 | 0.477 | 0.22 | 0.000186 |
| NAP1L1 | 8.06E-08 | 0.577811 | 0.511 | 0.277 | 0.001369 |
| GPI | 3.55E-13 | 0.577536 | 0.43 | 0.101 | 6.03E-09 |
| MST1 | 0.000704 | 0.577213 | 0.142 | 0.044 | 1 |
| GPRC5C | 1.12E-06 | 0.574262 | 0.235 | 0.063 | 0.019062 |
| CTSC | 7.99E-07 | 0.573108 | 0.173 | 0.019 | 0.013574 |
| SNRPD2 | 1.78E-11 | 0.571148 | 0.525 | 0.201 | 3.02E-07 |
| RPL31 | 6.34E-19 | 0.568796 | 0.863 | 0.642 | 1.08E-14 |
| PON2 | 4.72E-07 | 0.568637 | 0.269 | 0.082 | 0.008018 |
| TMA7 | 6.55E-10 | 0.567437 | 0.37 | 0.107 | 1.11E-05 |
| RPL15 | 6.91E-13 | 0.566886 | 0.81 | 0.572 | 1.17E-08 |
| C8orf59 | 4.03E-08 | 0.56669 | 0.291 | 0.075 | 0.000685 |
| C1R | 2.45E-06 | 0.566162 | 0.202 | 0.044 | 0.041598 |
| TOMM20 | 3.85E-07 | 0.565436 | 0.368 | 0.157 | 0.00654 |
| ARF4 | 5.33E-07 | 0.563688 | 0.459 | 0.226 | 0.009057 |
| C19orf33 | 6.55E-12 | 0.563298 | 0.496 | 0.182 | 1.11E-07 |
| LAPTM4B | 2.70E-10 | 0.563267 | 0.507 | 0.214 | 4.58E-06 |
| LRRC41 | 8.04E-05 | 0.561025 | 0.219 | 0.082 | 1 |
| PIP5K1A | 9.13E-05 | 0.559483 | 0.143 | 0.031 | 1 |
| PABPC4 | 7.27E-08 | 0.559392 | 0.368 | 0.138 | 0.001235 |
| FBL | 1.66E-12 | 0.558666 | 0.415 | 0.107 | 2.81E-08 |
| NAA50 | 3.80E-06 | 0.554822 | 0.205 | 0.05 | 0.064559 |
| TUBB | 3.60E-09 | 0.554687 | 0.367 | 0.126 | 6.11E-05 |
| RGL3 | 9.55E-05 | 0.553195 | 0.18 | 0.057 | 1 |
| RBBP6 | 7.02E-06 | 0.552965 | 0.284 | 0.107 | 0.119323 |
| BAG3 | 0.000215 | 0.551316 | 0.294 | 0.145 | 1 |
| HSPA6 | 0.023301 | 0.550602 | 0.11 | 0.05 | 1 |
| YBX1 | 1.27E-09 | 0.550238 | 0.356 | 0.101 | 2.16E-05 |
| CP | 7.20E-06 | 0.549971 | 0.356 | 0.164 | 0.122346 |
| CRTAP | 1.46E-06 | 0.549362 | 0.214 | 0.05 | 0.024846 |
| ACTG1 | 1.82E-13 | 0.547523 | 0.849 | 0.585 | 3.09E-09 |
| SH3YL1 | 3.48E-06 | 0.545771 | 0.238 | 0.069 | 0.059114 |
| FRMD4B | 5.08E-05 | 0.545127 | 0.169 | 0.044 | 0.862533 |
| ATP5J | 1.18E-09 | 0.544886 | 0.379 | 0.119 | 2.00E-05 |
| NAMPT | 1.15E-05 | 0.544225 | 0.212 | 0.063 | 0.196081 |
| FAM136A | 1.48E-07 | 0.543428 | 0.186 | 0.019 | 0.002515 |
| LINC01320 | 0.016743 | 0.543408 | 0.112 | 0.05 | 1 |
| CDIPT | 3.32E-06 | 0.542344 | 0.209 | 0.05 | 0.056389 |
| FAM43A | 5.80E-05 | 0.541639 | 0.117 | 0.013 | 0.984446 |
| MRPL3 | 1.19E-06 | 0.541338 | 0.267 | 0.082 | 0.020176 |
| FOSB | 5.98E-06 | 0.54114 | 0.625 | 0.447 | 0.101504 |
| KCNK15 | 1.48E-05 | 0.540769 | 0.185 | 0.044 | 0.251242 |
| CFB | 1.01E-07 | 0.540182 | 0.421 | 0.176 | 0.001713 |
| TTC14 | 0.000462 | 0.539973 | 0.146 | 0.044 | 1 |
| PSAT1 | 4.01E-06 | 0.539606 | 0.144 | 0.013 | 0.068048 |
| FKBP4 | 5.08E-08 | 0.53839 | 0.372 | 0.138 | 0.000863 |
| RSL1D1 | 2.09E-06 | 0.537249 | 0.238 | 0.069 | 0.035563 |
| RPL41 | 1.81E-08 | 0.536869 | 0.322 | 0.101 | 0.000308 |
| HPN | 1.97E-08 | 0.536523 | 0.206 | 0.019 | 0.000334 |
| MRPS15 | 2.31E-08 | 0.535365 | 0.206 | 0.019 | 0.000392 |
| AKT1 | 1.43E-07 | 0.535357 | 0.272 | 0.075 | 0.002431 |
| PODXL | 1.27E-06 | 0.533833 | 0.249 | 0.069 | 0.021578 |
| GLS | 2.48E-05 | 0.53354 | 0.147 | 0.025 | 0.422016 |
| TMEM160 | 2.02E-05 | 0.533286 | 0.152 | 0.025 | 0.343639 |
| TSC22D4 | 2.74E-05 | 0.532615 | 0.238 | 0.088 | 0.465504 |
| SEPHS1 | 1.74E-07 | 0.531382 | 0.174 | 0.013 | 0.002963 |
| MCM7 | 4.09E-06 | 0.531239 | 0.238 | 0.069 | 0.069548 |
| KLHL24 | 4.51E-06 | 0.530976 | 0.132 | 0.006 | 0.076658 |
| COX6C | 7.47E-09 | 0.530531 | 0.521 | 0.258 | 0.000127 |
| F3 | 0.00067 | 0.529954 | 0.104 | 0.019 | 1 |
| FLJ44635 | 2.34E-08 | 0.529724 | 0.563 | 0.327 | 0.000397 |
| TGIF2 | 6.78E-06 | 0.529194 | 0.178 | 0.038 | 0.115122 |
| FAM20C | 1.07E-06 | 0.528928 | 0.146 | 0.006 | 0.01813 |
| P4HB | 4.67E-13 | 0.528348 | 0.514 | 0.164 | 7.94E-09 |
| STARD7 | 9.57E-08 | 0.525705 | 0.263 | 0.063 | 0.001625 |
| TPD52 | 3.67E-08 | 0.525627 | 0.223 | 0.031 | 0.000623 |
| PLAUR | 0.001941 | 0.525497 | 0.101 | 0.025 | 1 |
| TNS1 | 4.05E-05 | 0.52421 | 0.141 | 0.025 | 0.688142 |
| AMOTL2 | 1.97E-06 | 0.522862 | 0.26 | 0.082 | 0.033451 |
| CYBRD1 | 6.40E-05 | 0.522283 | 0.14 | 0.025 | 1 |
| SLC34A2 | 4.47E-08 | 0.522206 | 0.306 | 0.082 | 0.00076 |
| LRRC8A | 8.02E-05 | 0.520537 | 0.135 | 0.025 | 1 |
| SPIN1 | 4.58E-06 | 0.520368 | 0.28 | 0.101 | 0.077866 |
| CDK4 | 2.33E-08 | 0.520026 | 0.258 | 0.05 | 0.000397 |
| KIAA1551 | 1.01E-06 | 0.51885 | 0.278 | 0.088 | 0.017072 |
| KLK5 | 5.35E-06 | 0.518513 | 0.131 | 0.006 | 0.090918 |
| TBL1XR1 | 1.02E-05 | 0.518294 | 0.216 | 0.063 | 0.173295 |
| SLC52A2 | 3.68E-06 | 0.517854 | 0.189 | 0.038 | 0.062426 |
| STARD10 | 5.80E-06 | 0.516804 | 0.206 | 0.05 | 0.098471 |
| PRKCI | 6.11E-07 | 0.516449 | 0.221 | 0.044 | 0.010386 |
| MIR205HG | 8.36E-05 | 0.515888 | 0.115 | 0.013 | 1 |
| UQCRB | 6.88E-08 | 0.515634 | 0.472 | 0.226 | 0.001169 |
| IDH1 | 1.51E-06 | 0.515075 | 0.153 | 0.013 | 0.025689 |
| MGST1 | 1.07E-05 | 0.5145 | 0.188 | 0.044 | 0.182287 |
| GORASP2 | 0.000206 | 0.514014 | 0.179 | 0.063 | 1 |
| BZW2 | 2.36E-07 | 0.513572 | 0.214 | 0.038 | 0.00401 |
| IGFBP3 | 0.000483 | 0.513486 | 0.107 | 0.019 | 1 |
| DGAT1 | 5.89E-06 | 0.512385 | 0.184 | 0.038 | 0.10008 |
| POLR1D | 2.11E-05 | 0.512268 | 0.283 | 0.119 | 0.358 |
| KRTCAP3 | 9.91E-05 | 0.511974 | 0.221 | 0.088 | 1 |
| SHMT2 | 2.23E-06 | 0.511701 | 0.238 | 0.069 | 0.037846 |
| TNKS1BP1 | 2.71E-06 | 0.511213 | 0.219 | 0.057 | 0.046011 |
| CD63 | 2.02E-09 | 0.51102 | 0.747 | 0.478 | 3.44E-05 |
| TXNL4A | 1.52E-08 | 0.510857 | 0.321 | 0.088 | 0.000258 |
| MZT2B | 1.13E-10 | 0.510616 | 0.489 | 0.195 | 1.92E-06 |
| LZTS3 | 5.18E-06 | 0.510079 | 0.175 | 0.031 | 0.08804 |
| RPS27 | 7.67E-12 | 0.509969 | 0.716 | 0.409 | 1.30E-07 |
| ZYX | 1.17E-07 | 0.509816 | 0.301 | 0.094 | 0.001982 |
| BCAM | 4.72E-13 | 0.508693 | 0.384 | 0.075 | 8.02E-09 |
| OGT | 2.33E-05 | 0.508487 | 0.327 | 0.151 | 0.396185 |
| B4GALNT4 | 1.06E-05 | 0.507969 | 0.111 | 0 | 0.180889 |
| PPP1CA | 2.53E-07 | 0.507414 | 0.333 | 0.119 | 0.004297 |
| SLC38A1 | 1.34E-07 | 0.50709 | 0.307 | 0.094 | 0.002277 |
| NDUFB9 | 1.70E-08 | 0.506746 | 0.542 | 0.27 | 0.000288 |
| FAM171A1 | 5.03E-05 | 0.506279 | 0.119 | 0.013 | 0.853851 |
| EPS8L1 | 1.05E-08 | 0.506155 | 0.29 | 0.069 | 0.000178 |
| RBM26 | 0.000325 | 0.505109 | 0.174 | 0.063 | 1 |
| RNF7 | 6.31E-06 | 0.504273 | 0.275 | 0.101 | 0.107128 |
| SEL1L3 | 4.34E-06 | 0.503962 | 0.153 | 0.019 | 0.073687 |
| VWA1 | 2.03E-05 | 0.50341 | 0.252 | 0.094 | 0.345328 |
| FTH1 | 1.34E-07 | 0.503141 | 0.807 | 0.56 | 0.002283 |
| LCN12 | 2.43E-05 | 0.503131 | 0.126 | 0.013 | 0.412827 |
| IFI44 | 1.87E-05 | 0.502598 | 0.193 | 0.05 | 0.317286 |
| ARL4C | 1.37E-09 | 0.501244 | 0.285 | 0.05 | 2.32E-05 |
| POLD4 | 1.96E-07 | 0.500997 | 0.242 | 0.057 | 0.003337 |
| NONO | 2.90E-07 | 0.500785 | 0.359 | 0.145 | 0.004933 |
| HADH | 5.68E-06 | 0.500602 | 0.211 | 0.057 | 0.096464 |
| RGS10 | 3.03E-05 | 0.50005 | 0.206 | 0.069 | 0.5139 |
| APOA1BP | 3.15E-07 | 0.497879 | 0.248 | 0.063 | 0.005356 |
| ASNS | 0.000146 | 0.497249 | 0.13 | 0.025 | 1 |
| TMSB10 | 4.35E-12 | 0.49724 | 0.885 | 0.648 | 7.39E-08 |
| CFL1 | 2.89E-11 | 0.496252 | 0.722 | 0.434 | 4.90E-07 |
| GALNT6 | 1.60E-05 | 0.496161 | 0.14 | 0.019 | 0.272341 |
| ETV4 | 3.95E-05 | 0.495669 | 0.152 | 0.031 | 0.67149 |
| DPYSL3 | 1.52E-05 | 0.494951 | 0.174 | 0.038 | 0.258982 |
| PVRL2 | 4.32E-07 | 0.49408 | 0.283 | 0.088 | 0.007331 |
| MIF | 5.43E-08 | 0.493676 | 0.764 | 0.522 | 0.000922 |
| LAMB2 | 9.69E-06 | 0.493239 | 0.252 | 0.088 | 0.164574 |
| SAT2 | 0.000168 | 0.493051 | 0.194 | 0.069 | 1 |
| ATP2B1 | 0.000261 | 0.492897 | 0.217 | 0.088 | 1 |
| CBR1 | 8.23E-05 | 0.492595 | 0.156 | 0.038 | 1 |
| HNRNPA3 | 2.59E-05 | 0.491898 | 0.354 | 0.176 | 0.439807 |
| RBBP8 | 0.000635 | 0.491411 | 0.142 | 0.044 | 1 |
| ARPP19 | 7.53E-06 | 0.490946 | 0.19 | 0.044 | 0.12787 |
| PATL1 | 1.54E-05 | 0.490336 | 0.141 | 0.019 | 0.261434 |
| VNN1 | 0.035219 | 0.488054 | 0.104 | 0.05 | 1 |
| RCC2 | 4.38E-07 | 0.487442 | 0.264 | 0.075 | 0.007448 |
| RDH10 | 0.003086 | 0.487167 | 0.127 | 0.044 | 1 |
| CD200 | 3.86E-06 | 0.486941 | 0.189 | 0.038 | 0.06554 |
| SMG1 | 0.000129 | 0.485826 | 0.18 | 0.057 | 1 |
| ARC | 0.000702 | 0.485402 | 0.212 | 0.094 | 1 |
| NDUFA3 | 5.49E-06 | 0.485045 | 0.267 | 0.094 | 0.093334 |
| APEX1 | 4.90E-09 | 0.48475 | 0.314 | 0.075 | 8.32E-05 |
| LGALS3BP | 9.26E-11 | 0.484624 | 0.675 | 0.371 | 1.57E-06 |
| EIF5A | 1.88E-07 | 0.484447 | 0.356 | 0.138 | 0.003195 |
| LTA4H | 7.04E-07 | 0.484311 | 0.202 | 0.038 | 0.011963 |
| PDHA1 | 5.24E-06 | 0.482979 | 0.196 | 0.044 | 0.089014 |
| AIFM1 | 0.000283 | 0.482551 | 0.169 | 0.057 | 1 |
| HEXA | 1.96E-06 | 0.482406 | 0.209 | 0.044 | 0.033266 |
| DOK5 | 2.78E-05 | 0.479602 | 0.126 | 0.013 | 0.471391 |
| IL4I1 | 0.00222 | 0.47938 | 0.11 | 0.031 | 1 |
| CPSF6 | 0.000118 | 0.479204 | 0.16 | 0.044 | 1 |
| CGGBP1 | 0.000268 | 0.478508 | 0.188 | 0.069 | 1 |
| NSG1 | 6.89E-06 | 0.477902 | 0.149 | 0.019 | 0.117071 |
| BIRC6 | 0.000468 | 0.477364 | 0.117 | 0.025 | 1 |
| APRT | 7.75E-07 | 0.477204 | 0.336 | 0.132 | 0.013158 |
| PITRM1 | 9.75E-05 | 0.476524 | 0.179 | 0.057 | 1 |
| LZTS2 | 0.00035 | 0.476339 | 0.185 | 0.069 | 1 |
| MCL1 | 2.00E-06 | 0.475696 | 0.696 | 0.465 | 0.034043 |
| SUSD3 | 3.86E-07 | 0.47501 | 0.201 | 0.031 | 0.006555 |
| ARGLU1 | 1.36E-06 | 0.474827 | 0.419 | 0.201 | 0.023079 |
| CKAP4 | 3.16E-05 | 0.474593 | 0.186 | 0.05 | 0.537386 |
| MTF2 | 0.000554 | 0.474576 | 0.116 | 0.025 | 1 |
| PCBD1 | 2.49E-07 | 0.473959 | 0.384 | 0.164 | 0.004228 |
| KIAA1324 | 0.00072 | 0.473617 | 0.114 | 0.025 | 1 |
| EID1 | 7.48E-07 | 0.473395 | 0.293 | 0.094 | 0.012709 |
| ARL4D | 8.19E-06 | 0.473208 | 0.193 | 0.044 | 0.139065 |
| SCNN1A | 2.83E-08 | 0.473019 | 0.442 | 0.195 | 0.00048 |
| CCNB1IP1 | 1.29E-06 | 0.472787 | 0.167 | 0.019 | 0.021911 |
| GPAA1 | 3.88E-07 | 0.471699 | 0.347 | 0.138 | 0.006587 |
| APH1A | 6.76E-07 | 0.470524 | 0.338 | 0.138 | 0.011477 |
| AP2S1 | 9.57E-06 | 0.470236 | 0.222 | 0.069 | 0.162539 |
| ARHGEF3 | 0.000421 | 0.469566 | 0.107 | 0.019 | 1 |
| GJA1 | 5.54E-05 | 0.469146 | 0.141 | 0.025 | 0.941705 |
| TGM2 | 0.00185 | 0.468707 | 0.151 | 0.057 | 1 |
| CYCS | 1.51E-09 | 0.468418 | 0.431 | 0.151 | 2.56E-05 |
| ENO1 | 6.06E-10 | 0.468282 | 0.706 | 0.465 | 1.03E-05 |
| GOLGA8B | 0.001052 | 0.468245 | 0.206 | 0.094 | 1 |
| SF3B1 | 1.47E-05 | 0.466892 | 0.405 | 0.208 | 0.250065 |
| PHPT1 | 1.17E-05 | 0.466827 | 0.396 | 0.208 | 0.198173 |
| PABPC1L | 0.000576 | 0.466534 | 0.115 | 0.025 | 1 |
| FCGRT | 8.57E-07 | 0.46626 | 0.184 | 0.025 | 0.01455 |
| DCAF13 | 0.000251 | 0.46622 | 0.164 | 0.05 | 1 |
| SHARPIN | 1.41E-05 | 0.465715 | 0.232 | 0.075 | 0.238704 |
| CD151 | 2.00E-09 | 0.465592 | 0.454 | 0.195 | 3.40E-05 |
| ATP6V1B1 | 1.70E-08 | 0.465067 | 0.286 | 0.063 | 0.000289 |
| MRPL27 | 2.90E-05 | 0.464184 | 0.222 | 0.075 | 0.492723 |
| KLF4 | 1.15E-07 | 0.462751 | 0.52 | 0.258 | 0.001949 |
| IFI16 | 0.000428 | 0.462067 | 0.2 | 0.082 | 1 |
| KLHDC2 | 0.000111 | 0.461488 | 0.174 | 0.05 | 1 |
| CTSF | 4.14E-06 | 0.461334 | 0.156 | 0.019 | 0.070376 |
| TUG1 | 7.24E-05 | 0.459994 | 0.221 | 0.082 | 1 |
| RDH11 | 9.35E-06 | 0.459649 | 0.146 | 0.019 | 0.15879 |
| HLA.DPA1 | 5.80E-05 | 0.458958 | 0.249 | 0.101 | 0.985039 |
| MPST | 2.84E-05 | 0.458646 | 0.157 | 0.031 | 0.483094 |
| DDT | 5.88E-08 | 0.458233 | 0.307 | 0.088 | 0.000998 |
| NCBP2 | 1.35E-05 | 0.458218 | 0.206 | 0.057 | 0.228709 |
| EEF1A1 | 1.36E-07 | 0.458144 | 0.519 | 0.296 | 0.002317 |
| PRDX4 | 0.000253 | 0.458115 | 0.284 | 0.145 | 1 |
| H2AFZ | 1.73E-07 | 0.457982 | 0.426 | 0.195 | 0.002945 |
| APOL2 | 0.000275 | 0.457273 | 0.132 | 0.031 | 1 |
| CAV1 | 0.00016 | 0.456854 | 0.109 | 0.013 | 1 |
| SEPHS2 | 2.36E-06 | 0.456756 | 0.181 | 0.031 | 0.040052 |
| UBE2G2 | 4.79E-05 | 0.456694 | 0.247 | 0.094 | 0.81408 |
| ATP5D | 3.10E-06 | 0.456618 | 0.291 | 0.113 | 0.052589 |
| MAGT1 | 2.98E-05 | 0.456558 | 0.175 | 0.044 | 0.506236 |
| MYL6B | 5.16E-07 | 0.456465 | 0.27 | 0.075 | 0.008771 |
| WAC | 2.21E-05 | 0.456381 | 0.299 | 0.126 | 0.375844 |
| UGT2B7 | 7.99E-06 | 0.456224 | 0.149 | 0.019 | 0.135697 |
| SRSF6 | 1.28E-06 | 0.456157 | 0.346 | 0.138 | 0.021757 |
| GDI2 | 7.98E-05 | 0.455943 | 0.244 | 0.101 | 1 |
| TIMM13 | 1.25E-05 | 0.455832 | 0.278 | 0.107 | 0.212746 |
| CX3CL1 | 0.000122 | 0.455071 | 0.11 | 0.013 | 1 |
| PRDX2 | 7.21E-07 | 0.454937 | 0.267 | 0.082 | 0.012244 |
| KIF1C | 3.51E-05 | 0.453991 | 0.154 | 0.031 | 0.596996 |
| SNHG15 | 0.000187 | 0.453435 | 0.154 | 0.044 | 1 |
| RHOF | 1.38E-05 | 0.453423 | 0.132 | 0.013 | 0.233854 |
| TMEM54 | 5.64E-05 | 0.453341 | 0.138 | 0.025 | 0.957212 |
| INF2 | 2.31E-08 | 0.453245 | 0.281 | 0.063 | 0.000392 |
| RTF1 | 7.10E-07 | 0.453158 | 0.263 | 0.075 | 0.012055 |
| ZFAS1 | 1.46E-10 | 0.453125 | 0.593 | 0.277 | 2.47E-06 |
| EBP | 2.16E-06 | 0.452541 | 0.211 | 0.05 | 0.036667 |
| WDR83OS | 8.39E-11 | 0.452445 | 0.541 | 0.233 | 1.43E-06 |
| HSPG2 | 1.07E-05 | 0.451879 | 0.216 | 0.063 | 0.181055 |
| UQCR11 | 4.57E-07 | 0.449981 | 0.398 | 0.17 | 0.007767 |
| EIF3M | 2.74E-06 | 0.449241 | 0.335 | 0.145 | 0.046459 |
| S100A1 | 4.95E-11 | 0.449164 | 0.256 | 0.019 | 8.41E-07 |
| SRSF11 | 1.75E-09 | 0.448431 | 0.469 | 0.189 | 2.98E-05 |
| MSX1 | 0.082308 | 0.448382 | 0.358 | 0.283 | 1 |
| GSTK1 | 1.69E-06 | 0.44807 | 0.389 | 0.17 | 0.028694 |
| STXBP6 | 2.01E-05 | 0.447873 | 0.13 | 0.013 | 0.340719 |
| BTNL9 | 6.29E-05 | 0.447696 | 0.106 | 0.006 | 1 |
| TOP1MT | 6.48E-06 | 0.446867 | 0.116 | 0 | 0.110143 |
| NPDC1 | 3.59E-06 | 0.446429 | 0.28 | 0.101 | 0.060962 |
| CALD1 | 8.51E-08 | 0.446419 | 0.194 | 0.019 | 0.001446 |
| ZNF704 | 0.000123 | 0.446243 | 0.131 | 0.025 | 1 |
| MZT2A | 3.76E-07 | 0.445691 | 0.288 | 0.094 | 0.006395 |
| QARS | 1.36E-06 | 0.445657 | 0.293 | 0.101 | 0.023024 |
| LONRF2 | 0.001875 | 0.445643 | 0.131 | 0.044 | 1 |
| NT5DC2 | 7.52E-06 | 0.445236 | 0.149 | 0.019 | 0.127802 |
| CDK2AP1 | 6.28E-06 | 0.445138 | 0.299 | 0.119 | 0.106705 |
| FAM107A | 6.69E-05 | 0.444339 | 0.177 | 0.05 | 1 |
| KLK11 | 1.08E-06 | 0.444293 | 0.237 | 0.063 | 0.018303 |
| RARRES2 | 0.000196 | 0.444167 | 0.312 | 0.164 | 1 |
| FSCN1 | 1.72E-05 | 0.443859 | 0.183 | 0.044 | 0.291371 |
| GSTO1 | 6.26E-06 | 0.443382 | 0.2 | 0.05 | 0.10634 |
| FOXQ1 | 0.000222 | 0.443361 | 0.104 | 0.013 | 1 |
| SH3BP2 | 0.000133 | 0.44197 | 0.159 | 0.044 | 1 |
| COA6 | 6.02E-05 | 0.441816 | 0.169 | 0.044 | 1 |
| NDUFB7 | 9.72E-06 | 0.441119 | 0.457 | 0.252 | 0.165113 |
| TRIM22 | 0.00092 | 0.441082 | 0.12 | 0.031 | 1 |
| NACA2 | 1.80E-09 | 0.440881 | 0.464 | 0.189 | 3.06E-05 |
| RPS26 | 4.83E-06 | 0.440583 | 0.306 | 0.126 | 0.081997 |
| ABRACL | 1.99E-05 | 0.439654 | 0.17 | 0.038 | 0.337437 |
| PTP4A3 | 2.10E-05 | 0.439502 | 0.179 | 0.044 | 0.356852 |
| CTDSP1 | 1.45E-07 | 0.439433 | 0.238 | 0.05 | 0.002455 |
| BGN | 0.000151 | 0.439177 | 0.12 | 0.019 | 1 |
| SLC25A5 | 6.46E-08 | 0.438683 | 0.459 | 0.214 | 0.001096 |
| C12orf75 | 0.003447 | 0.438354 | 0.115 | 0.038 | 1 |
| RBM17 | 0.000229 | 0.438328 | 0.299 | 0.151 | 1 |
| CHD3 | 1.87E-07 | 0.43799 | 0.278 | 0.075 | 0.003182 |
| LOC284454 | 0.000348 | 0.437835 | 0.351 | 0.201 | 1 |
| NDUFB4 | 1.09E-06 | 0.436861 | 0.337 | 0.132 | 0.018456 |
| SSR2 | 1.97E-08 | 0.436586 | 0.405 | 0.157 | 0.000335 |
| TAOK1 | 0.002655 | 0.436439 | 0.135 | 0.05 | 1 |
| IFRD2 | 1.94E-05 | 0.435467 | 0.141 | 0.019 | 0.329754 |
| LRP1 | 0.000596 | 0.435134 | 0.116 | 0.025 | 1 |
| ZC3H12A | 0.000115 | 0.434738 | 0.251 | 0.107 | 1 |
| PSMB1 | 2.67E-06 | 0.43406 | 0.407 | 0.195 | 0.04537 |
| LGALS1 | 1.23E-07 | 0.43394 | 0.248 | 0.057 | 0.002097 |
| NRIP1 | 1.51E-05 | 0.433139 | 0.156 | 0.025 | 0.257134 |
| MRPL4 | 2.30E-08 | 0.432843 | 0.258 | 0.05 | 0.00039 |
| MRPL52 | 7.98E-06 | 0.432665 | 0.223 | 0.069 | 0.135583 |
| BLVRB | 1.95E-06 | 0.432603 | 0.223 | 0.057 | 0.033092 |
| SND1 | 2.29E-07 | 0.432036 | 0.247 | 0.057 | 0.003885 |
| SNHG12 | 0.000121 | 0.430809 | 0.295 | 0.145 | 1 |
| MRPL11 | 2.60E-06 | 0.43071 | 0.227 | 0.063 | 0.044231 |
| IER2 | 0.001012 | 0.430685 | 0.607 | 0.447 | 1 |
| AIMP1 | 0.001734 | 0.430516 | 0.14 | 0.05 | 1 |
| OSTC | 3.06E-06 | 0.430405 | 0.259 | 0.082 | 0.05201 |
| SURF4 | 1.19E-07 | 0.429607 | 0.264 | 0.063 | 0.00203 |
| MIR3654 | 2.25E-06 | 0.429102 | 0.248 | 0.075 | 0.03815 |
| TXNDC17 | 5.43E-06 | 0.428957 | 0.212 | 0.057 | 0.092299 |
| GNPNAT1 | 7.90E-05 | 0.428737 | 0.116 | 0.013 | 1 |
| CD2AP | 0.000223 | 0.428632 | 0.164 | 0.05 | 1 |
| ATP5EP2 | 6.61E-06 | 0.426869 | 0.323 | 0.145 | 0.112316 |
| TIMP2 | 4.77E-06 | 0.425543 | 0.165 | 0.025 | 0.081059 |
| C8orf4 | 1.38E-05 | 0.425195 | 0.198 | 0.05 | 0.234833 |
| PNPLA2 | 2.69E-05 | 0.42486 | 0.231 | 0.082 | 0.456562 |
| ALDH18A1 | 7.73E-05 | 0.424828 | 0.158 | 0.038 | 1 |
| SUSD2 | 0.000106 | 0.424748 | 0.11 | 0.013 | 1 |
| FAM20A | 4.17E-05 | 0.424569 | 0.11 | 0.006 | 0.708936 |
| KDM6B | 0.001774 | 0.424484 | 0.165 | 0.069 | 1 |
| ASPH | 1.95E-05 | 0.423737 | 0.151 | 0.025 | 0.330829 |
| TUBA1C | 8.16E-06 | 0.423594 | 0.379 | 0.182 | 0.138643 |
| LTBP4 | 0.000573 | 0.423282 | 0.165 | 0.057 | 1 |
| ANAPC11 | 6.44E-08 | 0.423109 | 0.416 | 0.17 | 0.001094 |
| MRPS25 | 1.72E-05 | 0.422372 | 0.18 | 0.044 | 0.292184 |
| ID1 | 0.007649 | 0.421962 | 0.331 | 0.22 | 1 |
| GRAMD3 | 9.72E-05 | 0.421655 | 0.175 | 0.05 | 1 |
| XPO1 | 1.39E-06 | 0.421327 | 0.39 | 0.176 | 0.023653 |
| SDC2 | 1.20E-05 | 0.420803 | 0.121 | 0.006 | 0.203437 |
| MRPL12 | 1.79E-08 | 0.420703 | 0.281 | 0.069 | 0.000304 |
| MAGED2 | 6.95E-06 | 0.420414 | 0.319 | 0.132 | 0.118071 |
| RAB24 | 0.001261 | 0.420217 | 0.116 | 0.031 | 1 |
| EIF3B | 2.71E-07 | 0.419813 | 0.327 | 0.113 | 0.004602 |
| AUP1 | 2.29E-07 | 0.419807 | 0.293 | 0.088 | 0.003884 |
| CDC42SE1 | 3.11E-05 | 0.419718 | 0.199 | 0.057 | 0.528125 |
| SLC48A1 | 0.000109 | 0.419436 | 0.162 | 0.044 | 1 |
| PTRF | 3.81E-05 | 0.418933 | 0.143 | 0.025 | 0.64641 |
| SRP72 | 0.00014 | 0.418292 | 0.159 | 0.044 | 1 |
| NUPR1 | 9.94E-05 | 0.418226 | 0.291 | 0.138 | 1 |
| AHCY | 1.82E-07 | 0.41723 | 0.315 | 0.101 | 0.00309 |
| AQP5 | 1.60E-06 | 0.417056 | 0.154 | 0.013 | 0.027191 |
| TNKS2 | 0.007654 | 0.416473 | 0.126 | 0.05 | 1 |
| S100A2 | 0.002437 | 0.415774 | 0.13 | 0.044 | 1 |
| FBXO32 | 0.000161 | 0.415577 | 0.151 | 0.038 | 1 |
| COX5B | 1.04E-07 | 0.415376 | 0.615 | 0.358 | 0.001763 |
| TARS | 0.000337 | 0.414988 | 0.131 | 0.031 | 1 |
| MFSD3 | 0.000109 | 0.414821 | 0.121 | 0.019 | 1 |
| MIEN1 | 2.72E-05 | 0.414463 | 0.225 | 0.075 | 0.461421 |
| MUC16 | 1.26E-05 | 0.413291 | 0.468 | 0.277 | 0.213247 |
| UPP1 | 0.000198 | 0.412891 | 0.105 | 0.013 | 1 |
| SRSF1 | 1.32E-08 | 0.412786 | 0.377 | 0.132 | 0.000225 |
| LSM8 | 0.000145 | 0.412448 | 0.233 | 0.094 | 1 |
| PIEZO1 | 0.000429 | 0.411899 | 0.119 | 0.025 | 1 |
| TIMP1 | 1.18E-05 | 0.411338 | 0.501 | 0.289 | 0.201076 |
| RASD1 | 0.003789 | 0.411188 | 0.164 | 0.075 | 1 |
| USP53 | 0.007678 | 0.410781 | 0.272 | 0.17 | 1 |
| PARP10 | 0.012368 | 0.410633 | 0.191 | 0.107 | 1 |
| PRKCD | 0.000218 | 0.410255 | 0.201 | 0.075 | 1 |
| FAM195B | 8.05E-07 | 0.41018 | 0.273 | 0.082 | 0.013667 |
| DDOST | 3.27E-05 | 0.410063 | 0.164 | 0.038 | 0.554865 |
| CDC42EP4 | 6.91E-06 | 0.409668 | 0.283 | 0.107 | 0.117339 |
| ADM | 0.000279 | 0.408677 | 0.146 | 0.038 | 1 |
| FAM162A | 3.29E-05 | 0.408225 | 0.258 | 0.101 | 0.558134 |
| PTP4A2 | 1.09E-06 | 0.408118 | 0.341 | 0.132 | 0.018449 |
| SLBP | 0.000923 | 0.407632 | 0.19 | 0.082 | 1 |
| AHR | 6.62E-05 | 0.406993 | 0.283 | 0.126 | 1 |
| SNHG19 | 2.61E-05 | 0.406737 | 0.186 | 0.05 | 0.442838 |
| COX5A | 9.03E-07 | 0.406686 | 0.423 | 0.201 | 0.015342 |
| CNDP2 | 4.15E-06 | 0.406602 | 0.299 | 0.113 | 0.07044 |
| S100A6 | 0.068525 | 0.406147 | 0.846 | 0.755 | 1 |
| AHSA2 | 0.005923 | 0.406054 | 0.159 | 0.075 | 1 |
| ZNF503 | 1.50E-06 | 0.405823 | 0.432 | 0.214 | 0.025491 |
| PPP1R12C | 0.000258 | 0.405293 | 0.133 | 0.031 | 1 |
| COTL1 | 0.00155 | 0.405247 | 0.104 | 0.025 | 1 |
| ILF2 | 1.21E-06 | 0.405182 | 0.32 | 0.119 | 0.020473 |
| SRM | 2.22E-05 | 0.40456 | 0.138 | 0.019 | 0.377071 |
| LPP | 6.03E-06 | 0.40436 | 0.202 | 0.05 | 0.102452 |
| EMC10 | 1.29E-05 | 0.40431 | 0.216 | 0.063 | 0.218685 |
| PSMC2 | 0.003303 | 0.404119 | 0.162 | 0.069 | 1 |
| EIF3L | 3.45E-06 | 0.403523 | 0.356 | 0.151 | 0.058669 |
| ZMYM2 | 0.000408 | 0.403024 | 0.194 | 0.075 | 1 |
| SLC25A39 | 9.67E-07 | 0.402465 | 0.265 | 0.082 | 0.016419 |
| BMP1 | 9.24E-05 | 0.402078 | 0.102 | 0.006 | 1 |
| CARS2 | 7.56E-05 | 0.401752 | 0.126 | 0.019 | 1 |
| NDUFA10 | 6.46E-07 | 0.401356 | 0.246 | 0.063 | 0.010979 |
| COPA | 0.000303 | 0.4009 | 0.251 | 0.113 | 1 |
| PLCD3 | 8.44E-05 | 0.400726 | 0.135 | 0.025 | 1 |
| CPSF1 | 0.002853 | 0.400042 | 0.214 | 0.107 | 1 |
| PCNA | 0.001555 | 0.399559 | 0.195 | 0.088 | 1 |
| ACSL5 | 0.000907 | 0.399488 | 0.109 | 0.025 | 1 |
| SIAH2 | 0.001079 | 0.399468 | 0.138 | 0.044 | 1 |
| MEST | 1.83E-05 | 0.399358 | 0.13 | 0.013 | 0.311559 |
| S100A4 | 0.000195 | 0.399152 | 0.344 | 0.182 | 1 |
| DUSP23 | 6.42E-05 | 0.398843 | 0.289 | 0.132 | 1 |
| SMIM20 | 6.25E-05 | 0.39883 | 0.151 | 0.031 | 1 |
| BRWD1 | 0.001396 | 0.398391 | 0.142 | 0.05 | 1 |
| C9orf3 | 0.002746 | 0.398346 | 0.179 | 0.082 | 1 |
| PDHB | 9.69E-05 | 0.398236 | 0.184 | 0.057 | 1 |
| CLDN1 | 0.002582 | 0.39797 | 0.242 | 0.132 | 1 |
| NOL11 | 0.000274 | 0.397699 | 0.122 | 0.025 | 1 |
| PAICS | 8.04E-06 | 0.397689 | 0.221 | 0.063 | 0.136509 |
| C17orf89 | 7.31E-07 | 0.397172 | 0.257 | 0.069 | 0.012416 |
| TANK | 0.000637 | 0.396795 | 0.164 | 0.057 | 1 |
| ADI1 | 1.35E-05 | 0.396758 | 0.227 | 0.069 | 0.230102 |
| GAA | 0.000188 | 0.396192 | 0.159 | 0.044 | 1 |
| LETM1 | 0.001767 | 0.396175 | 0.133 | 0.044 | 1 |
| SNHG6 | 9.52E-08 | 0.395515 | 0.568 | 0.302 | 0.001617 |
| C9orf16 | 1.41E-07 | 0.395306 | 0.306 | 0.094 | 0.002399 |
| LOC440311 | 3.09E-05 | 0.394603 | 0.22 | 0.075 | 0.524408 |
| PLS3 | 1.17E-05 | 0.394105 | 0.123 | 0.006 | 0.198382 |
| CLIC5 | 0.00027 | 0.393757 | 0.162 | 0.05 | 1 |
| EP300 | 0.000253 | 0.393686 | 0.135 | 0.031 | 1 |
| FLNA | 2.16E-07 | 0.392624 | 0.39 | 0.164 | 0.003661 |
| CLUH | 0.000145 | 0.392059 | 0.132 | 0.025 | 1 |
| KCNQ1OT1 | 0.009101 | 0.391901 | 0.164 | 0.082 | 1 |
| PLXNA3 | 0.000497 | 0.391879 | 0.106 | 0.019 | 1 |
| STXBP3 | 0.004191 | 0.39177 | 0.141 | 0.057 | 1 |
| YIF1A | 4.23E-05 | 0.391502 | 0.194 | 0.057 | 0.718353 |
| METTL12 | 0.00491 | 0.391282 | 0.101 | 0.031 | 1 |
| SSR4 | 9.50E-09 | 0.391211 | 0.553 | 0.27 | 0.000161 |
| DCTPP1 | 3.69E-05 | 0.390744 | 0.175 | 0.044 | 0.626772 |
| SLC12A9 | 0.00131 | 0.390543 | 0.106 | 0.025 | 1 |
| CMPK1 | 1.22E-07 | 0.390257 | 0.233 | 0.044 | 0.002067 |
| UQCRQ | 6.84E-08 | 0.390167 | 0.454 | 0.201 | 0.001161 |
| FMR1 | 0.000826 | 0.390068 | 0.223 | 0.101 | 1 |
| NAPRT | 0.001775 | 0.389693 | 0.181 | 0.082 | 1 |
| MPV17 | 8.33E-05 | 0.389452 | 0.143 | 0.031 | 1 |
| ZNF655 | 0.000256 | 0.389288 | 0.167 | 0.05 | 1 |
| SSH3 | 0.002061 | 0.388042 | 0.121 | 0.038 | 1 |
| GLRX5 | 2.54E-06 | 0.387876 | 0.211 | 0.05 | 0.043218 |
| CLU | 0.001261 | 0.387789 | 0.732 | 0.61 | 1 |
| SF3A1 | 6.46E-06 | 0.387638 | 0.163 | 0.025 | 0.109739 |
| TMEM238 | 5.24E-08 | 0.386789 | 0.249 | 0.05 | 0.000889 |
| C1orf122 | 1.74E-05 | 0.386336 | 0.223 | 0.069 | 0.29539 |
| ATP6V1F | 9.13E-06 | 0.386232 | 0.299 | 0.119 | 0.155159 |
| C1S | 0.000275 | 0.385615 | 0.123 | 0.025 | 1 |
| ELOVL5 | 0.000637 | 0.385215 | 0.152 | 0.05 | 1 |
| MROH6 | 1.57E-05 | 0.3849 | 0.132 | 0.013 | 0.265887 |
| ODC1 | 5.93E-07 | 0.3846 | 0.435 | 0.208 | 0.010079 |
| RAMP1 | 8.21E-05 | 0.384534 | 0.136 | 0.025 | 1 |
| C7orf73 | 1.16E-06 | 0.384322 | 0.306 | 0.113 | 0.019634 |
| PLEKHH3 | 0.000367 | 0.384269 | 0.11 | 0.019 | 1 |
| SLC22A18 | 7.39E-05 | 0.383983 | 0.104 | 0.006 | 1 |
| CD276 | 0.000179 | 0.383288 | 0.117 | 0.019 | 1 |
| NFKBIZ | 9.25E-05 | 0.382259 | 0.458 | 0.264 | 1 |
| NT5C | 0.00049 | 0.381909 | 0.226 | 0.101 | 1 |
| MTR | 0.000547 | 0.381899 | 0.116 | 0.025 | 1 |
| POLR2K | 3.08E-08 | 0.381741 | 0.237 | 0.038 | 0.000523 |
| TPD52L1 | 2.93E-06 | 0.380811 | 0.222 | 0.057 | 0.049815 |
| ASAP2 | 0.015328 | 0.380594 | 0.133 | 0.063 | 1 |
| USF2 | 1.97E-07 | 0.380547 | 0.24 | 0.05 | 0.00335 |
| IFITM2 | 8.00E-05 | 0.378565 | 0.537 | 0.346 | 1 |
| OST4 | 2.83E-07 | 0.378462 | 0.467 | 0.214 | 0.004811 |
| PDXK | 6.64E-05 | 0.378195 | 0.247 | 0.094 | 1 |
| CBX3 | 9.60E-07 | 0.378178 | 0.359 | 0.157 | 0.016309 |
| COASY | 0.000108 | 0.378173 | 0.135 | 0.025 | 1 |
| TMEM139 | 2.27E-05 | 0.37709 | 0.127 | 0.013 | 0.385988 |
| NSUN5P1 | 6.88E-05 | 0.376913 | 0.13 | 0.019 | 1 |
| EIF1AX | 2.57E-06 | 0.376609 | 0.289 | 0.101 | 0.043612 |
| MRPL33 | 1.05E-06 | 0.376598 | 0.333 | 0.132 | 0.017916 |
| PCYOX1 | 7.40E-05 | 0.376379 | 0.116 | 0.013 | 1 |
| RPRD1A | 0.000198 | 0.376246 | 0.137 | 0.031 | 1 |
| ASAP1 | 9.24E-05 | 0.376213 | 0.125 | 0.019 | 1 |
| MKLN1 | 0.005879 | 0.375544 | 0.126 | 0.05 | 1 |
| MEX3C | 0.000962 | 0.375403 | 0.14 | 0.044 | 1 |
| LSM3 | 0.000947 | 0.375233 | 0.184 | 0.075 | 1 |
| TTC37 | 0.003055 | 0.37465 | 0.143 | 0.057 | 1 |
| PAFAH1B3 | 0.00085 | 0.374579 | 0.13 | 0.038 | 1 |
| ZNF146 | 0.000976 | 0.374535 | 0.168 | 0.063 | 1 |
| EML3 | 0.000239 | 0.373811 | 0.114 | 0.019 | 1 |
| FGFRL1 | 4.88E-06 | 0.373729 | 0.177 | 0.031 | 0.082835 |
| CSTF3 | 0.000196 | 0.373617 | 0.149 | 0.038 | 1 |
| ADAMTS1 | 5.56E-05 | 0.373087 | 0.181 | 0.05 | 0.944595 |
| TSPAN12 | 0.000559 | 0.372688 | 0.116 | 0.025 | 1 |
| CBX4 | 4.28E-06 | 0.372671 | 0.263 | 0.088 | 0.072666 |
| LINC01420 | 0.001656 | 0.372395 | 0.173 | 0.075 | 1 |
| POMGNT1 | 7.01E-05 | 0.37229 | 0.147 | 0.031 | 1 |
| ING4 | 0.000406 | 0.372283 | 0.131 | 0.031 | 1 |
| SOX9 | 0.001976 | 0.371824 | 0.191 | 0.088 | 1 |
| SEC62 | 5.14E-05 | 0.371699 | 0.277 | 0.119 | 0.873766 |
| HS6ST1 | 4.66E-05 | 0.37141 | 0.162 | 0.038 | 0.790736 |
| HIGD2A | 2.48E-06 | 0.371397 | 0.385 | 0.182 | 0.042161 |
| LEMD1 | 0.000164 | 0.371246 | 0.137 | 0.031 | 1 |
| TP53 | 6.07E-05 | 0.371157 | 0.147 | 0.031 | 1 |
| SUCLA2 | 0.000261 | 0.371117 | 0.114 | 0.019 | 1 |
| NPAS3 | 0.003262 | 0.37057 | 0.115 | 0.038 | 1 |
| BCL3 | 3.02E-05 | 0.370538 | 0.226 | 0.075 | 0.512742 |
| H1F0 | 0.000146 | 0.370359 | 0.249 | 0.107 | 1 |
| NET1 | 0.000199 | 0.370315 | 0.23 | 0.094 | 1 |
| LPIN1 | 0.003256 | 0.370231 | 0.116 | 0.038 | 1 |
| FAM110C | 0.000484 | 0.369606 | 0.119 | 0.025 | 1 |
| UHRF2 | 0.000878 | 0.369572 | 0.11 | 0.025 | 1 |
| RWDD1 | 9.36E-05 | 0.369427 | 0.153 | 0.038 | 1 |
| DNM1L | 0.000559 | 0.369413 | 0.136 | 0.038 | 1 |
| EHD1 | 0.000261 | 0.369274 | 0.233 | 0.101 | 1 |
| TFAP2A | 0.000613 | 0.36924 | 0.105 | 0.019 | 1 |
| SNTB1 | 2.52E-05 | 0.369063 | 0.102 | 0 | 0.427836 |
| PPP1R14B | 1.23E-06 | 0.368434 | 0.321 | 0.126 | 0.020826 |
| RPL22L1 | 3.03E-06 | 0.368326 | 0.302 | 0.113 | 0.051421 |
| SSR3 | 9.33E-07 | 0.368148 | 0.251 | 0.069 | 0.015848 |
| FLII | 0.000198 | 0.368102 | 0.227 | 0.094 | 1 |
| WDR74 | 0.013539 | 0.368022 | 0.123 | 0.057 | 1 |
| RTKN | 0.000166 | 0.368013 | 0.138 | 0.031 | 1 |
| POLR2F | 1.99E-06 | 0.36799 | 0.206 | 0.044 | 0.033837 |
| EML4 | 4.91E-05 | 0.367612 | 0.164 | 0.038 | 0.833642 |
| APOL6 | 0.00034 | 0.367521 | 0.11 | 0.019 | 1 |
| ZC3HAV1 | 0.011974 | 0.367237 | 0.133 | 0.063 | 1 |
| TRMT1 | 0.00028 | 0.367136 | 0.135 | 0.031 | 1 |
| FAM32A | 8.70E-07 | 0.367125 | 0.214 | 0.044 | 0.014773 |
| WDR91 | 0.000114 | 0.367017 | 0.135 | 0.025 | 1 |
| STX18 | 6.91E-05 | 0.366949 | 0.232 | 0.088 | 1 |
| CAND1 | 0.002523 | 0.366709 | 0.196 | 0.094 | 1 |
| MIER1 | 0.000357 | 0.366678 | 0.12 | 0.025 | 1 |
| CSAD | 0.000134 | 0.366186 | 0.169 | 0.05 | 1 |
| KLHL14 | 0.000214 | 0.36494 | 0.105 | 0.013 | 1 |
| DOK7 | 7.61E-05 | 0.364039 | 0.165 | 0.044 | 1 |
| CBX1 | 8.07E-05 | 0.363994 | 0.148 | 0.031 | 1 |
| RAD21 | 7.19E-05 | 0.363642 | 0.231 | 0.088 | 1 |
| VPS25 | 3.56E-05 | 0.363474 | 0.144 | 0.025 | 0.603923 |
| HLA.DRA | 0.000201 | 0.363464 | 0.479 | 0.289 | 1 |
| ATP5C1 | 3.73E-07 | 0.363162 | 0.399 | 0.17 | 0.006332 |
| CDC42EP1 | 0.000169 | 0.363009 | 0.178 | 0.057 | 1 |
| DNAJB12 | 0.000144 | 0.362284 | 0.16 | 0.044 | 1 |
| ALKBH7 | 3.54E-05 | 0.361205 | 0.243 | 0.094 | 0.601368 |
| PTCD3 | 0.000203 | 0.361001 | 0.193 | 0.069 | 1 |
| HNRNPR | 7.60E-05 | 0.3609 | 0.331 | 0.157 | 1 |
| SCAP | 0.003652 | 0.360584 | 0.142 | 0.057 | 1 |
| PIK3C2A | 0.000547 | 0.360468 | 0.178 | 0.063 | 1 |
| PPRC1 | 0.001461 | 0.36039 | 0.136 | 0.044 | 1 |
| DLD | 3.67E-05 | 0.36039 | 0.111 | 0.006 | 0.623029 |
| UAP1 | 0.010226 | 0.36036 | 0.111 | 0.044 | 1 |
| CERK | 0.000173 | 0.35955 | 0.119 | 0.019 | 1 |
| C1orf186 | 4.67E-05 | 0.359406 | 0.274 | 0.113 | 0.79347 |
| TMEM176B | 0.000394 | 0.359053 | 0.177 | 0.063 | 1 |
| NCKAP1 | 0.000251 | 0.358832 | 0.268 | 0.126 | 1 |
| SEPP1 | 7.45E-05 | 0.358545 | 0.28 | 0.126 | 1 |
| WDR1 | 0.000426 | 0.357264 | 0.217 | 0.094 | 1 |
| IMMP2L | 9.75E-05 | 0.3572 | 0.101 | 0.006 | 1 |
| PHF14 | 0.019651 | 0.357114 | 0.172 | 0.094 | 1 |
| ZNF195 | 0.000664 | 0.356846 | 0.115 | 0.025 | 1 |
| LOC101927318 | 8.38E-05 | 0.356544 | 0.102 | 0.006 | 1 |
| NCBP2.AS2 | 2.68E-06 | 0.356316 | 0.151 | 0.013 | 0.045501 |
| TRABD | 0.001555 | 0.356295 | 0.105 | 0.025 | 1 |
| NSUN2 | 0.000302 | 0.356245 | 0.169 | 0.057 | 1 |
| CLDN16 | 0.008725 | 0.355717 | 0.112 | 0.044 | 1 |
| DPH7 | 0.002913 | 0.355611 | 0.109 | 0.031 | 1 |
| AGR2 | 0.004078 | 0.355459 | 0.175 | 0.082 | 1 |
| HID1 | 0.000104 | 0.355037 | 0.152 | 0.038 | 1 |
| CDK5RAP3 | 2.14E-05 | 0.354977 | 0.314 | 0.138 | 0.364064 |
| PTDSS1 | 0.000111 | 0.354972 | 0.132 | 0.025 | 1 |
| NUCKS1 | 1.80E-07 | 0.354735 | 0.495 | 0.239 | 0.003059 |
| SNHG9 | 0.000617 | 0.35461 | 0.154 | 0.05 | 1 |
| NNMT | 0.000153 | 0.354584 | 0.223 | 0.094 | 1 |
| ITPR3 | 5.91E-05 | 0.354503 | 0.19 | 0.057 | 1 |
| CISD3 | 0.001596 | 0.354425 | 0.162 | 0.063 | 1 |
| ARHGAP23 | 0.004525 | 0.354343 | 0.121 | 0.044 | 1 |
| EXOSC5 | 5.14E-05 | 0.354287 | 0.119 | 0.013 | 0.873534 |
| FMOD | 3.50E-05 | 0.354157 | 0.122 | 0.013 | 0.594255 |
| CNN3 | 0.000601 | 0.353643 | 0.173 | 0.063 | 1 |
| SNCG | 8.00E-05 | 0.353588 | 0.125 | 0.019 | 1 |
| HDAC6 | 0.002392 | 0.353335 | 0.111 | 0.031 | 1 |
| GRINA | 1.67E-05 | 0.352669 | 0.377 | 0.189 | 0.284122 |
| HEXB | 0.00348 | 0.352551 | 0.135 | 0.05 | 1 |
| ZDHHC7 | 0.005488 | 0.352307 | 0.109 | 0.038 | 1 |
| CYB5A | 4.46E-05 | 0.352289 | 0.319 | 0.151 | 0.758331 |
| DBN1 | 0.000151 | 0.352078 | 0.12 | 0.019 | 1 |
| CAV2 | 0.00016 | 0.351883 | 0.13 | 0.025 | 1 |
| SIGMAR1 | 2.63E-05 | 0.351864 | 0.158 | 0.031 | 0.447063 |
| CXCL8 | 0.148338 | 0.351308 | 0.158 | 0.113 | 1 |
| EBPL | 0.000107 | 0.351164 | 0.2 | 0.069 | 1 |
| GPRC5A | 0.00062 | 0.350726 | 0.102 | 0.019 | 1 |
| RPS18P9 | 1.64E-11 | 0.35019 | 0.404 | 0.113 | 2.78E-07 |
| NDUFB11 | 4.62E-06 | 0.350114 | 0.36 | 0.17 | 0.078431 |
| EIF2S3 | 2.49E-06 | 0.349802 | 0.401 | 0.182 | 0.042357 |
| MTRNR2L3 | 0.000203 | 0.349695 | 0.147 | 0.038 | 1 |
| RNF31 | 0.000218 | 0.349607 | 0.116 | 0.019 | 1 |
| VKORC1 | 0.000588 | 0.349546 | 0.153 | 0.05 | 1 |
| RBPMS | 0.000493 | 0.348954 | 0.165 | 0.057 | 1 |
| AFG3L2 | 0.001173 | 0.34875 | 0.159 | 0.057 | 1 |
| WIPI2 | 6.76E-05 | 0.348238 | 0.222 | 0.082 | 1 |
| PUS1 | 0.001693 | 0.347945 | 0.114 | 0.031 | 1 |
| LTC4S | 0.001471 | 0.34789 | 0.105 | 0.025 | 1 |
| CTDSPL | 2.84E-06 | 0.347848 | 0.158 | 0.019 | 0.048212 |
| ALG3 | 0.001012 | 0.347681 | 0.119 | 0.031 | 1 |
| SCAMP3 | 8.00E-06 | 0.347554 | 0.215 | 0.063 | 0.135884 |
| TOMM22 | 0.001528 | 0.347081 | 0.115 | 0.031 | 1 |
| FNTA | 0.000216 | 0.346917 | 0.214 | 0.082 | 1 |
| TGFBR2 | 0.000123 | 0.346784 | 0.11 | 0.013 | 1 |
| LDOC1 | 0.000447 | 0.346717 | 0.167 | 0.057 | 1 |
| ATF5 | 5.94E-05 | 0.346638 | 0.128 | 0.019 | 1 |
| MUC20 | 0.005809 | 0.34636 | 0.18 | 0.088 | 1 |
| ATIC | 0.008362 | 0.346344 | 0.141 | 0.063 | 1 |
| TMEM14C | 0.000294 | 0.345947 | 0.353 | 0.195 | 1 |
| HOOK2 | 0.001244 | 0.345862 | 0.142 | 0.05 | 1 |
| PAWR | 0.000111 | 0.345266 | 0.111 | 0.013 | 1 |
| CDC123 | 9.05E-05 | 0.345203 | 0.168 | 0.044 | 1 |
| BAZ2A | 0.000173 | 0.345188 | 0.177 | 0.057 | 1 |
| RPS10 | 2.33E-06 | 0.344673 | 0.322 | 0.126 | 0.03954 |
| RYBP | 0.000108 | 0.34396 | 0.184 | 0.057 | 1 |
| AMD1 | 2.13E-05 | 0.343294 | 0.278 | 0.107 | 0.361802 |
| CITED4 | 0.001472 | 0.3427 | 0.258 | 0.138 | 1 |
| CLEC11A | 0.000198 | 0.342372 | 0.127 | 0.025 | 1 |
| DDX49 | 0.000578 | 0.342133 | 0.105 | 0.019 | 1 |
| ZNF358 | 2.22E-05 | 0.341759 | 0.126 | 0.013 | 0.376411 |
| PLEKHJ1 | 0.000555 | 0.341403 | 0.116 | 0.025 | 1 |
| ATOX1 | 0.000405 | 0.341004 | 0.27 | 0.132 | 1 |
| GUSB | 0.000305 | 0.339882 | 0.132 | 0.031 | 1 |
| HMGB1 | 6.03E-05 | 0.339452 | 0.322 | 0.157 | 1 |
| CHRAC1 | 6.09E-05 | 0.339224 | 0.14 | 0.025 | 1 |
| THY1 | 0.003781 | 0.339197 | 0.181 | 0.088 | 1 |
| UNC5B.AS1 | 6.06E-05 | 0.339116 | 0.163 | 0.038 | 1 |
| PCGF3 | 0.023186 | 0.339028 | 0.109 | 0.05 | 1 |
| ATP5G1 | 1.27E-05 | 0.338849 | 0.312 | 0.132 | 0.214925 |
| PDS5A | 0.000461 | 0.338764 | 0.178 | 0.063 | 1 |
| EIF4B | 1.38E-05 | 0.338712 | 0.222 | 0.069 | 0.234623 |
| RNF114 | 0.000406 | 0.338647 | 0.198 | 0.075 | 1 |
| ZFPL1 | 0.00058 | 0.338255 | 0.114 | 0.025 | 1 |
| CLK2 | 9.16E-05 | 0.338002 | 0.168 | 0.044 | 1 |
| HADHB | 0.000405 | 0.337811 | 0.211 | 0.088 | 1 |
| DBNL | 0.001487 | 0.337542 | 0.123 | 0.038 | 1 |
| SCARA3 | 6.60E-07 | 0.337315 | 0.275 | 0.082 | 0.011205 |
| ARFGAP1 | 0.001278 | 0.336045 | 0.157 | 0.057 | 1 |
| SNHG25 | 0.000866 | 0.336007 | 0.191 | 0.082 | 1 |
| CAP1 | 0.000213 | 0.335789 | 0.258 | 0.119 | 1 |
| ZC3H15 | 0.000318 | 0.335607 | 0.199 | 0.075 | 1 |
| PLAT | 0.012521 | 0.335563 | 0.177 | 0.094 | 1 |
| CEBPD | 1.08E-05 | 0.335359 | 0.567 | 0.358 | 0.184007 |
| CCDC6 | 0.000198 | 0.334646 | 0.167 | 0.05 | 1 |
| PFDN2 | 6.92E-05 | 0.33445 | 0.289 | 0.126 | 1 |
| TSTA3 | 4.16E-05 | 0.33442 | 0.254 | 0.101 | 0.706825 |
| DCAF7 | 0.000139 | 0.334387 | 0.17 | 0.05 | 1 |
| PTRHD1 | 0.000139 | 0.334214 | 0.159 | 0.044 | 1 |
| HIBADH | 0.001314 | 0.334154 | 0.107 | 0.025 | 1 |
| SRSF8 | 4.04E-05 | 0.333883 | 0.173 | 0.044 | 0.685694 |
| MRPS26 | 0.000577 | 0.333315 | 0.267 | 0.132 | 1 |
| KCTD12 | 0.001601 | 0.333009 | 0.228 | 0.113 | 1 |
| GCA | 0.001401 | 0.332946 | 0.116 | 0.031 | 1 |
| TNFSF10 | 5.44E-05 | 0.332734 | 0.189 | 0.057 | 0.924841 |
| SULF1 | 0.000661 | 0.332719 | 0.105 | 0.019 | 1 |
| COPS6 | 0.000471 | 0.33256 | 0.244 | 0.113 | 1 |
| SDHB | 0.000113 | 0.332138 | 0.258 | 0.107 | 1 |
| CXCL3 | 0.018734 | 0.332089 | 0.119 | 0.057 | 1 |
| TMPO | 0.007728 | 0.331628 | 0.132 | 0.057 | 1 |
| EMC7 | 0.040524 | 0.331218 | 0.101 | 0.05 | 1 |
| NXT1 | 0.000257 | 0.329971 | 0.136 | 0.031 | 1 |
| PLD3 | 0.000112 | 0.329566 | 0.232 | 0.088 | 1 |
| SPOP | 0.003386 | 0.32938 | 0.117 | 0.038 | 1 |
| HMGCS1 | 0.001536 | 0.327819 | 0.152 | 0.057 | 1 |
| TSEN54 | 0.000289 | 0.327784 | 0.114 | 0.019 | 1 |
| SNHG17 | 0.000176 | 0.327681 | 0.148 | 0.038 | 1 |
| TBRG4 | 0.000218 | 0.327265 | 0.126 | 0.025 | 1 |
| MTFR1L | 9.15E-05 | 0.3272 | 0.136 | 0.025 | 1 |
| POLR2H | 2.48E-05 | 0.327181 | 0.364 | 0.176 | 0.420476 |
| TIMM17A | 4.98E-05 | 0.327095 | 0.201 | 0.063 | 0.845555 |
| KDSR | 0.001642 | 0.327056 | 0.198 | 0.088 | 1 |
| NHP2L1 | 1.04E-06 | 0.327056 | 0.364 | 0.151 | 0.017662 |
| SCAF11 | 7.49E-05 | 0.32642 | 0.219 | 0.075 | 1 |
| GALNT12 | 0.000187 | 0.326064 | 0.107 | 0.013 | 1 |
| SUCLG1 | 0.000368 | 0.325925 | 0.159 | 0.05 | 1 |
| PPP1R15B | 0.002608 | 0.325497 | 0.199 | 0.094 | 1 |
| EIF2D | 0.000827 | 0.325381 | 0.12 | 0.031 | 1 |
| FOSL2 | 0.000488 | 0.324138 | 0.138 | 0.038 | 1 |
| CUEDC2 | 8.92E-05 | 0.323653 | 0.177 | 0.05 | 1 |
| ALDH2 | 3.02E-05 | 0.323575 | 0.148 | 0.025 | 0.513342 |
| ITGAV | 0.003872 | 0.323485 | 0.204 | 0.101 | 1 |
| VMP1 | 7.45E-06 | 0.323281 | 0.428 | 0.226 | 0.12661 |
| SYF2 | 3.20E-05 | 0.323213 | 0.212 | 0.069 | 0.542884 |
| DHTKD1 | 0.000291 | 0.323093 | 0.101 | 0.013 | 1 |
| ARPC2 | 0.000306 | 0.32243 | 0.422 | 0.245 | 1 |
| ARFGEF1 | 0.00143 | 0.322182 | 0.144 | 0.05 | 1 |
| CXCL16 | 0.000323 | 0.322055 | 0.189 | 0.069 | 1 |
| FEM1C | 0.02741 | 0.321642 | 0.106 | 0.05 | 1 |
| IRF2BP2 | 4.22E-06 | 0.32138 | 0.43 | 0.208 | 0.071609 |
| SYNE4 | 0.000501 | 0.321334 | 0.127 | 0.031 | 1 |
| CTSH | 0.02008 | 0.321114 | 0.165 | 0.094 | 1 |
| DNAJC15 | 0.001321 | 0.320942 | 0.117 | 0.031 | 1 |
| DCAF8 | 0.00084 | 0.320874 | 0.199 | 0.082 | 1 |
| CD44 | 0.000127 | 0.320598 | 0.122 | 0.019 | 1 |
| TBC1D15 | 0.000319 | 0.320552 | 0.111 | 0.019 | 1 |
| ALDOA | 4.36E-05 | 0.320216 | 0.705 | 0.516 | 0.741424 |
| SPATA20 | 0.001237 | 0.319645 | 0.117 | 0.031 | 1 |
| HDAC7 | 8.04E-07 | 0.319517 | 0.311 | 0.113 | 0.013655 |
| NDUFA6 | 9.79E-05 | 0.319242 | 0.193 | 0.063 | 1 |
| PIK3R3 | 0.000792 | 0.319109 | 0.101 | 0.019 | 1 |
| ARF1 | 1.43E-05 | 0.319007 | 0.514 | 0.296 | 0.243292 |
| ITM2C | 1.83E-07 | 0.318979 | 0.381 | 0.151 | 0.003109 |
| SMAD7 | 0.002761 | 0.318899 | 0.109 | 0.031 | 1 |
| DUS1L | 8.59E-05 | 0.318852 | 0.259 | 0.107 | 1 |
| FAM173A | 0.000199 | 0.318836 | 0.106 | 0.013 | 1 |
| HSD17B10 | 3.35E-05 | 0.318429 | 0.184 | 0.05 | 0.569811 |
| NDUFS4 | 2.87E-05 | 0.318228 | 0.209 | 0.063 | 0.488202 |
| FNBP4 | 0.000651 | 0.31808 | 0.24 | 0.113 | 1 |
| CALU | 6.70E-05 | 0.318066 | 0.232 | 0.088 | 1 |
| ANKIB1 | 0.001403 | 0.31782 | 0.106 | 0.025 | 1 |
| COA4 | 1.79E-05 | 0.317359 | 0.269 | 0.101 | 0.304041 |
| ZNF91 | 0.000586 | 0.317291 | 0.126 | 0.031 | 1 |
| MAP2K2 | 6.09E-05 | 0.316949 | 0.227 | 0.082 | 1 |
| TSPAN4 | 1.13E-05 | 0.316925 | 0.135 | 0.013 | 0.192402 |
| CCDC85B | 0.000509 | 0.316643 | 0.198 | 0.082 | 1 |
| CHTOP | 0.000199 | 0.315638 | 0.256 | 0.119 | 1 |
| SLPI | 0.001515 | 0.31556 | 0.694 | 0.528 | 1 |
| TFRC | 0.000304 | 0.315341 | 0.293 | 0.151 | 1 |
| PMEPA1 | 0.001155 | 0.314839 | 0.119 | 0.031 | 1 |
| SLC9A1 | 0.000193 | 0.314532 | 0.106 | 0.013 | 1 |
| CHCHD1 | 7.56E-05 | 0.314492 | 0.241 | 0.094 | 1 |
| RAB25 | 5.21E-05 | 0.313991 | 0.201 | 0.063 | 0.885043 |
| ITGB8 | 0.000486 | 0.313896 | 0.391 | 0.233 | 1 |
| NACAP1 | 9.78E-07 | 0.313725 | 0.241 | 0.063 | 0.016613 |
| PSMF1 | 0.000217 | 0.313644 | 0.194 | 0.069 | 1 |
| PMPCA | 0.003835 | 0.313614 | 0.115 | 0.038 | 1 |
| COX4I1 | 4.26E-07 | 0.313465 | 0.646 | 0.415 | 0.00724 |
| MTG1 | 0.000435 | 0.313402 | 0.109 | 0.019 | 1 |
| NOL4L | 0.003283 | 0.313241 | 0.126 | 0.044 | 1 |
| ATP5G2 | 5.09E-10 | 0.313181 | 0.643 | 0.327 | 8.65E-06 |
| KAT6A | 0.001161 | 0.312795 | 0.157 | 0.057 | 1 |
| SLC39A8 | 6.85E-05 | 0.312751 | 0.138 | 0.025 | 1 |
| MLF2 | 1.50E-06 | 0.312644 | 0.378 | 0.164 | 0.025401 |
| MAP3K1 | 0.005156 | 0.312457 | 0.13 | 0.05 | 1 |
| PDXDC1 | 0.00105 | 0.31235 | 0.167 | 0.063 | 1 |
| TRNP1 | 8.54E-05 | 0.312007 | 0.114 | 0.013 | 1 |
| SAR1A | 0.000168 | 0.311794 | 0.21 | 0.075 | 1 |
| FBXW5 | 0.000186 | 0.311637 | 0.201 | 0.075 | 1 |
| STK17B | 4.06E-05 | 0.310925 | 0.133 | 0.019 | 0.689646 |
| RAB13 | 0.000583 | 0.310777 | 0.189 | 0.075 | 1 |
| EFNB1 | 0.000209 | 0.31076 | 0.127 | 0.025 | 1 |
| SOX17 | 0.000764 | 0.310735 | 0.41 | 0.245 | 1 |
| KIAA0368 | 0.001886 | 0.310723 | 0.133 | 0.044 | 1 |
| MYOF | 0.000987 | 0.310437 | 0.204 | 0.088 | 1 |
| FDPS | 4.89E-05 | 0.309842 | 0.289 | 0.119 | 0.829931 |
| NOTCH3 | 7.84E-05 | 0.30979 | 0.159 | 0.038 | 1 |
| ARPC1A | 0.000131 | 0.309554 | 0.175 | 0.05 | 1 |
| HLA.DRB5 | 0.064509 | 0.309431 | 0.165 | 0.107 | 1 |
| EPOR | 0.00045 | 0.308793 | 0.128 | 0.031 | 1 |
| SNRPD3 | 3.19E-05 | 0.308506 | 0.278 | 0.113 | 0.54266 |
| ZDHHC12 | 0.000504 | 0.308288 | 0.105 | 0.019 | 1 |
| SREK1IP1 | 0.032439 | 0.308043 | 0.121 | 0.063 | 1 |
| TPP1 | 0.004866 | 0.307778 | 0.127 | 0.05 | 1 |
| ORMDL3 | 6.22E-05 | 0.307354 | 0.127 | 0.019 | 1 |
| ABCE1 | 5.78E-05 | 0.30729 | 0.141 | 0.025 | 0.981853 |
| C10orf11 | 0.001453 | 0.307105 | 0.114 | 0.031 | 1 |
| GLOD4 | 0.001251 | 0.306739 | 0.107 | 0.025 | 1 |
| HNRNPA1 | 8.74E-05 | 0.306692 | 0.389 | 0.214 | 1 |
| EGLN1 | 0.0049 | 0.306642 | 0.112 | 0.038 | 1 |
| ERGIC1 | 3.17E-05 | 0.306383 | 0.232 | 0.082 | 0.538298 |
| CANT1 | 0.000781 | 0.306301 | 0.112 | 0.025 | 1 |
| AGPAT6 | 0.017194 | 0.305749 | 0.154 | 0.082 | 1 |
| DPM3 | 3.01E-06 | 0.305722 | 0.294 | 0.101 | 0.051073 |
| SDR39U1 | 0.000451 | 0.305583 | 0.167 | 0.057 | 1 |
| LRPPRC | 0.000855 | 0.30558 | 0.195 | 0.082 | 1 |
| GSTO2 | 0.000381 | 0.305272 | 0.12 | 0.025 | 1 |
| DNAJC19 | 0.002094 | 0.30522 | 0.181 | 0.082 | 1 |
| RRP7A | 0.000872 | 0.305173 | 0.132 | 0.038 | 1 |
| BCKDHA | 0.001172 | 0.304599 | 0.137 | 0.044 | 1 |
| ATP1B3 | 0.000545 | 0.304547 | 0.216 | 0.094 | 1 |
| C8orf82 | 1.83E-06 | 0.304493 | 0.142 | 0.006 | 0.031081 |
| CMTM7 | 2.59E-05 | 0.30379 | 0.268 | 0.107 | 0.440711 |
| EIF4A2 | 9.31E-07 | 0.303767 | 0.763 | 0.509 | 0.01582 |
| NBN | 0.010041 | 0.303743 | 0.13 | 0.057 | 1 |
| IGSF9 | 0.000812 | 0.303295 | 0.111 | 0.025 | 1 |
| ABCA1 | 0.009103 | 0.302945 | 0.104 | 0.038 | 1 |
| MAF1 | 1.07E-05 | 0.302707 | 0.328 | 0.145 | 0.181165 |
| XPO6 | 0.000882 | 0.302439 | 0.142 | 0.044 | 1 |
| IDH3B | 0.002242 | 0.302293 | 0.163 | 0.069 | 1 |
| ZNF32 | 0.000453 | 0.302246 | 0.107 | 0.019 | 1 |
| NUP62 | 0.000196 | 0.301441 | 0.186 | 0.063 | 1 |
| KDELR1 | 2.54E-07 | 0.301402 | 0.299 | 0.088 | 0.004314 |
| NACC1 | 0.000126 | 0.300986 | 0.11 | 0.013 | 1 |
| CANX | 0.000265 | 0.300599 | 0.405 | 0.239 | 1 |
| ARF6 | 0.000154 | 0.300015 | 0.162 | 0.044 | 1 |
| CAPN1 | 8.13E-05 | 0.299789 | 0.335 | 0.17 | 1 |
| TRIB2 | 0.015691 | 0.299709 | 0.115 | 0.05 | 1 |
| SARS | 4.44E-05 | 0.299662 | 0.184 | 0.05 | 0.754455 |
| DSC2 | 0.006208 | 0.299575 | 0.116 | 0.044 | 1 |
| CPSF7 | 0.000554 | 0.299542 | 0.117 | 0.025 | 1 |
| PFKL | 9.89E-05 | 0.299254 | 0.293 | 0.138 | 1 |
| PRMT1 | 6.82E-05 | 0.299053 | 0.138 | 0.025 | 1 |
| SHROOM1 | 0.000164 | 0.299043 | 0.119 | 0.019 | 1 |
| VIM | 0.275947 | 0.298981 | 0.283 | 0.245 | 1 |
| FAM46C | 0.012066 | 0.298784 | 0.173 | 0.088 | 1 |
| SNHG7 | 1.36E-05 | 0.29863 | 0.362 | 0.164 | 0.231042 |
| IFNAR1 | 0.004476 | 0.298435 | 0.141 | 0.057 | 1 |
| UQCR10 | 1.36E-07 | 0.298412 | 0.352 | 0.126 | 0.00231 |
| MTHFD2 | 0.000128 | 0.298186 | 0.193 | 0.063 | 1 |
| HELZ | 0.000993 | 0.297811 | 0.168 | 0.063 | 1 |
| PPFIA1 | 0.000427 | 0.297801 | 0.12 | 0.025 | 1 |
| SNX5 | 2.26E-05 | 0.297606 | 0.251 | 0.088 | 0.384023 |
| NSUN5P2 | 0.000812 | 0.297527 | 0.112 | 0.025 | 1 |
| PPIA | 3.37E-06 | 0.297369 | 0.44 | 0.233 | 0.057197 |
| HDDC2 | 1.90E-05 | 0.297302 | 0.152 | 0.025 | 0.322344 |
| SMARCE1 | 0.000524 | 0.29723 | 0.195 | 0.075 | 1 |
| MRPS12 | 3.00E-05 | 0.297148 | 0.199 | 0.057 | 0.508979 |
| ANP32E | 0.033909 | 0.296993 | 0.146 | 0.082 | 1 |
| RAB21 | 0.004141 | 0.296871 | 0.121 | 0.044 | 1 |
| FGFR2 | 9.56E-05 | 0.296823 | 0.157 | 0.038 | 1 |
| AIP | 0.005648 | 0.296467 | 0.128 | 0.05 | 1 |
| ERBB3 | 0.000276 | 0.296377 | 0.235 | 0.101 | 1 |
| EPB41L4A.AS1 | 4.39E-05 | 0.296119 | 0.235 | 0.082 | 0.744943 |
| NDUFA1 | 3.79E-07 | 0.295992 | 0.39 | 0.164 | 0.006442 |
| RFK | 0.005058 | 0.295949 | 0.137 | 0.057 | 1 |
| CARHSP1 | 6.67E-05 | 0.294769 | 0.198 | 0.063 | 1 |
| HDHD3 | 0.008244 | 0.294573 | 0.141 | 0.063 | 1 |
| PRSS21 | 6.04E-05 | 0.29425 | 0.105 | 0.006 | 1 |
| TMEM181 | 0.000763 | 0.294221 | 0.104 | 0.019 | 1 |
| RALBP1 | 0.001866 | 0.294061 | 0.194 | 0.088 | 1 |
| CCZ1 | 0.002659 | 0.293745 | 0.147 | 0.057 | 1 |
| UGCG | 0.001013 | 0.293716 | 0.251 | 0.119 | 1 |
| DDRGK1 | 0.003085 | 0.293129 | 0.199 | 0.094 | 1 |
| GRAMD1A | 0.000375 | 0.293044 | 0.179 | 0.063 | 1 |
| TMEM167A | 0.004732 | 0.292872 | 0.132 | 0.05 | 1 |
| LACTB2 | 0.003477 | 0.292454 | 0.178 | 0.082 | 1 |
| SLC7A5P2 | 0.004272 | 0.292453 | 0.104 | 0.031 | 1 |
| VEZT | 0.009982 | 0.292447 | 0.112 | 0.044 | 1 |
| SAE1 | 0.000686 | 0.292328 | 0.102 | 0.019 | 1 |
| UNC45A | 5.16E-05 | 0.292197 | 0.142 | 0.025 | 0.875923 |
| BRD4 | 0.000747 | 0.291822 | 0.178 | 0.069 | 1 |
| PRPF31 | 0.000883 | 0.291313 | 0.152 | 0.05 | 1 |
| MLLT10 | 0.007444 | 0.291221 | 0.125 | 0.05 | 1 |
| ADNP | 0.000193 | 0.291102 | 0.18 | 0.057 | 1 |
| EIF2A | 0.000205 | 0.290894 | 0.127 | 0.025 | 1 |
| B4GALT2 | 0.000207 | 0.290833 | 0.137 | 0.031 | 1 |
| REC8 | 0.000503 | 0.290775 | 0.302 | 0.157 | 1 |
| NPC2 | 1.64E-06 | 0.29076 | 0.452 | 0.226 | 0.027868 |
| PRDX6 | 1.27E-05 | 0.290587 | 0.595 | 0.358 | 0.215901 |
| LGALS8 | 0.007542 | 0.290494 | 0.159 | 0.075 | 1 |
| SRRM1 | 0.001065 | 0.290433 | 0.183 | 0.075 | 1 |
| ATP5J2 | 0.000126 | 0.290161 | 0.341 | 0.176 | 1 |
| GTF2H3 | 0.007543 | 0.290106 | 0.115 | 0.044 | 1 |
| MRPL55 | 0.000166 | 0.289972 | 0.189 | 0.063 | 1 |
| INSIG1 | 0.018855 | 0.289696 | 0.105 | 0.044 | 1 |
| RBM33 | 0.005178 | 0.289659 | 0.12 | 0.044 | 1 |
| TARBP1 | 0.021827 | 0.2889 | 0.156 | 0.082 | 1 |
| SLC35F6 | 4.71E-05 | 0.288881 | 0.152 | 0.031 | 0.799795 |
| BRD9 | 0.000648 | 0.288534 | 0.136 | 0.038 | 1 |
| CGN | 0.00606 | 0.288234 | 0.186 | 0.094 | 1 |
| GNPTG | 5.98E-05 | 0.288229 | 0.17 | 0.044 | 1 |
| LAGE3 | 0.000483 | 0.288178 | 0.127 | 0.031 | 1 |
| NDUFV3 | 0.008401 | 0.287576 | 0.141 | 0.063 | 1 |
| DERL1 | 0.000939 | 0.287232 | 0.228 | 0.107 | 1 |
| OLA1 | 1.44E-05 | 0.287039 | 0.175 | 0.038 | 0.244458 |
| TNKS | 0.011742 | 0.286995 | 0.101 | 0.038 | 1 |
| LSM14B | 0.00047 | 0.28687 | 0.13 | 0.031 | 1 |
| POLG2 | 0.000995 | 0.286738 | 0.132 | 0.038 | 1 |
| IFI30 | 0.002081 | 0.286647 | 0.101 | 0.025 | 1 |
| KANSL2 | 0.021028 | 0.286643 | 0.136 | 0.069 | 1 |
| COX8A | 1.99E-07 | 0.28653 | 0.62 | 0.358 | 0.003376 |
| DRAP1 | 0.00033 | 0.286212 | 0.204 | 0.082 | 1 |
| TSPO | 2.31E-08 | 0.286048 | 0.46 | 0.208 | 0.000393 |
| AVPI1 | 0.001492 | 0.285955 | 0.106 | 0.025 | 1 |
| MT1F | 0.000153 | 0.285912 | 0.121 | 0.019 | 1 |
| SETDB1 | 0.013942 | 0.285884 | 0.125 | 0.057 | 1 |
| TEX264 | 0.009129 | 0.285875 | 0.198 | 0.107 | 1 |
| RBM7 | 0.012514 | 0.285812 | 0.109 | 0.044 | 1 |
| NFYC | 0.027981 | 0.285408 | 0.117 | 0.057 | 1 |
| NOB1 | 0.000957 | 0.285201 | 0.151 | 0.05 | 1 |
| BTBD2 | 6.38E-05 | 0.284622 | 0.105 | 0.006 | 1 |
| TFE3 | 0.005524 | 0.284506 | 0.109 | 0.038 | 1 |
| ARID5B | 0.005663 | 0.283938 | 0.119 | 0.044 | 1 |
| SLC9A3R1 | 0.000475 | 0.283743 | 0.367 | 0.22 | 1 |
| OBSCN | 0.002122 | 0.283731 | 0.142 | 0.05 | 1 |
| DNASE2 | 0.002049 | 0.283647 | 0.191 | 0.088 | 1 |
| PAPD4 | 0.010661 | 0.283443 | 0.102 | 0.038 | 1 |
| CDV3 | 6.43E-05 | 0.283135 | 0.237 | 0.088 | 1 |
| RNF181 | 1.42E-07 | 0.28246 | 0.295 | 0.088 | 0.002418 |
| COX20 | 0.000222 | 0.280992 | 0.207 | 0.082 | 1 |
| SLC7A2 | 0.000956 | 0.280768 | 0.196 | 0.082 | 1 |
| JTB | 0.000135 | 0.280724 | 0.415 | 0.233 | 1 |
| NDUFS5 | 4.27E-06 | 0.280669 | 0.438 | 0.22 | 0.072555 |
| C19orf24 | 0.000966 | 0.28017 | 0.151 | 0.05 | 1 |
| RPS2P32 | 0.000159 | 0.280074 | 0.157 | 0.044 | 1 |
| HP1BP3 | 0.000488 | 0.279962 | 0.301 | 0.151 | 1 |
| FBXW4 | 0.005509 | 0.279182 | 0.156 | 0.069 | 1 |
| MTA1 | 4.39E-05 | 0.279167 | 0.243 | 0.088 | 0.745465 |
| LAMA5 | 3.46E-07 | 0.279122 | 0.452 | 0.208 | 0.005881 |
| SH3GLB2 | 0.000829 | 0.278586 | 0.23 | 0.107 | 1 |
| PA2G4 | 8.28E-05 | 0.278161 | 0.257 | 0.107 | 1 |
| OVOL1 | 0.080144 | 0.278073 | 0.14 | 0.088 | 1 |
| NCOA1 | 0.001355 | 0.277733 | 0.127 | 0.038 | 1 |
| MCCC1 | 0.005554 | 0.277573 | 0.119 | 0.044 | 1 |
| MRPL24 | 0.000307 | 0.277452 | 0.223 | 0.094 | 1 |
| SRSF9 | 2.41E-05 | 0.277158 | 0.359 | 0.17 | 0.408955 |
| IPO9 | 0.002084 | 0.277103 | 0.13 | 0.044 | 1 |
| MYH9 | 1.07E-05 | 0.276944 | 0.344 | 0.157 | 0.181251 |
| PCNP | 0.000312 | 0.276554 | 0.302 | 0.151 | 1 |
| RLIM | 0.000773 | 0.276486 | 0.202 | 0.088 | 1 |
| SGMS1 | 0.000382 | 0.276443 | 0.122 | 0.025 | 1 |
| EMX2 | 0.019836 | 0.276416 | 0.167 | 0.094 | 1 |
| TIMM8B | 0.000106 | 0.276135 | 0.217 | 0.082 | 1 |
| EFEMP2 | 0.000489 | 0.27598 | 0.138 | 0.038 | 1 |
| CRYAB | 4.51E-05 | 0.275964 | 0.285 | 0.119 | 0.766067 |
| NDUFB2 | 8.77E-06 | 0.27591 | 0.443 | 0.233 | 0.148911 |
| RAI1 | 0.002555 | 0.275843 | 0.168 | 0.075 | 1 |
| APPL1 | 0.000746 | 0.275625 | 0.112 | 0.025 | 1 |
| CREG1 | 2.10E-06 | 0.27557 | 0.281 | 0.094 | 0.035592 |
| CBS | 7.93E-05 | 0.275553 | 0.179 | 0.05 | 1 |
| IMPAD1 | 0.005174 | 0.275533 | 0.181 | 0.088 | 1 |
| PTK2 | 0.009696 | 0.275251 | 0.157 | 0.075 | 1 |
| MTDH | 4.29E-06 | 0.274935 | 0.446 | 0.226 | 0.072952 |
| SMG7 | 0.003589 | 0.274912 | 0.117 | 0.038 | 1 |
| ACAA1 | 0.000179 | 0.274811 | 0.185 | 0.063 | 1 |
| BRAT1 | 0.002934 | 0.274719 | 0.11 | 0.031 | 1 |
| CEBPZ | 0.000624 | 0.27456 | 0.105 | 0.019 | 1 |
| PBX2 | 0.044128 | 0.274478 | 0.109 | 0.057 | 1 |
| HIGD1A | 0.001052 | 0.273707 | 0.237 | 0.113 | 1 |
| GBA2 | 0.000568 | 0.273596 | 0.117 | 0.025 | 1 |
| DECR1 | 0.000597 | 0.273211 | 0.164 | 0.057 | 1 |
| GPC1 | 0.001997 | 0.273031 | 0.132 | 0.044 | 1 |
| IDI1 | 0.001018 | 0.272507 | 0.151 | 0.05 | 1 |
| ZDHHC4 | 0.004793 | 0.272218 | 0.121 | 0.044 | 1 |
| AFF1 | 0.005609 | 0.271646 | 0.128 | 0.05 | 1 |
| TBC1D5 | 0.013775 | 0.270811 | 0.109 | 0.044 | 1 |
| PNISR | 0.007595 | 0.270248 | 0.231 | 0.132 | 1 |
| PDAP1 | 0.00013 | 0.270095 | 0.26 | 0.113 | 1 |
| SLC39A1 | 0.000428 | 0.270055 | 0.184 | 0.069 | 1 |
| WHSC1 | 0.006711 | 0.270007 | 0.107 | 0.038 | 1 |
| NDUFA5 | 0.005863 | 0.269965 | 0.253 | 0.145 | 1 |
| ACTN1 | 0.000633 | 0.269828 | 0.295 | 0.157 | 1 |
| NOC2L | 0.003554 | 0.269713 | 0.16 | 0.069 | 1 |
| SPRY2 | 0.031451 | 0.269613 | 0.106 | 0.05 | 1 |
| EFTUD2 | 0.000482 | 0.269516 | 0.189 | 0.069 | 1 |
| UBR5 | 0.000265 | 0.269414 | 0.205 | 0.075 | 1 |
| CTDNEP1 | 0.000534 | 0.269321 | 0.205 | 0.082 | 1 |
| ZYG11B | 0.001725 | 0.268963 | 0.115 | 0.031 | 1 |
| PLEKHG3 | 0.004727 | 0.268944 | 0.102 | 0.031 | 1 |
| COPS2 | 0.000568 | 0.268825 | 0.146 | 0.044 | 1 |
| EPAS1 | 0.012121 | 0.268711 | 0.101 | 0.038 | 1 |
| RIPK2 | 0.006171 | 0.268593 | 0.136 | 0.057 | 1 |
| SRPK1 | 0.002496 | 0.268255 | 0.117 | 0.038 | 1 |
| PGRMC2 | 0.002314 | 0.268057 | 0.201 | 0.094 | 1 |
| NPAS2 | 0.002065 | 0.268004 | 0.16 | 0.063 | 1 |
| ACSF2 | 0.012132 | 0.267809 | 0.101 | 0.038 | 1 |
| SDHD | 0.000419 | 0.266943 | 0.178 | 0.063 | 1 |
| TMEM263 | 0.003002 | 0.266939 | 0.107 | 0.031 | 1 |
| CYP51A1 | 0.000175 | 0.266775 | 0.128 | 0.025 | 1 |
| C1orf198 | 0.000175 | 0.266519 | 0.13 | 0.025 | 1 |
| RABAC1 | 0.004149 | 0.266331 | 0.254 | 0.138 | 1 |
| EHMT2 | 0.001686 | 0.266056 | 0.143 | 0.05 | 1 |
| COMTD1 | 0.001825 | 0.26599 | 0.178 | 0.075 | 1 |
| CCDC14 | 0.000986 | 0.265816 | 0.151 | 0.05 | 1 |
| NDUFS6 | 0.000644 | 0.265752 | 0.388 | 0.245 | 1 |
| EMC6 | 0.002945 | 0.26553 | 0.16 | 0.069 | 1 |
| NDUFA13 | 7.34E-06 | 0.2655 | 0.53 | 0.308 | 0.124605 |
| AEBP2 | 0.014212 | 0.265491 | 0.142 | 0.069 | 1 |
| SGK1 | 0.005515 | 0.265405 | 0.242 | 0.138 | 1 |
| IRF3 | 0.03772 | 0.265363 | 0.142 | 0.082 | 1 |
| MRPL14 | 0.002919 | 0.26533 | 0.223 | 0.113 | 1 |
| MCCC2 | 0.003428 | 0.265256 | 0.153 | 0.063 | 1 |
| CHD1L | 0.037487 | 0.265155 | 0.102 | 0.05 | 1 |
| TMEM134 | 2.96E-05 | 0.264967 | 0.211 | 0.069 | 0.502183 |
| BRK1 | 2.62E-05 | 0.264873 | 0.348 | 0.17 | 0.445411 |
| TNS3 | 0.003417 | 0.264772 | 0.125 | 0.044 | 1 |
| TATDN2 | 0.000204 | 0.264637 | 0.105 | 0.013 | 1 |
| AHDC1 | 0.006454 | 0.264302 | 0.126 | 0.05 | 1 |
| VKORC1L1 | 0.002905 | 0.26422 | 0.117 | 0.038 | 1 |
| SUCO | 0.018066 | 0.264192 | 0.114 | 0.05 | 1 |
| ATF2 | 0.004672 | 0.263857 | 0.102 | 0.031 | 1 |
| ILVBL | 0.000336 | 0.263738 | 0.18 | 0.063 | 1 |
| C15orf39 | 0.002349 | 0.263715 | 0.11 | 0.031 | 1 |
| KLF10 | 0.001845 | 0.263686 | 0.409 | 0.277 | 1 |
| SNHG1 | 3.70E-07 | 0.263625 | 0.486 | 0.233 | 0.006285 |
| B4GALT5 | 0.000762 | 0.263516 | 0.183 | 0.069 | 1 |
| ZNF384 | 0.00476 | 0.263119 | 0.137 | 0.057 | 1 |
| RUSC1 | 0.00092 | 0.263108 | 0.13 | 0.038 | 1 |
| EIF3F | 3.00E-05 | 0.262743 | 0.191 | 0.05 | 0.510266 |
| PSME2 | 2.87E-05 | 0.262525 | 0.265 | 0.101 | 0.487722 |
| FPGS | 0.000314 | 0.262464 | 0.17 | 0.057 | 1 |
| HIF1A | 1.19E-05 | 0.262201 | 0.267 | 0.094 | 0.202877 |
| ASAH1 | 0.000825 | 0.262117 | 0.198 | 0.082 | 1 |
| TUBA4A | 0.004327 | 0.26208 | 0.13 | 0.05 | 1 |
| ANP32B | 9.50E-08 | 0.262068 | 0.362 | 0.132 | 0.001613 |
| MRPL51 | 0.002195 | 0.262005 | 0.32 | 0.189 | 1 |
| ATXN8OS | 1.99E-05 | 0.261966 | 0.127 | 0.013 | 0.337789 |
| GNL3 | 0.03296 | 0.26166 | 0.114 | 0.057 | 1 |
| CLCF1 | 0.000129 | 0.26138 | 0.142 | 0.031 | 1 |
| PFN1 | 1.41E-07 | 0.261321 | 0.675 | 0.447 | 0.00239 |
| NT5C2 | 0.00029 | 0.26111 | 0.22 | 0.088 | 1 |
| CLEC2D | 0.000267 | 0.261068 | 0.104 | 0.013 | 1 |
| SLC35B1 | 0.002421 | 0.261054 | 0.147 | 0.057 | 1 |
| PRSS23 | 0.000496 | 0.261038 | 0.137 | 0.038 | 1 |
| SNRPB | 3.77E-07 | 0.260942 | 0.444 | 0.208 | 0.006401 |
| ZRANB2 | 0.001636 | 0.260177 | 0.251 | 0.132 | 1 |
| NXN | 0.010993 | 0.260044 | 0.12 | 0.05 | 1 |
| GSS | 0.003057 | 0.259699 | 0.115 | 0.038 | 1 |
| SNHG16 | 0.000143 | 0.259614 | 0.368 | 0.195 | 1 |
| ANAPC7 | 0.087223 | 0.259597 | 0.123 | 0.075 | 1 |
| CDK10 | 0.006654 | 0.259321 | 0.126 | 0.05 | 1 |
| ATP11A | 0.001714 | 0.25907 | 0.105 | 0.025 | 1 |
| ATXN10 | 0.051331 | 0.258965 | 0.157 | 0.094 | 1 |
| RPS6KB2 | 0.003467 | 0.258935 | 0.115 | 0.038 | 1 |
| ISOC2 | 7.12E-05 | 0.258752 | 0.115 | 0.013 | 1 |
| MRPL34 | 0.000104 | 0.258594 | 0.205 | 0.069 | 1 |
| TLK1 | 0.002092 | 0.25846 | 0.112 | 0.031 | 1 |
| TKFC | 0.001652 | 0.258341 | 0.104 | 0.025 | 1 |
| SUPT4H1 | 0.000112 | 0.258203 | 0.201 | 0.069 | 1 |
| SLC39A6 | 0.001166 | 0.257707 | 0.117 | 0.031 | 1 |
| HSPA14 | 0.000632 | 0.257658 | 0.126 | 0.031 | 1 |
| SKIL | 0.018407 | 0.257316 | 0.236 | 0.145 | 1 |
| YWHAZ | 0.000126 | 0.256543 | 0.458 | 0.258 | 1 |
| LAMP2 | 0.019173 | 0.25654 | 0.26 | 0.164 | 1 |
| SLC29A2 | 0.000101 | 0.256494 | 0.112 | 0.013 | 1 |
| C14orf166 | 5.90E-05 | 0.256482 | 0.263 | 0.101 | 1 |
| SERBP1 | 1.85E-06 | 0.256478 | 0.367 | 0.151 | 0.03137 |
| TRIAP1 | 0.019232 | 0.256257 | 0.104 | 0.044 | 1 |
| ANXA4 | 0.001985 | 0.256226 | 0.247 | 0.126 | 1 |
| BACE2 | 0.000608 | 0.256124 | 0.247 | 0.113 | 1 |
| DCAF10 | 0.017003 | 0.255672 | 0.131 | 0.063 | 1 |
| ST14 | 0.008249 | 0.254604 | 0.298 | 0.182 | 1 |
| NAA25 | 0.018066 | 0.254231 | 0.114 | 0.05 | 1 |
| TROVE2 | 4.50E-05 | 0.254231 | 0.133 | 0.019 | 0.765196 |
| TCEAL4 | 9.78E-05 | 0.253707 | 0.211 | 0.075 | 1 |
| MREG | 0.001542 | 0.253673 | 0.105 | 0.025 | 1 |
| HERPUD2 | 0.009771 | 0.253636 | 0.105 | 0.038 | 1 |
| TNFRSF21 | 0.001058 | 0.253527 | 0.141 | 0.044 | 1 |
| MAGED1 | 8.36E-05 | 0.253183 | 0.254 | 0.101 | 1 |
| RFXANK | 0.005566 | 0.253072 | 0.206 | 0.107 | 1 |
| USP39 | 2.77E-05 | 0.253059 | 0.127 | 0.013 | 0.47073 |
| CNPPD1 | 6.79E-05 | 0.252731 | 0.141 | 0.025 | 1 |
| CLDND1 | 0.000449 | 0.252412 | 0.13 | 0.031 | 1 |
| RAB1A | 0.002832 | 0.25234 | 0.351 | 0.22 | 1 |
| HSPD1 | 0.001606 | 0.252254 | 0.56 | 0.403 | 1 |
| KRT18 | 0.000203 | 0.251956 | 0.78 | 0.591 | 1 |
| HLA.DQB1 | 0.00253 | 0.251355 | 0.219 | 0.107 | 1 |
| VAMP2 | 0.001169 | 0.251048 | 0.167 | 0.063 | 1 |
| PLAGL2 | 0.049567 | 0.250969 | 0.115 | 0.063 | 1 |
| ECH1 | 0.001876 | 0.250898 | 0.206 | 0.094 | 1 |
| TMEM251 | 0.000144 | 0.25044 | 0.109 | 0.013 | 1 |
| SNX4 | 0.000866 | 0.25027 | 0.112 | 0.025 | 1 |
| CHKA | 0.000318 | 0.250237 | 0.123 | 0.025 | 1 |
| CCNL2 | 0.051926 | -0.250134 | 0.299 | 0.201 | 1 |
| RB1CC1 | 0.463607 | -0.25077 | 0.13 | 0.145 | 1 |
| MAZ | 0.400223 | -0.251371 | 0.206 | 0.164 | 1 |
| IFT80 | 0.256151 | -0.251538 | 0.077 | 0.101 | 1 |
| ANKRD11 | 0.278743 | -0.251827 | 0.116 | 0.082 | 1 |
| STX16 | 0.092107 | -0.252041 | 0.225 | 0.151 | 1 |
| GTF2B | 0.127891 | -0.252616 | 0.114 | 0.151 | 1 |
| RPN2 | 0.002644 | -0.252863 | 0.375 | 0.22 | 1 |
| NAP1L4 | 0.7322 | -0.252932 | 0.174 | 0.151 | 1 |
| NFIL3 | 0.396225 | -0.253076 | 0.14 | 0.107 | 1 |
| PNMA1 | 0.171162 | -0.253102 | 0.119 | 0.151 | 1 |
| UBE2N | 0.145668 | -0.253229 | 0.206 | 0.145 | 1 |
| FKBP1A | 0.239089 | -0.253325 | 0.314 | 0.239 | 1 |
| SORT1 | 0.063873 | -0.25349 | 0.167 | 0.101 | 1 |
| ZNF516 | 0.959502 | -0.254303 | 0.104 | 0.101 | 1 |
| CDC42SE2 | 0.243995 | -0.254717 | 0.112 | 0.075 | 1 |
| DCTN3 | 0.973492 | -0.255041 | 0.168 | 0.157 | 1 |
| PPP2R1A | 0.281941 | -0.255443 | 0.317 | 0.245 | 1 |
| MBD4 | 0.033923 | -0.255616 | 0.107 | 0.05 | 1 |
| AKAP17A | 0.006923 | -0.255773 | 0.148 | 0.063 | 1 |
| LAPTM4A | 0.052267 | -0.255897 | 0.437 | 0.314 | 1 |
| HEBP2 | 0.283025 | -0.255994 | 0.248 | 0.189 | 1 |
| SRPRB | 0.211189 | -0.25698 | 0.104 | 0.069 | 1 |
| C11orf54 | 0.194626 | -0.258036 | 0.116 | 0.075 | 1 |
| CAD | 0.001033 | -0.258105 | 0.112 | 0.025 | 1 |
| TM2D3 | 0.215117 | -0.258375 | 0.122 | 0.082 | 1 |
| SAP30BP | 0.983672 | -0.258389 | 0.138 | 0.132 | 1 |
| MARCKS | 0.967612 | -0.259937 | 0.173 | 0.164 | 1 |
| HLA.A | 0.236854 | -0.260189 | 0.646 | 0.497 | 1 |
| ZBED5 | 0.392313 | -0.260562 | 0.114 | 0.088 | 1 |
| LINC00493 | 0.374813 | -0.260733 | 0.252 | 0.201 | 1 |
| GBAS | 0.948595 | -0.260852 | 0.165 | 0.164 | 1 |
| MBP | 0.505254 | -0.260888 | 0.102 | 0.113 | 1 |
| C4orf48 | 0.53463 | -0.26091 | 0.247 | 0.258 | 1 |
| ALYREF | 0.022261 | -0.260931 | 0.142 | 0.069 | 1 |
| TAF7 | 0.78414 | -0.262018 | 0.233 | 0.201 | 1 |
| AKR7A2 | 0.888028 | -0.262644 | 0.125 | 0.113 | 1 |
| C18orf25 | 0.04364 | -0.263918 | 0.059 | 0.101 | 1 |
| PRUNE2 | 0.094434 | -0.264158 | 0.077 | 0.113 | 1 |
| CLK1 | 0.009249 | -0.264164 | 0.447 | 0.289 | 1 |
| SERPINB1 | 0.491929 | -0.264851 | 0.226 | 0.239 | 1 |
| FLOT2 | 0.49922 | -0.264862 | 0.104 | 0.082 | 1 |
| SLC35A2 | 0.160744 | -0.265378 | 0.109 | 0.145 | 1 |
| ING1 | 0.653408 | -0.26572 | 0.194 | 0.17 | 1 |
| BDH2 | 0.146665 | -0.265978 | 0.117 | 0.075 | 1 |
| MLLT4 | 0.608905 | -0.266052 | 0.368 | 0.314 | 1 |
| GPBP1 | 0.624789 | -0.266351 | 0.225 | 0.195 | 1 |
| RNF130 | 0.12214 | -0.266466 | 0.14 | 0.088 | 1 |
| PARP14 | 0.182684 | -0.268123 | 0.193 | 0.138 | 1 |
| PI4KB | 0.7623 | -0.26825 | 0.159 | 0.138 | 1 |
| CSTB | 0.655252 | -0.26856 | 0.556 | 0.491 | 1 |
| IFNGR1 | 0.25536 | -0.268714 | 0.115 | 0.082 | 1 |
| DCAF5 | 0.238003 | -0.269253 | 0.148 | 0.107 | 1 |
| LYPLA2 | 0.484255 | -0.269319 | 0.154 | 0.17 | 1 |
| TNFRSF14 | 0.043062 | -0.270224 | 0.169 | 0.101 | 1 |
| WDR45B | 0.995002 | -0.270624 | 0.311 | 0.283 | 1 |
| TECR | 0.233004 | -0.270793 | 0.114 | 0.075 | 1 |
| IPO4 | 0.609393 | -0.270818 | 0.111 | 0.094 | 1 |
| EIF4G3 | 0.972493 | -0.271673 | 0.105 | 0.101 | 1 |
| SEC24B | 0.185687 | -0.272597 | 0.115 | 0.075 | 1 |
| TOMM34 | 0.358889 | -0.272693 | 0.119 | 0.138 | 1 |
| NDUFAF3 | 0.859004 | -0.272724 | 0.212 | 0.201 | 1 |
| FKBP9 | 0.023814 | -0.273149 | 0.125 | 0.057 | 1 |
| TEX10 | 0.00296 | -0.27323 | 0.111 | 0.031 | 1 |
| SYPL1 | 0.465208 | -0.274102 | 0.315 | 0.258 | 1 |
| RBP1 | 0.633104 | -0.274797 | 0.409 | 0.384 | 1 |
| SEC16A | 0.640168 | -0.275598 | 0.133 | 0.113 | 1 |
| ST5 | 0.779243 | -0.275629 | 0.102 | 0.107 | 1 |
| GTF3C2 | 0.352008 | -0.275816 | 0.156 | 0.119 | 1 |
| NFX1 | 0.099336 | -0.275945 | 0.111 | 0.063 | 1 |
| RAB7A | 0.681385 | -0.276221 | 0.296 | 0.258 | 1 |
| YWHAE | 0.343041 | -0.276278 | 0.375 | 0.302 | 1 |
| TUSC2 | 0.785158 | -0.276447 | 0.105 | 0.107 | 1 |
| BOC | 0.034744 | -0.276615 | 0.143 | 0.075 | 1 |
| GABARAPL2 | 0.115746 | -0.276904 | 0.328 | 0.233 | 1 |
| ISG20 | 0.795383 | -0.277237 | 0.112 | 0.113 | 1 |
| CLIC1 | 0.557937 | -0.277352 | 0.34 | 0.327 | 1 |
| SPTAN1 | 0.699349 | -0.277777 | 0.237 | 0.233 | 1 |
| ZFP36 | 0.142037 | -0.278505 | 0.746 | 0.698 | 1 |
| SUB1 | 0.514713 | -0.279719 | 0.322 | 0.264 | 1 |
| NEDD8 | 0.140114 | -0.27972 | 0.395 | 0.283 | 1 |
| LEMD2 | 0.070232 | -0.280011 | 0.154 | 0.094 | 1 |
| PPP1R10 | 0.370922 | -0.280469 | 0.101 | 0.075 | 1 |
| CNOT1 | 0.621126 | -0.280878 | 0.127 | 0.107 | 1 |
| TMEM123 | 0.648473 | -0.281267 | 0.299 | 0.258 | 1 |
| TMEM248 | 0.657244 | -0.281323 | 0.149 | 0.126 | 1 |
| FOXP1 | 0.78013 | -0.281461 | 0.207 | 0.201 | 1 |
| ZNF326 | 0.039209 | -0.281476 | 0.133 | 0.069 | 1 |
| ADGRL1 | 0.019634 | -0.281778 | 0.125 | 0.057 | 1 |
| VPS29 | 0.073614 | -0.282289 | 0.19 | 0.119 | 1 |
| GRHPR | 5.78E-05 | -0.284402 | 0.254 | 0.101 | 0.981391 |
| CUL5 | 0.326641 | -0.28443 | 0.102 | 0.075 | 1 |
| PSMD7 | 0.269954 | -0.284567 | 0.162 | 0.119 | 1 |
| SLK | 0.495197 | -0.2849 | 0.101 | 0.082 | 1 |
| MRPL41 | 0.330113 | -0.285165 | 0.251 | 0.195 | 1 |
| CTNNB1 | 0.318058 | -0.285684 | 0.335 | 0.264 | 1 |
| NUDCD3 | 0.31272 | -0.285922 | 0.114 | 0.082 | 1 |
| NEK7 | 0.364816 | -0.28634 | 0.135 | 0.101 | 1 |
| ILF3.AS1 | 0.063827 | -0.286564 | 0.109 | 0.057 | 1 |
| UBE2W | 0.054931 | -0.287402 | 0.101 | 0.05 | 1 |
| SYT8 | 0.014665 | -0.287584 | 0.052 | 0.101 | 1 |
| KIAA2013 | 0.08268 | -0.287614 | 0.121 | 0.069 | 1 |
| SPATA13 | 0.197173 | -0.288154 | 0.093 | 0.126 | 1 |
| HAGH | 0.396238 | -0.288992 | 0.102 | 0.119 | 1 |
| SPRYD3 | 0.021288 | -0.289012 | 0.059 | 0.107 | 1 |
| GLYR1 | 0.515952 | -0.289508 | 0.126 | 0.138 | 1 |
| BBS1 | 0.083797 | -0.289854 | 0.064 | 0.101 | 1 |
| ILF3 | 0.010159 | -0.290846 | 0.396 | 0.252 | 1 |
| MYCBP2 | 0.06021 | -0.290904 | 0.133 | 0.075 | 1 |
| SMARCA1 | 0.638914 | -0.29115 | 0.119 | 0.101 | 1 |
| WDR77 | 0.278388 | -0.291927 | 0.109 | 0.075 | 1 |
| MAN1B1 | 0.502476 | -0.292173 | 0.111 | 0.088 | 1 |
| NOP10 | 0.037634 | -0.292402 | 0.312 | 0.201 | 1 |
| LONP1 | 0.047659 | -0.292717 | 0.13 | 0.069 | 1 |
| SERP1 | 0.122096 | -0.293169 | 0.474 | 0.346 | 1 |
| MALAT1 | 0.004026 | -0.293527 | 0.8 | 0.786 | 1 |
| CCDC107 | 0.179865 | -0.294177 | 0.091 | 0.119 | 1 |
| GPR107 | 0.496844 | -0.29432 | 0.102 | 0.082 | 1 |
| ARRDC2 | 0.735781 | -0.295167 | 0.133 | 0.119 | 1 |
| SSNA1 | 0.127931 | -0.295261 | 0.184 | 0.126 | 1 |
| NFE2L1 | 0.782727 | -0.295605 | 0.179 | 0.176 | 1 |
| FAM102A | 0.22449 | -0.296257 | 0.141 | 0.17 | 1 |
| SMIM14 | 0.484149 | -0.296288 | 0.119 | 0.132 | 1 |
| SLC30A9 | 0.513296 | -0.296293 | 0.127 | 0.101 | 1 |
| ERBB4 | 0.066732 | -0.296844 | 0.078 | 0.119 | 1 |
| MPC1 | 0.571852 | -0.296929 | 0.138 | 0.113 | 1 |
| ZER1 | 0.111671 | -0.296935 | 0.09 | 0.126 | 1 |
| SLC27A1 | 0.451079 | -0.298291 | 0.115 | 0.088 | 1 |
| MFSD10 | 0.598818 | -0.298402 | 0.214 | 0.189 | 1 |
| GOLGB1 | 0.055321 | -0.298484 | 0.16 | 0.094 | 1 |
| MX1 | 0.127373 | -0.2988 | 0.146 | 0.094 | 1 |
| DMKN | 0.448726 | -0.299107 | 0.406 | 0.333 | 1 |
| SRSF3 | 0.173919 | -0.299632 | 0.47 | 0.358 | 1 |
| PIN4 | 0.086684 | -0.300404 | 0.119 | 0.069 | 1 |
| TAP1 | 0.067043 | -0.300597 | 0.178 | 0.107 | 1 |
| C2CD5 | 0.629216 | -0.300937 | 0.11 | 0.094 | 1 |
| PSMB9 | 0.266916 | -0.30199 | 0.126 | 0.151 | 1 |
| COPS8 | 0.905568 | -0.301992 | 0.116 | 0.113 | 1 |
| NUMB | 0.94736 | -0.302731 | 0.131 | 0.126 | 1 |
| TUFM | 0.176004 | -0.302947 | 0.367 | 0.283 | 1 |
| GAK | 0.287428 | -0.302958 | 0.116 | 0.082 | 1 |
| UBE2E1 | 0.761722 | -0.30325 | 0.273 | 0.239 | 1 |
| GPR85 | 0.299904 | -0.304238 | 0.353 | 0.358 | 1 |
| SFN | 0.668146 | -0.306421 | 0.126 | 0.132 | 1 |
| TMEM59 | 0.559366 | -0.306471 | 0.501 | 0.447 | 1 |
| SMDT1 | 0.283518 | -0.307081 | 0.247 | 0.182 | 1 |
| OXR1 | 0.951172 | -0.307128 | 0.111 | 0.107 | 1 |
| DAB2IP | 0.134224 | -0.307175 | 0.121 | 0.075 | 1 |
| EXOC4 | 0.642175 | -0.307658 | 0.106 | 0.113 | 1 |
| SUPT20H | 0.851963 | -0.308218 | 0.109 | 0.101 | 1 |
| TAF6 | 0.806238 | -0.308236 | 0.121 | 0.107 | 1 |
| COL6A1 | 0.01912 | -0.308517 | 0.156 | 0.082 | 1 |
| ATXN7L3B | 0.210372 | -0.308585 | 0.152 | 0.182 | 1 |
| SARNP | 0.051153 | -0.309039 | 0.212 | 0.132 | 1 |
| PPP6R1 | 0.233562 | -0.30936 | 0.122 | 0.151 | 1 |
| TMEM41B | 0.143073 | -0.310879 | 0.105 | 0.063 | 1 |
| CCT6A | 0.182967 | -0.311002 | 0.296 | 0.22 | 1 |
| ZNF302 | 0.133562 | -0.31105 | 0.523 | 0.541 | 1 |
| SOD3 | 0.156621 | -0.311665 | 0.07 | 0.101 | 1 |
| MDH1 | 0.001639 | -0.312038 | 0.284 | 0.145 | 1 |
| WDR13 | 0.151508 | -0.31204 | 0.195 | 0.226 | 1 |
| SATB1 | 0.084751 | -0.312324 | 0.077 | 0.113 | 1 |
| UCKL1 | 0.114946 | -0.312815 | 0.156 | 0.101 | 1 |
| CTDSP2 | 0.049611 | -0.31304 | 0.231 | 0.145 | 1 |
| TSC2 | 0.144874 | -0.313097 | 0.159 | 0.107 | 1 |
| PTBP3 | 0.595905 | -0.313663 | 0.156 | 0.164 | 1 |
| TAGLN | 0.671086 | -0.315398 | 0.105 | 0.113 | 1 |
| BSG | 0.641875 | -0.315464 | 0.112 | 0.119 | 1 |
| WRB | 0.011059 | -0.316172 | 0.056 | 0.107 | 1 |
| H2AFY | 0.300041 | -0.31636 | 0.31 | 0.239 | 1 |
| PPAPDC1B | 0.074239 | -0.316489 | 0.152 | 0.201 | 1 |
| CTNNA1 | 0.437487 | -0.317045 | 0.369 | 0.358 | 1 |
| TMED1 | 0.899469 | -0.317345 | 0.109 | 0.101 | 1 |
| UBL3 | 0.251416 | -0.317676 | 0.128 | 0.151 | 1 |
| EPS8L2 | 0.315118 | -0.31772 | 0.254 | 0.201 | 1 |
| GSE1 | 0.888661 | -0.317969 | 0.144 | 0.132 | 1 |
| TRPS1 | 0.832975 | -0.319132 | 0.123 | 0.113 | 1 |
| JUN | 0.761126 | -0.319307 | 0.756 | 0.648 | 1 |
| SOCS1 | 0.025088 | -0.320201 | 0.06 | 0.107 | 1 |
| TACC1 | 0.159915 | -0.320361 | 0.152 | 0.101 | 1 |
| NSFL1C | 0.214673 | -0.320424 | 0.153 | 0.182 | 1 |
| ATF7IP | 0.728631 | -0.320513 | 0.123 | 0.107 | 1 |
| KIAA0196 | 0.049125 | -0.321984 | 0.128 | 0.069 | 1 |
| THNSL2 | 0.160228 | -0.323294 | 0.072 | 0.101 | 1 |
| TSNARE1 | 0.47208 | -0.323299 | 0.086 | 0.101 | 1 |
| PKN2 | 0.34196 | -0.323998 | 0.117 | 0.088 | 1 |
| SLC25A24 | 0.494973 | -0.324345 | 0.101 | 0.113 | 1 |
| COX19 | 0.030909 | -0.325207 | 0.093 | 0.145 | 1 |
| PIAS3 | 0.261357 | -0.325291 | 0.117 | 0.145 | 1 |
| PRDX1 | 0.985047 | -0.32593 | 0.49 | 0.415 | 1 |
| HIAT1 | 0.11332 | -0.326707 | 0.125 | 0.075 | 1 |
| YPEL5 | 0.74701 | -0.326736 | 0.214 | 0.214 | 1 |
| KIF3B | 0.929085 | -0.327043 | 0.121 | 0.119 | 1 |
| MPG | 0.172377 | -0.327494 | 0.141 | 0.094 | 1 |
| IRF9 | 0.792014 | -0.327706 | 0.181 | 0.164 | 1 |
| CIR1 | 0.547656 | -0.328035 | 0.133 | 0.145 | 1 |
| VRK3 | 0.028515 | -0.328112 | 0.057 | 0.101 | 1 |
| USP25 | 0.487618 | -0.328761 | 0.117 | 0.094 | 1 |
| PCM1 | 0.333457 | -0.329216 | 0.141 | 0.164 | 1 |
| HLA.E | 0.359282 | -0.329254 | 0.495 | 0.478 | 1 |
| COQ9 | 0.511254 | -0.329294 | 0.13 | 0.107 | 1 |
| TC2N | 0.73572 | -0.329619 | 0.163 | 0.164 | 1 |
| UTRN | 0.073929 | -0.330591 | 0.123 | 0.069 | 1 |
| SQLE | 0.23536 | -0.330616 | 0.144 | 0.176 | 1 |
| PAK2 | 0.414079 | -0.33064 | 0.101 | 0.119 | 1 |
| CHD4 | 0.403268 | -0.330768 | 0.378 | 0.314 | 1 |
| SULT1A1 | 0.412066 | -0.331461 | 0.081 | 0.101 | 1 |
| RBM8A | 0.214486 | -0.331808 | 0.315 | 0.239 | 1 |
| TMEM109 | 0.936406 | -0.332729 | 0.138 | 0.126 | 1 |
| SDCBP | 0.292746 | -0.333273 | 0.242 | 0.258 | 1 |
| TBCA | 0.650934 | -0.333363 | 0.225 | 0.189 | 1 |
| YIPF4 | 0.048441 | -0.333461 | 0.122 | 0.063 | 1 |
| CD24 | 0.103802 | -0.333821 | 0.667 | 0.597 | 1 |
| LMBRD1 | 0.995587 | -0.334026 | 0.133 | 0.126 | 1 |
| DNAL4 | 0.027258 | -0.334314 | 0.077 | 0.126 | 1 |
| NDUFC1 | 0.858733 | -0.33581 | 0.346 | 0.314 | 1 |
| CTTN | 0.743162 | -0.335987 | 0.352 | 0.333 | 1 |
| TTC9 | 0.059264 | -0.337308 | 0.067 | 0.107 | 1 |
| GRAMD1C | 0.014035 | -0.339271 | 0.079 | 0.138 | 1 |
| SIGIRR | 0.531573 | -0.339704 | 0.21 | 0.176 | 1 |
| TMEM115 | 0.23914 | -0.340189 | 0.119 | 0.082 | 1 |
| FAM208B | 0.16534 | -0.34043 | 0.101 | 0.063 | 1 |
| DAZAP2 | 0.325309 | -0.341489 | 0.391 | 0.384 | 1 |
| ARFGEF2 | 0.556939 | -0.34149 | 0.102 | 0.082 | 1 |
| TFAP2C | 0.032 | -0.341742 | 0.107 | 0.05 | 1 |
| EHMT1 | 0.216408 | -0.342132 | 0.115 | 0.075 | 1 |
| RUVBL2 | 0.018549 | -0.34259 | 0.138 | 0.201 | 1 |
| ABR | 9.66E-05 | -0.343536 | 0.04 | 0.113 | 1 |
| SLC30A5 | 0.989874 | -0.343697 | 0.12 | 0.113 | 1 |
| TSTD1 | 0.224713 | -0.343725 | 0.367 | 0.365 | 1 |
| OSBPL3 | 0.778516 | -0.345653 | 0.151 | 0.138 | 1 |
| TPGS2 | 0.380344 | -0.345697 | 0.156 | 0.119 | 1 |
| SSB | 0.977755 | -0.346147 | 0.204 | 0.189 | 1 |
| RARS | 0.93052 | -0.346422 | 0.107 | 0.101 | 1 |
| CD27.AS1 | 0.238784 | -0.346782 | 0.12 | 0.082 | 1 |
| ISCU | 0.568266 | -0.348532 | 0.173 | 0.176 | 1 |
| UQCRC1 | 0.859908 | -0.34957 | 0.29 | 0.258 | 1 |
| EMX2OS | 0.432541 | -0.349884 | 0.127 | 0.145 | 1 |
| PMM1 | 0.01975 | -0.349991 | 0.059 | 0.107 | 1 |
| DRAM2 | 0.593271 | -0.350191 | 0.11 | 0.119 | 1 |
| ABHD2 | 0.059802 | -0.350819 | 0.068 | 0.107 | 1 |
| 15-Sep | 0.069885 | -0.350928 | 0.425 | 0.296 | 1 |
| POLR2A | 0.33763 | -0.350945 | 0.181 | 0.201 | 1 |
| STUB1 | 0.871333 | -0.351351 | 0.186 | 0.182 | 1 |
| UXT | 0.383961 | -0.351625 | 0.252 | 0.201 | 1 |
| MORF4L2 | 0.497625 | -0.351641 | 0.359 | 0.34 | 1 |
| HMGB2 | 0.120058 | -0.351894 | 0.268 | 0.308 | 1 |
| CABIN1 | 0.906398 | -0.351988 | 0.101 | 0.101 | 1 |
| TEKT2 | 4.49E-13 | -0.352109 | 0.021 | 0.145 | 7.62E-09 |
| ASB8 | 0.105143 | -0.352559 | 0.11 | 0.063 | 1 |
| WBP11 | 0.911466 | -0.352834 | 0.18 | 0.17 | 1 |
| PAPOLA | 0.263594 | -0.354676 | 0.305 | 0.321 | 1 |
| H1FX | 0.876917 | -0.355123 | 0.353 | 0.308 | 1 |
| LOC100132215 | 0.013444 | -0.356053 | 0.59 | 0.604 | 1 |
| DAPK1 | 0.329762 | -0.35634 | 0.117 | 0.088 | 1 |
| CUL4A | 0.509418 | -0.356487 | 0.132 | 0.107 | 1 |
| TJP2 | 0.545306 | -0.356544 | 0.223 | 0.189 | 1 |
| SKP1 | 0.466692 | -0.35655 | 0.425 | 0.396 | 1 |
| STYXL1 | 0.052468 | -0.356759 | 0.104 | 0.151 | 1 |
| TERF2IP | 0.198261 | -0.357576 | 0.135 | 0.164 | 1 |
| SSU72 | 0.354659 | -0.357631 | 0.256 | 0.208 | 1 |
| BTG2 | 0.881344 | -0.357919 | 0.544 | 0.459 | 1 |
| 6-Mar | 0.272216 | -0.358312 | 0.356 | 0.277 | 1 |
| ZC2HC1A | 0.043565 | -0.358654 | 0.059 | 0.101 | 1 |
| DAD1 | 0.6244 | -0.358724 | 0.38 | 0.314 | 1 |
| FAM107B | 0.620308 | -0.359382 | 0.135 | 0.138 | 1 |
| STAU1 | 0.424127 | -0.359489 | 0.199 | 0.157 | 1 |
| AKAP13 | 0.543154 | -0.359774 | 0.149 | 0.157 | 1 |
| EMP2 | 0.342845 | -0.360688 | 0.326 | 0.321 | 1 |
| ZDHHC6 | 0.017596 | -0.360733 | 0.127 | 0.057 | 1 |
| MAEA | 0.627894 | -0.361628 | 0.121 | 0.101 | 1 |
| CDC42EP3 | 0.915723 | -0.362195 | 0.121 | 0.113 | 1 |
| STK24 | 0.130713 | -0.362447 | 0.19 | 0.132 | 1 |
| FBXO33 | 0.288466 | -0.362473 | 0.095 | 0.119 | 1 |
| GDE1 | 0.935166 | -0.362533 | 0.138 | 0.126 | 1 |
| BUD31 | 0.424737 | -0.362668 | 0.314 | 0.252 | 1 |
| MID1IP1 | 0.249507 | -0.363674 | 0.123 | 0.151 | 1 |
| UNC50 | 0.524522 | -0.36466 | 0.125 | 0.101 | 1 |
| POR | 0.549246 | -0.365052 | 0.269 | 0.22 | 1 |
| CDK9 | 0.616579 | -0.365511 | 0.098 | 0.107 | 1 |
| CYB561A3 | 0.05283 | -0.365685 | 0.062 | 0.101 | 1 |
| RTFDC1 | 0.919212 | -0.365781 | 0.164 | 0.157 | 1 |
| WRNIP1 | 0.785131 | -0.367436 | 0.184 | 0.164 | 1 |
| MIR5684 | 0.433924 | -0.367798 | 0.181 | 0.195 | 1 |
| HSPH1 | 0.182665 | -0.368136 | 0.368 | 0.396 | 1 |
| ZMYND8 | 0.200722 | -0.369387 | 0.246 | 0.182 | 1 |
| ORMDL2 | 0.0321 | -0.369672 | 0.089 | 0.138 | 1 |
| MAP1LC3B | 0.665589 | -0.370144 | 0.21 | 0.208 | 1 |
| RAF1 | 0.21666 | -0.371023 | 0.159 | 0.113 | 1 |
| UBN2 | 0.165628 | -0.371181 | 0.07 | 0.101 | 1 |
| TMED4 | 0.828998 | -0.37166 | 0.248 | 0.214 | 1 |
| GLO1 | 0.553542 | -0.372017 | 0.194 | 0.157 | 1 |
| SMIM22 | 0.266794 | -0.372174 | 0.304 | 0.308 | 1 |
| EEF2K | 0.143837 | -0.372891 | 0.099 | 0.132 | 1 |
| GOLGA2 | 0.268062 | -0.373034 | 0.132 | 0.094 | 1 |
| CASC4 | 0.601464 | -0.373061 | 0.196 | 0.201 | 1 |
| SECISBP2L | 0.799547 | -0.37307 | 0.106 | 0.094 | 1 |
| NPIPA5 | 0.169087 | -0.373576 | 0.112 | 0.145 | 1 |
| NRAV | 5.58E-08 | -0.374802 | 0.026 | 0.119 | 0.000948 |
| SAP18 | 1 | -0.3749 | 0.354 | 0.308 | 1 |
| ANKRD54 | 5.58E-05 | -0.375575 | 0.038 | 0.113 | 0.947502 |
| ACADVL | 0.71021 | -0.375653 | 0.388 | 0.327 | 1 |
| CELSR1 | 0.559037 | -0.375798 | 0.144 | 0.119 | 1 |
| KCNJ3 | 0.236804 | -0.376138 | 0.223 | 0.252 | 1 |
| GTF2A2 | 0.006559 | -0.376189 | 0.207 | 0.107 | 1 |
| ANKRD28 | 0.497018 | -0.376427 | 0.123 | 0.101 | 1 |
| MPZL3 | 0.016474 | -0.376708 | 0.053 | 0.101 | 1 |
| C1orf56 | 0.291204 | -0.37759 | 0.114 | 0.082 | 1 |
| ANKRD10 | 0.118407 | -0.377668 | 0.333 | 0.239 | 1 |
| GSR | 0.383459 | -0.378021 | 0.162 | 0.126 | 1 |
| SCCPDH | 0.139945 | -0.378034 | 0.109 | 0.145 | 1 |
| CROCCP2 | 0.270668 | -0.378278 | 0.083 | 0.107 | 1 |
| CHMP5 | 0.796 | -0.379212 | 0.212 | 0.201 | 1 |
| YAP1 | 0.097012 | -0.379279 | 0.217 | 0.145 | 1 |
| RABL2B | 7.43E-10 | -0.379541 | 0.046 | 0.176 | 1.26E-05 |
| XPR1 | 0.275646 | -0.380477 | 0.154 | 0.113 | 1 |
| PER2 | 0.741792 | -0.380827 | 0.142 | 0.126 | 1 |
| DCAF11 | 0.917064 | -0.380955 | 0.151 | 0.145 | 1 |
| CCT5 | 0.130204 | -0.381153 | 0.335 | 0.245 | 1 |
| TCEB1 | 0.054832 | -0.381566 | 0.305 | 0.214 | 1 |
| PSMD2 | 0.291413 | -0.382041 | 0.215 | 0.164 | 1 |
| CDKN1B | 0.447657 | -0.382143 | 0.104 | 0.082 | 1 |
| UBE2A | 0.607755 | -0.382269 | 0.131 | 0.138 | 1 |
| MGMT | 0.018896 | -0.382483 | 0.143 | 0.208 | 1 |
| RHOU | 0.004679 | -0.382917 | 0.136 | 0.214 | 1 |
| INTS10 | 0.873974 | -0.383253 | 0.115 | 0.113 | 1 |
| HLA.J | 0.953605 | -0.383752 | 0.154 | 0.145 | 1 |
| DRC7 | 3.91E-14 | -0.383758 | 0.007 | 0.107 | 6.63E-10 |
| POC1B | 0.303253 | -0.383846 | 0.085 | 0.107 | 1 |
| SNX2 | 0.871771 | -0.384348 | 0.135 | 0.132 | 1 |
| VPS37B | 0.893535 | -0.384437 | 0.131 | 0.119 | 1 |
| ATXN2 | 0.184329 | -0.385035 | 0.146 | 0.101 | 1 |
| ZC3H11A | 0.292156 | -0.385154 | 0.198 | 0.151 | 1 |
| C4orf3 | 0.681969 | -0.385418 | 0.322 | 0.302 | 1 |
| USP8 | 0.264228 | -0.385772 | 0.084 | 0.107 | 1 |
| ALKBH5 | 0.888067 | -0.385802 | 0.122 | 0.113 | 1 |
| ZNF106 | 0.702413 | -0.386324 | 0.095 | 0.101 | 1 |
| RBM5 | 0.294234 | -0.386334 | 0.315 | 0.245 | 1 |
| LUC7L | 0.115179 | -0.386826 | 0.184 | 0.119 | 1 |
| EIF2AK1 | 0.585115 | -0.386943 | 0.205 | 0.17 | 1 |
| PHIP | 0.531718 | -0.387655 | 0.21 | 0.176 | 1 |
| PLTP | 0.106608 | -0.388366 | 0.199 | 0.239 | 1 |
| ST6GALNAC1 | 0.71927 | -0.388388 | 0.11 | 0.113 | 1 |
| PTP4A1 | 0.690096 | -0.389229 | 0.316 | 0.308 | 1 |
| DUSP22 | 0.013715 | -0.389268 | 0.057 | 0.107 | 1 |
| PURA | 0.716203 | -0.389634 | 0.109 | 0.094 | 1 |
| HDLBP | 0.150167 | -0.389987 | 0.454 | 0.333 | 1 |
| ARIH1 | 0.072261 | -0.390305 | 0.14 | 0.082 | 1 |
| TMEM258 | 0.519096 | -0.390411 | 0.33 | 0.264 | 1 |
| TXNRD1 | 0.486589 | -0.391086 | 0.158 | 0.17 | 1 |
| DDB1 | 0.65901 | -0.391495 | 0.296 | 0.252 | 1 |
| FBXO2 | 0.174509 | -0.391557 | 0.073 | 0.101 | 1 |
| POFUT2 | 0.051187 | -0.391698 | 0.091 | 0.138 | 1 |
| KIAA1671 | 0.115924 | -0.39174 | 0.122 | 0.075 | 1 |
| POLR1C | 0.600355 | -0.393238 | 0.115 | 0.094 | 1 |
| ALCAM | 0.161573 | -0.393353 | 0.156 | 0.195 | 1 |
| TJP3 | 0.013651 | -0.393838 | 0.08 | 0.138 | 1 |
| TRIM2 | 0.401644 | -0.393876 | 0.122 | 0.138 | 1 |
| CES2 | 0.041796 | -0.394023 | 0.084 | 0.132 | 1 |
| DNAJB2 | 0.137325 | -0.396048 | 0.122 | 0.157 | 1 |
| PCMT1 | 0.024437 | -0.396663 | 0.158 | 0.082 | 1 |
| VCP | 0.502893 | -0.397421 | 0.331 | 0.27 | 1 |
| ABCA8 | 0.654149 | -0.398056 | 0.18 | 0.182 | 1 |
| IL6R | 0.023036 | -0.399275 | 0.112 | 0.17 | 1 |
| VPS35 | 0.4144 | -0.399889 | 0.193 | 0.208 | 1 |
| WDTC1 | 0.0189 | -0.400573 | 0.064 | 0.113 | 1 |
| HBEGF | 0.009381 | -0.402091 | 0.133 | 0.057 | 1 |
| UBALD2 | 0.258934 | -0.402975 | 0.246 | 0.27 | 1 |
| DCBLD2 | 0.799622 | -0.40308 | 0.119 | 0.119 | 1 |
| MICALL2 | 0.246814 | -0.404852 | 0.141 | 0.101 | 1 |
| QKI | 0.07409 | -0.404879 | 0.141 | 0.082 | 1 |
| RTN4 | 0.764329 | -0.406218 | 0.356 | 0.302 | 1 |
| TMEM68 | 0.001119 | -0.406305 | 0.085 | 0.164 | 1 |
| SPATA6L | 8.11E-05 | -0.407193 | 0.032 | 0.101 | 1 |
| ARID1B | 0.70799 | -0.408417 | 0.101 | 0.088 | 1 |
| ERP29 | 0.428281 | -0.409019 | 0.362 | 0.289 | 1 |
| PHKG1 | 0.189397 | -0.409326 | 0.096 | 0.126 | 1 |
| CAPNS1 | 0.870808 | -0.409952 | 0.553 | 0.472 | 1 |
| RHPN2 | 0.349388 | -0.410041 | 0.159 | 0.176 | 1 |
| IRF7 | 0.022818 | -0.410333 | 0.193 | 0.107 | 1 |
| MAML2 | 0.139233 | -0.411654 | 0.074 | 0.107 | 1 |
| UBA1 | 0.604551 | -0.411882 | 0.443 | 0.409 | 1 |
| FXR1 | 0.312066 | -0.41211 | 0.177 | 0.132 | 1 |
| PLEKHB1 | 0.00982 | -0.41236 | 0.059 | 0.113 | 1 |
| TSPAN3 | 0.702959 | -0.412732 | 0.537 | 0.491 | 1 |
| SPR | 0.450679 | -0.413021 | 0.086 | 0.101 | 1 |
| RFX1 | 0.037276 | -0.413181 | 0.069 | 0.113 | 1 |
| SPATS2L | 0.13338 | -0.414147 | 0.175 | 0.214 | 1 |
| USP11 | 0.883735 | -0.41504 | 0.125 | 0.113 | 1 |
| PACSIN2 | 0.704436 | -0.415284 | 0.101 | 0.088 | 1 |
| DIDO1 | 0.622871 | -0.416167 | 0.105 | 0.088 | 1 |
| TPRG1L | 0.541479 | -0.4162 | 0.115 | 0.126 | 1 |
| BAG6 | 0.148401 | -0.416753 | 0.278 | 0.302 | 1 |
| SETD3 | 0.922109 | -0.417243 | 0.109 | 0.101 | 1 |
| LIMS1 | 0.58694 | -0.417461 | 0.122 | 0.132 | 1 |
| ARMC9 | 4.98E-05 | -0.418448 | 0.031 | 0.101 | 0.845632 |
| LAP3 | 0.780078 | -0.41938 | 0.164 | 0.164 | 1 |
| ATP6V0C | 0.306893 | -0.42015 | 0.525 | 0.478 | 1 |
| PAN3 | 0.606111 | -0.420297 | 0.126 | 0.107 | 1 |
| UBC | 6.11E-07 | -0.421823 | 0.819 | 0.868 | 0.010379 |
| RRM2B | 0.264337 | -0.421856 | 0.089 | 0.113 | 1 |
| NOL7 | 0.058567 | -0.423037 | 0.149 | 0.088 | 1 |
| AIG1 | 0.057069 | -0.4234 | 0.109 | 0.057 | 1 |
| LOC100506844 | 2.88E-08 | -0.423534 | 0.028 | 0.126 | 0.000488 |
| LGALS3 | 0.920906 | -0.423651 | 0.46 | 0.396 | 1 |
| PFKFB3 | 0.802656 | -0.423872 | 0.23 | 0.201 | 1 |
| PPP4R3A | 0.450636 | -0.424937 | 0.157 | 0.17 | 1 |
| GLA | 0.029359 | -0.425196 | 0.062 | 0.107 | 1 |
| HIPK2 | 0.405037 | -0.425279 | 0.146 | 0.113 | 1 |
| POLR2I | 0.104043 | -0.425468 | 0.306 | 0.34 | 1 |
| UBR4 | 0.953373 | -0.427073 | 0.13 | 0.119 | 1 |
| ALDH1L1 | 6.87E-15 | -0.427085 | 0.005 | 0.101 | 1.17E-10 |
| AZIN1 | 0.604024 | -0.427793 | 0.321 | 0.302 | 1 |
| COX17 | 0.629144 | -0.428171 | 0.169 | 0.145 | 1 |
| GNG12 | 0.31396 | -0.428191 | 0.169 | 0.189 | 1 |
| SNX17 | 0.600367 | -0.428562 | 0.141 | 0.145 | 1 |
| H2AFJ | 0.407176 | -0.430214 | 0.228 | 0.239 | 1 |
| TMEM222 | 0.02556 | -0.430594 | 0.07 | 0.119 | 1 |
| VEZF1 | 0.31244 | -0.430802 | 0.149 | 0.17 | 1 |
| MICAL3 | 0.042067 | -0.431375 | 0.075 | 0.119 | 1 |
| REPIN1 | 0.168131 | -0.431755 | 0.233 | 0.264 | 1 |
| AKAP8 | 0.910435 | -0.431987 | 0.117 | 0.107 | 1 |
| SLC2A1 | 0.506734 | -0.432042 | 0.332 | 0.327 | 1 |
| RGCC | 0.443152 | -0.432776 | 0.189 | 0.201 | 1 |
| PER1 | 0.762558 | -0.433713 | 0.206 | 0.182 | 1 |
| YY1AP1 | 0.937504 | -0.434061 | 0.117 | 0.113 | 1 |
| CHP1 | 0.058821 | -0.434169 | 0.174 | 0.107 | 1 |
| NDUFAB1 | 0.755021 | -0.434943 | 0.263 | 0.245 | 1 |
| NUTF2 | 0.059238 | -0.435068 | 0.127 | 0.069 | 1 |
| CLDN9 | 2.28E-07 | -0.435696 | 0.026 | 0.113 | 0.003865 |
| ITGB4 | 0.460451 | -0.436002 | 0.294 | 0.296 | 1 |
| ZNF330 | 0.353997 | -0.436472 | 0.14 | 0.107 | 1 |
| OGFOD2 | 0.038069 | -0.437516 | 0.058 | 0.101 | 1 |
| CLN5 | 0.335648 | -0.437806 | 0.117 | 0.138 | 1 |
| RAB14 | 0.957276 | -0.437928 | 0.156 | 0.145 | 1 |
| ETNK1 | 0.614404 | -0.43805 | 0.163 | 0.138 | 1 |
| TNFRSF19 | 3.28E-06 | -0.439041 | 0.031 | 0.113 | 0.055726 |
| CLDN4 | 0.052805 | -0.439127 | 0.636 | 0.61 | 1 |
| DHX40 | 0.848514 | -0.439732 | 0.119 | 0.107 | 1 |
| MED25 | 0.073133 | -0.439895 | 0.147 | 0.195 | 1 |
| REXO2 | 0.472468 | -0.440377 | 0.169 | 0.138 | 1 |
| CCDC103 | 2.93E-09 | -0.441112 | 0.015 | 0.101 | 4.97E-05 |
| PHAX | 0.643134 | -0.441844 | 0.109 | 0.113 | 1 |
| RAPGEF2 | 0.039631 | -0.442571 | 0.068 | 0.113 | 1 |
| CAB39 | 0.755058 | -0.443151 | 0.14 | 0.138 | 1 |
| SLC41A3 | 0.202872 | -0.444697 | 0.109 | 0.138 | 1 |
| ENPP5 | 0.00107 | -0.444983 | 0.044 | 0.107 | 1 |
| MYO1E | 0.00224 | -0.445206 | 0.052 | 0.113 | 1 |
| ATP6AP1 | 0.644742 | -0.445286 | 0.273 | 0.264 | 1 |
| NPIPA1 | 0.010373 | -0.445361 | 0.099 | 0.164 | 1 |
| ENAH | 0.000832 | -0.445842 | 0.164 | 0.264 | 1 |
| PITPNA | 0.872281 | -0.447508 | 0.174 | 0.157 | 1 |
| CBFB | 0.597727 | -0.44791 | 0.112 | 0.094 | 1 |
| EXOC5 | 0.07444 | -0.448101 | 0.07 | 0.107 | 1 |
| IFITM10 | 0.000675 | -0.448493 | 0.043 | 0.107 | 1 |
| TNK2 | 0.150114 | -0.450588 | 0.121 | 0.075 | 1 |
| DPM1 | 0.534203 | -0.451308 | 0.125 | 0.101 | 1 |
| SRD5A3 | 0.764544 | -0.453069 | 0.104 | 0.107 | 1 |
| RIIAD1 | 1.76E-20 | -0.453111 | 0.006 | 0.138 | 2.99E-16 |
| ICMT | 0.499174 | -0.454521 | 0.111 | 0.088 | 1 |
| TEAD1 | 0.232256 | -0.454825 | 0.11 | 0.075 | 1 |
| DDX3X | 0.960592 | -0.455907 | 0.405 | 0.365 | 1 |
| NEAT1 | 0.00284 | -0.45667 | 0.838 | 0.811 | 1 |
| LINC01578 | 0.436025 | -0.45692 | 0.381 | 0.308 | 1 |
| BAIAP2 | 0.60867 | -0.458544 | 0.328 | 0.321 | 1 |
| CEP70 | 0.036532 | -0.459189 | 0.059 | 0.101 | 1 |
| ZFAND6 | 0.005593 | -0.459559 | 0.16 | 0.069 | 1 |
| KIF9 | 0.026998 | -0.460582 | 0.107 | 0.164 | 1 |
| BBIP1 | 0.011645 | -0.460821 | 0.052 | 0.101 | 1 |
| PRKAA1 | 0.249608 | -0.462719 | 0.077 | 0.101 | 1 |
| C11orf49 | 0.011333 | -0.463339 | 0.105 | 0.17 | 1 |
| ANK3 | 0.084507 | -0.463993 | 0.093 | 0.132 | 1 |
| HIST1H1C | 0.026252 | -0.46413 | 0.122 | 0.182 | 1 |
| CEP164 | 0.142997 | -0.464414 | 0.111 | 0.145 | 1 |
| ATP1B1 | 0.57699 | -0.465284 | 0.251 | 0.258 | 1 |
| TLE4 | 0.000172 | -0.466663 | 0.038 | 0.107 | 1 |
| PWWP2B | 0.945216 | -0.467677 | 0.121 | 0.113 | 1 |
| FAM13A | 0.944527 | -0.46854 | 0.102 | 0.101 | 1 |
| FDXR | 0.001006 | -0.468714 | 0.069 | 0.145 | 1 |
| TAGLN2 | 0.017154 | -0.469149 | 0.502 | 0.491 | 1 |
| WDR73 | 0.257382 | -0.469459 | 0.101 | 0.126 | 1 |
| ABI1 | 0.077973 | -0.470874 | 0.133 | 0.176 | 1 |
| PSMB5 | 0.404279 | -0.472914 | 0.277 | 0.214 | 1 |
| KDM1A | 0.775767 | -0.473125 | 0.158 | 0.138 | 1 |
| METTL7A | 0.33868 | -0.474452 | 0.178 | 0.195 | 1 |
| RPRD2 | 0.599542 | -0.476415 | 0.147 | 0.119 | 1 |
| SPAG16 | 0.00393 | -0.476648 | 0.098 | 0.17 | 1 |
| UBE2J1 | 0.574099 | -0.477644 | 0.122 | 0.101 | 1 |
| C5orf15 | 0.305998 | -0.479356 | 0.184 | 0.201 | 1 |
| KAZN | 0.174305 | -0.480345 | 0.126 | 0.157 | 1 |
| RRAGA | 0.105369 | -0.481401 | 0.119 | 0.157 | 1 |
| OXTR | 3.30E-08 | -0.481519 | 0.028 | 0.126 | 0.000561 |
| SMAP2 | 0.03165 | -0.481658 | 0.058 | 0.101 | 1 |
| UBAP1 | 0.271315 | -0.482511 | 0.138 | 0.101 | 1 |
| CFDP1 | 0.003503 | -0.483129 | 0.098 | 0.17 | 1 |
| TMBIM4 | 0.298233 | -0.484159 | 0.244 | 0.189 | 1 |
| FAM214A | 0.079037 | -0.48435 | 0.133 | 0.075 | 1 |
| C2CD2L | 4.29E-07 | -0.485534 | 0.049 | 0.157 | 0.007288 |
| NDUFB5 | 0.955393 | -0.486544 | 0.264 | 0.233 | 1 |
| DDR1 | 0.233522 | -0.486579 | 0.37 | 0.384 | 1 |
| PPP1R7 | 0.024936 | -0.487067 | 0.144 | 0.201 | 1 |
| YBX3 | 0.024912 | -0.490138 | 0.501 | 0.497 | 1 |
| GTF2F1 | 0.872105 | -0.490162 | 0.119 | 0.107 | 1 |
| TBC1D8 | 0.043529 | -0.490312 | 0.128 | 0.176 | 1 |
| STAM2 | 0.186258 | -0.49041 | 0.08 | 0.107 | 1 |
| CEBPG | 0.453637 | -0.491664 | 0.131 | 0.145 | 1 |
| ATP6V0D1 | 0.0043 | -0.493859 | 0.099 | 0.17 | 1 |
| JMJD1C | 0.273665 | -0.493909 | 0.141 | 0.164 | 1 |
| ATAD3A | 0.059412 | -0.494851 | 0.072 | 0.113 | 1 |
| RABL6 | 0.209168 | -0.496015 | 0.152 | 0.107 | 1 |
| CAPN2 | 0.375125 | -0.49692 | 0.348 | 0.346 | 1 |
| SNW1 | 0.603037 | -0.497462 | 0.106 | 0.113 | 1 |
| C14orf132 | 4.64E-06 | -0.498722 | 0.042 | 0.132 | 0.07875 |
| CALML4 | 0.004027 | -0.499525 | 0.064 | 0.126 | 1 |
| SREK1 | 0.729167 | -0.499724 | 0.175 | 0.176 | 1 |
| CNOT6 | 0.334992 | -0.499828 | 0.089 | 0.107 | 1 |
| TUBGCP2 | 0.032914 | -0.500985 | 0.133 | 0.189 | 1 |
| ROMO1 | 0.147302 | -0.501168 | 0.357 | 0.252 | 1 |
| SEC31A | 0.816709 | -0.501238 | 0.164 | 0.145 | 1 |
| ELOF1 | 0.439146 | -0.501322 | 0.147 | 0.157 | 1 |
| CLGN | 0.154498 | -0.501552 | 0.072 | 0.101 | 1 |
| CTGF | 0.29698 | -0.503911 | 0.331 | 0.264 | 1 |
| MLLT1 | 0.183796 | -0.504042 | 0.127 | 0.157 | 1 |
| RABL2A | 2.92E-09 | -0.504875 | 0.036 | 0.151 | 4.96E-05 |
| MKS1 | 0.002432 | -0.505961 | 0.043 | 0.101 | 1 |
| MAPRE3 | 1.21E-06 | -0.506085 | 0.026 | 0.107 | 0.020496 |
| C10orf10 | 0.131424 | -0.506257 | 0.194 | 0.233 | 1 |
| CTSL | 0.013252 | -0.506659 | 0.164 | 0.233 | 1 |
| GNB2 | 0.158713 | -0.506865 | 0.333 | 0.34 | 1 |
| ERICH2 | 9.20E-06 | -0.507342 | 0.041 | 0.126 | 0.156242 |
| MVP | 0.195722 | -0.509106 | 0.243 | 0.264 | 1 |
| F11R | 0.872931 | -0.509169 | 0.295 | 0.264 | 1 |
| CETN3 | 0.499916 | -0.509337 | 0.093 | 0.107 | 1 |
| PRRG4 | 0.001694 | -0.509754 | 0.094 | 0.17 | 1 |
| DEK | 0.20216 | -0.510569 | 0.265 | 0.195 | 1 |
| ISOC1 | 0.172599 | -0.511072 | 0.107 | 0.069 | 1 |
| CLHC1 | 0.001519 | -0.512316 | 0.057 | 0.126 | 1 |
| PIN1 | 0.227651 | -0.513976 | 0.132 | 0.157 | 1 |
| HLA.B | 0.030154 | -0.51401 | 0.758 | 0.723 | 1 |
| ACBD7 | 0.042667 | -0.514052 | 0.06 | 0.101 | 1 |
| MOGS | 0.005484 | -0.514808 | 0.125 | 0.044 | 1 |
| RABGAP1L | 0.019659 | -0.514849 | 0.083 | 0.138 | 1 |
| NELL2 | 3.23E-13 | -0.515647 | 0.012 | 0.119 | 5.48E-09 |
| RNF19A | 0.851298 | -0.516749 | 0.13 | 0.132 | 1 |
| FDX1 | 0.907702 | -0.519958 | 0.104 | 0.101 | 1 |
| CD99 | 0.742038 | -0.520339 | 0.193 | 0.195 | 1 |
| CNTRL | 7.19E-07 | -0.523055 | 0.022 | 0.101 | 0.012212 |
| LZTFL1 | 0.001846 | -0.523231 | 0.1 | 0.176 | 1 |
| HSPB1 | 8.49E-06 | -0.523402 | 0.79 | 0.767 | 0.144265 |
| CST6 | 2.22E-05 | -0.523557 | 0.032 | 0.107 | 0.377788 |
| EZR | 0.000727 | -0.523639 | 0.472 | 0.553 | 1 |
| LOC388780 | 1.38E-07 | -0.524412 | 0.02 | 0.101 | 0.002348 |
| ZCCHC11 | 0.402485 | -0.524446 | 0.109 | 0.082 | 1 |
| CFAP221 | 4.13E-17 | -0.525818 | 0.006 | 0.119 | 7.02E-13 |
| CELF1 | 0.916512 | -0.526362 | 0.263 | 0.239 | 1 |
| SLC25A4 | 0.354912 | -0.529736 | 0.149 | 0.164 | 1 |
| KIF5B | 0.753694 | -0.530511 | 0.237 | 0.22 | 1 |
| TRNAU1AP | 0.000146 | -0.530968 | 0.064 | 0.151 | 1 |
| ARL3 | 0.007839 | -0.531669 | 0.107 | 0.176 | 1 |
| CAT | 8.98E-05 | -0.532684 | 0.126 | 0.239 | 1 |
| SHANK2 | 0.000149 | -0.532927 | 0.058 | 0.138 | 1 |
| UNC93B1 | 0.08181 | -0.533535 | 0.178 | 0.22 | 1 |
| SOAT1 | 3.48E-06 | -0.535161 | 0.035 | 0.119 | 0.059048 |
| SOD1 | 0.393704 | -0.53632 | 0.489 | 0.447 | 1 |
| BIN3 | 0.151899 | -0.536494 | 0.084 | 0.113 | 1 |
| LKAAEAR1 | 3.37E-14 | -0.536835 | 0.012 | 0.126 | 5.73E-10 |
| GCLM | 1.19E-05 | -0.537858 | 0.077 | 0.182 | 0.202358 |
| WEE1 | 0.225847 | -0.538704 | 0.159 | 0.113 | 1 |
| JUNB | 0.003409 | -0.53919 | 0.707 | 0.717 | 1 |
| DNAJC10 | 0.303731 | -0.539442 | 0.209 | 0.226 | 1 |
| HOXC4 | 0.001286 | -0.539779 | 0.062 | 0.132 | 1 |
| CFLAR | 0.261626 | -0.541282 | 0.232 | 0.252 | 1 |
| KLHDC9 | 0.004701 | -0.541651 | 0.06 | 0.119 | 1 |
| AGTRAP | 0.021772 | -0.542808 | 0.169 | 0.233 | 1 |
| MFSD6 | 0.006532 | -0.543631 | 0.062 | 0.119 | 1 |
| GMPR | 0.454135 | -0.546759 | 0.116 | 0.132 | 1 |
| SERPINB6 | 0.002233 | -0.546812 | 0.202 | 0.289 | 1 |
| TULP3 | 0.000543 | -0.547235 | 0.075 | 0.157 | 1 |
| PLSCR1 | 0.055466 | -0.552842 | 0.249 | 0.302 | 1 |
| SRGAP2 | 0.000517 | -0.553406 | 0.049 | 0.119 | 1 |
| HLA.C | 0.730983 | -0.553408 | 0.535 | 0.465 | 1 |
| DYNC2LI1 | 0.00455 | -0.554158 | 0.085 | 0.151 | 1 |
| WDR60 | 0.153576 | -0.555017 | 0.106 | 0.138 | 1 |
| NARS | 0.84542 | -0.55542 | 0.246 | 0.226 | 1 |
| NHLRC4 | 3.59E-18 | -0.555631 | 0.004 | 0.113 | 6.09E-14 |
| FBRS | 0.317062 | -0.556836 | 0.159 | 0.176 | 1 |
| C21orf2 | 0.010503 | -0.557797 | 0.07 | 0.126 | 1 |
| SGMS2 | 0.017555 | -0.558906 | 0.084 | 0.138 | 1 |
| ARAP1 | 0.021182 | -0.558976 | 0.114 | 0.17 | 1 |
| MSH3 | 0.000121 | -0.55936 | 0.053 | 0.132 | 1 |
| ANXA2 | 0.005429 | -0.559986 | 0.531 | 0.541 | 1 |
| AHI1 | 0.86041 | -0.561113 | 0.141 | 0.126 | 1 |
| MXD4 | 0.809414 | -0.561605 | 0.151 | 0.138 | 1 |
| MLXIP | 0.792866 | -0.562718 | 0.206 | 0.182 | 1 |
| MAFK | 0.288725 | -0.566005 | 0.13 | 0.151 | 1 |
| PDLIM4 | 0.172836 | -0.568268 | 0.125 | 0.157 | 1 |
| ELF1 | 0.369933 | -0.568793 | 0.109 | 0.126 | 1 |
| ZNF703 | 0.037905 | -0.570139 | 0.13 | 0.182 | 1 |
| CCDC34 | 0.002111 | -0.570464 | 0.065 | 0.132 | 1 |
| CSNK1G2 | 0.566216 | -0.570757 | 0.156 | 0.164 | 1 |
| CDH1 | 0.125276 | -0.57106 | 0.301 | 0.327 | 1 |
| TPBG | 0.052917 | -0.57174 | 0.111 | 0.057 | 1 |
| B2M | 0.000325 | -0.571811 | 0.841 | 0.843 | 1 |
| TTC26 | 2.05E-06 | -0.572292 | 0.027 | 0.107 | 0.034897 |
| NPTN | 0.212254 | -0.573184 | 0.277 | 0.296 | 1 |
| CCDC39 | 1.65E-20 | -0.573787 | 0.002 | 0.119 | 2.80E-16 |
| CRISPLD1 | 2.91E-06 | -0.573812 | 0.025 | 0.101 | 0.049483 |
| GADD45G | 0.300406 | -0.573995 | 0.123 | 0.145 | 1 |
| MEAF6 | 0.63212 | -0.574086 | 0.188 | 0.189 | 1 |
| SPG20 | 0.124791 | -0.574257 | 0.104 | 0.138 | 1 |
| SMYD2 | 0.115325 | -0.574687 | 0.098 | 0.132 | 1 |
| LRRFIP1 | 0.115257 | -0.574777 | 0.364 | 0.377 | 1 |
| UBAC1 | 7.06E-05 | -0.576438 | 0.104 | 0.208 | 1 |
| BDH1 | 0.046538 | -0.576726 | 0.107 | 0.157 | 1 |
| IFT122 | 0.000848 | -0.577478 | 0.084 | 0.164 | 1 |
| DNM2 | 0.665045 | -0.581074 | 0.212 | 0.208 | 1 |
| CAST | 0.001235 | -0.581529 | 0.191 | 0.283 | 1 |
| S100A11 | 1.52E-05 | -0.582135 | 0.626 | 0.654 | 0.258848 |
| TTC25 | 1.62E-14 | -0.583894 | 0.01 | 0.119 | 2.76E-10 |
| ESYT1 | 0.147994 | -0.584368 | 0.137 | 0.17 | 1 |
| LRRC56 | 6.82E-09 | -0.584795 | 0.035 | 0.145 | 0.000116 |
| RPS27L | 0.479645 | -0.585365 | 0.21 | 0.214 | 1 |
| ADIPOR1 | 0.590133 | -0.585779 | 0.133 | 0.138 | 1 |
| COQ4 | 0.160783 | -0.58656 | 0.202 | 0.233 | 1 |
| CRY1 | 0.081454 | -0.589241 | 0.08 | 0.119 | 1 |
| TMEM107 | 3.55E-08 | -0.589714 | 0.041 | 0.151 | 0.000604 |
| CLUAP1 | 9.13E-07 | -0.589807 | 0.104 | 0.239 | 0.015504 |
| LAMC2 | 0.057247 | -0.589879 | 0.207 | 0.258 | 1 |
| TTC21B | 7.15E-08 | -0.590069 | 0.027 | 0.119 | 0.001214 |
| COMMD7 | 0.328265 | -0.591071 | 0.131 | 0.151 | 1 |
| DNPH1 | 0.096971 | -0.591224 | 0.301 | 0.321 | 1 |
| C10orf32 | 0.185107 | -0.591286 | 0.156 | 0.182 | 1 |
| MDM1 | 0.000114 | -0.591681 | 0.052 | 0.132 | 1 |
| ST6GALNAC2 | 0.001403 | -0.593023 | 0.107 | 0.189 | 1 |
| BTBD1 | 0.003194 | -0.593196 | 0.107 | 0.182 | 1 |
| HK1 | 0.761338 | -0.593547 | 0.122 | 0.107 | 1 |
| SH3BP4 | 0.607044 | -0.594678 | 0.127 | 0.107 | 1 |
| HSPBP1 | 6.25E-05 | -0.596261 | 0.127 | 0.239 | 1 |
| TPCN1 | 0.119239 | -0.596659 | 0.09 | 0.126 | 1 |
| ZDHHC1 | 6.40E-08 | -0.597276 | 0.046 | 0.157 | 0.001087 |
| HIPK3 | 0.058009 | -0.599157 | 0.159 | 0.208 | 1 |
| MTMR12 | 0.255191 | -0.599422 | 0.102 | 0.126 | 1 |
| PIGQ | 0.094846 | -0.601326 | 0.106 | 0.145 | 1 |
| CHORDC1 | 0.87795 | -0.601618 | 0.115 | 0.113 | 1 |
| TACSTD2 | 0.051685 | -0.603653 | 0.504 | 0.497 | 1 |
| SFXN3 | 0.007105 | -0.603938 | 0.049 | 0.101 | 1 |
| CYB561D2 | 0.196074 | -0.605268 | 0.106 | 0.069 | 1 |
| IGFBP5 | 0.000455 | -0.605764 | 0.162 | 0.27 | 1 |
| CRNDE | 0.02345 | -0.607963 | 0.062 | 0.107 | 1 |
| CDC16 | 0.913876 | -0.6087 | 0.173 | 0.157 | 1 |
| FOCAD | 0.013476 | -0.609257 | 0.062 | 0.113 | 1 |
| GLUL | 0.007826 | -0.609954 | 0.411 | 0.472 | 1 |
| MTURN | 0.116813 | -0.612709 | 0.079 | 0.113 | 1 |
| SLTM | 0.358398 | -0.613151 | 0.188 | 0.145 | 1 |
| ACYP1 | 0.014523 | -0.616433 | 0.078 | 0.132 | 1 |
| C20orf96 | 1.26E-10 | -0.616491 | 0.04 | 0.17 | 2.14E-06 |
| NAT14 | 0.089689 | -0.619891 | 0.105 | 0.145 | 1 |
| COPRS | 0.000526 | -0.62186 | 0.147 | 0.239 | 1 |
| NR4A3 | 0.088907 | -0.625948 | 0.104 | 0.145 | 1 |
| SYBU | 0.19969 | -0.626743 | 0.086 | 0.113 | 1 |
| MSRB1 | 0.003215 | -0.62838 | 0.044 | 0.101 | 1 |
| MINOS1 | 0.876954 | -0.628475 | 0.306 | 0.283 | 1 |
| JKAMP | 0.007816 | -0.628475 | 0.12 | 0.189 | 1 |
| GDI1 | 0.74238 | -0.630476 | 0.142 | 0.145 | 1 |
| RAB11FIP1 | 0.039099 | -0.63058 | 0.167 | 0.22 | 1 |
| CCP110 | 6.57E-05 | -0.633892 | 0.054 | 0.138 | 1 |
| CRY2 | 4.72E-06 | -0.635112 | 0.036 | 0.119 | 0.080133 |
| IQCA1 | 0.000287 | -0.635442 | 0.057 | 0.132 | 1 |
| BCAS3 | 4.54E-05 | -0.63558 | 0.035 | 0.107 | 0.771941 |
| C10orf107 | 1.48E-16 | -0.63622 | 0.014 | 0.145 | 2.52E-12 |
| SCGB2A1 | 0.023355 | -0.636333 | 0.327 | 0.377 | 1 |
| PLAC8 | 1.39E-15 | -0.637124 | 0.014 | 0.138 | 2.35E-11 |
| RAD9A | 0.023981 | -0.637437 | 0.07 | 0.119 | 1 |
| UCP2 | 0.022797 | -0.638246 | 0.325 | 0.371 | 1 |
| SPATA6 | 3.94E-07 | -0.638888 | 0.027 | 0.113 | 0.006687 |
| CCDC173 | 2.89E-09 | -0.639048 | 0.015 | 0.101 | 4.91E-05 |
| RIBC1 | 1.84E-19 | -0.640693 | 0.012 | 0.157 | 3.12E-15 |
| RHBDD2 | 0.022584 | -0.641494 | 0.228 | 0.283 | 1 |
| IFT140 | 0.010588 | -0.64655 | 0.057 | 0.107 | 1 |
| MIPEP | 0.001582 | -0.647904 | 0.047 | 0.107 | 1 |
| CYP27A1 | 3.60E-08 | -0.649227 | 0.023 | 0.113 | 0.000611 |
| IFT81 | 2.49E-05 | -0.649554 | 0.04 | 0.119 | 0.422711 |
| ANKS1A | 0.010583 | -0.649557 | 0.07 | 0.126 | 1 |
| STMND1 | 4.67E-17 | -0.650707 | 0.004 | 0.107 | 7.93E-13 |
| MBNL2 | 0.803427 | -0.651276 | 0.107 | 0.107 | 1 |
| NUCB2 | 0.049122 | -0.651614 | 0.062 | 0.101 | 1 |
| DYNLT1 | 0.075597 | -0.652415 | 0.246 | 0.283 | 1 |
| ETS2 | 0.520933 | -0.652606 | 0.188 | 0.157 | 1 |
| CLDN3 | 0.00147 | -0.652614 | 0.636 | 0.591 | 1 |
| AKAP14 | 7.66E-15 | -0.652688 | 0.005 | 0.101 | 1.30E-10 |
| WDR90 | 3.05E-05 | -0.653466 | 0.084 | 0.189 | 0.517649 |
| LOC100507577 | 0.140776 | -0.654082 | 0.12 | 0.075 | 1 |
| HSPB11 | 0.01618 | -0.657302 | 0.136 | 0.195 | 1 |
| PRKAR1A | 0.045118 | -0.657778 | 0.348 | 0.365 | 1 |
| SPAG1 | 3.05E-05 | -0.65858 | 0.064 | 0.157 | 0.518225 |
| NME7 | 0.001627 | -0.659124 | 0.064 | 0.132 | 1 |
| SFI1 | 0.000357 | -0.659218 | 0.048 | 0.119 | 1 |
| BAIAP2L1 | 0.097129 | -0.659417 | 0.167 | 0.214 | 1 |
| ICA1L | 5.44E-09 | -0.659469 | 0.06 | 0.195 | 9.25E-05 |
| ZNF273 | 4.68E-08 | -0.663178 | 0.032 | 0.132 | 0.000795 |
| FAM179B | 0.038078 | -0.663281 | 0.064 | 0.107 | 1 |
| CIPC | 0.091389 | -0.663361 | 0.111 | 0.151 | 1 |
| DNAH11 | 2.36E-12 | -0.663555 | 0.009 | 0.101 | 4.02E-08 |
| CFAP53 | 1.09E-16 | -0.664859 | 0.01 | 0.132 | 1.85E-12 |
| SMIM6 | 1.05E-09 | -0.665208 | 0.028 | 0.138 | 1.78E-05 |
| HSBP1 | 0.034124 | -0.666628 | 0.337 | 0.358 | 1 |
| TSPAN6 | 0.008334 | -0.66864 | 0.172 | 0.245 | 1 |
| LRPAP1 | 0.729708 | -0.673241 | 0.214 | 0.182 | 1 |
| FAM227A | 1.41E-07 | -0.673659 | 0.037 | 0.138 | 0.002397 |
| SYNGAP1 | 1.46E-07 | -0.6747 | 0.035 | 0.132 | 0.002484 |
| SCAMP1 | 0.381039 | -0.677362 | 0.128 | 0.145 | 1 |
| DNAAF3 | 5.13E-14 | -0.678321 | 0.015 | 0.132 | 8.71E-10 |
| TMX4 | 0.001565 | -0.678714 | 0.094 | 0.17 | 1 |
| DNAH9 | 4.02E-17 | -0.680009 | 0.005 | 0.113 | 6.83E-13 |
| ISYNA1 | 0.072992 | -0.68377 | 0.151 | 0.189 | 1 |
| KIAA0895 | 3.56E-05 | -0.684072 | 0.037 | 0.113 | 0.604723 |
| TMEM254 | 0.036787 | -0.685943 | 0.069 | 0.113 | 1 |
| CTXN1 | 0.000469 | -0.686106 | 0.106 | 0.195 | 1 |
| SEPW1 | 0.000939 | -0.688944 | 0.378 | 0.44 | 1 |
| CYB561 | 0.001247 | -0.689033 | 0.225 | 0.314 | 1 |
| PCSK1N | 2.90E-05 | -0.689039 | 0.044 | 0.126 | 0.493083 |
| RGS22 | 6.76E-16 | -0.689654 | 0.004 | 0.101 | 1.15E-11 |
| KLF2 | 0.000158 | -0.689905 | 0.126 | 0.226 | 1 |
| PPP1R32 | 1.26E-26 | -0.690036 | 0.01 | 0.189 | 2.14E-22 |
| KCNK1 | 0.115155 | -0.690577 | 0.112 | 0.151 | 1 |
| CD46 | 0.004492 | -0.692109 | 0.411 | 0.465 | 1 |
| KIAA1211L | 4.00E-05 | -0.694182 | 0.053 | 0.138 | 0.679364 |
| CBY1 | 0.240678 | -0.694826 | 0.095 | 0.119 | 1 |
| EPPK1 | 0.52203 | -0.695946 | 0.131 | 0.138 | 1 |
| PTPRF | 0.524021 | -0.69679 | 0.499 | 0.434 | 1 |
| ANXA1 | 2.64E-05 | -0.697629 | 0.611 | 0.654 | 0.448825 |
| CFAP69 | 7.73E-10 | -0.700811 | 0.058 | 0.201 | 1.31E-05 |
| IFT43 | 7.08E-05 | -0.700839 | 0.165 | 0.277 | 1 |
| PPP1R16A | 0.070371 | -0.701665 | 0.33 | 0.358 | 1 |
| MYB | 8.67E-09 | -0.703782 | 0.032 | 0.138 | 0.000147 |
| ACAT1 | 0.021714 | -0.70397 | 0.07 | 0.119 | 1 |
| CFAP20 | 0.025419 | -0.705428 | 0.164 | 0.22 | 1 |
| IGF1R | 0.087096 | -0.705699 | 0.146 | 0.182 | 1 |
| PALMD | 0.000566 | -0.705923 | 0.047 | 0.113 | 1 |
| FXYD3 | 1.31E-06 | -0.706377 | 0.447 | 0.572 | 0.022248 |
| OSCP1 | 3.61E-09 | -0.706895 | 0.049 | 0.176 | 6.13E-05 |
| PCYT2 | 5.38E-06 | -0.707835 | 0.093 | 0.208 | 0.091398 |
| AK7 | 2.56E-17 | -0.71017 | 0.007 | 0.126 | 4.34E-13 |
| ZNF688 | 3.08E-05 | -0.711526 | 0.041 | 0.119 | 0.522483 |
| KLHL6 | 5.04E-16 | -0.712106 | 0.005 | 0.107 | 8.55E-12 |
| CCDC66 | 0.001649 | -0.712944 | 0.042 | 0.101 | 1 |
| TCTN1 | 3.51E-05 | -0.713392 | 0.081 | 0.182 | 0.595756 |
| CATSPERD | 5.14E-17 | -0.713789 | 0.004 | 0.107 | 8.74E-13 |
| MUC4 | 0.001213 | -0.714391 | 0.085 | 0.164 | 1 |
| MAATS1 | 4.15E-05 | -0.714474 | 0.053 | 0.138 | 0.705032 |
| PKIG | 0.001661 | -0.714633 | 0.094 | 0.17 | 1 |
| CALM2 | 1.52E-08 | -0.714851 | 0.635 | 0.704 | 0.000258 |
| HNRNPF | 0.003612 | -0.714914 | 0.368 | 0.428 | 1 |
| SLAIN2 | 7.36E-05 | -0.715349 | 0.081 | 0.176 | 1 |
| CADM1 | 0.021614 | -0.716039 | 0.185 | 0.245 | 1 |
| DNAAF5 | 0.135194 | -0.71621 | 0.109 | 0.145 | 1 |
| PPP4R3B | 0.026626 | -0.717775 | 0.117 | 0.17 | 1 |
| SYTL3 | 7.20E-09 | -0.719716 | 0.023 | 0.119 | 0.000122 |
| WDR38 | 2.84E-27 | -0.720508 | 0.006 | 0.176 | 4.82E-23 |
| ENDOG | 1.17E-09 | -0.721945 | 0.063 | 0.208 | 2.00E-05 |
| RPN1 | 0.455027 | -0.723218 | 0.31 | 0.296 | 1 |
| PGR | 2.01E-07 | -0.723562 | 0.026 | 0.113 | 0.00342 |
| SMIM19 | 0.035412 | -0.724043 | 0.235 | 0.277 | 1 |
| RAB36 | 2.08E-07 | -0.725043 | 0.032 | 0.126 | 0.003533 |
| FAM166B | 6.58E-24 | -0.727818 | 0.005 | 0.151 | 1.12E-19 |
| DYX1C1 | 3.55E-12 | -0.728654 | 0.021 | 0.138 | 6.04E-08 |
| SYTL1 | 0.000953 | -0.730747 | 0.106 | 0.189 | 1 |
| C6 | 6.22E-16 | -0.732239 | 0.004 | 0.101 | 1.06E-11 |
| JMJD8 | 0.298583 | -0.732944 | 0.137 | 0.157 | 1 |
| CCDC40 | 1.74E-10 | -0.733088 | 0.028 | 0.145 | 2.95E-06 |
| ANXA2P3 | 0.004091 | -0.734514 | 0.437 | 0.472 | 1 |
| C9orf72 | 5.92E-07 | -0.734909 | 0.07 | 0.189 | 0.010051 |
| IFT27 | 1.01E-06 | -0.736486 | 0.06 | 0.17 | 0.017113 |
| CWH43 | 2.52E-13 | -0.736778 | 0.009 | 0.107 | 4.28E-09 |
| ARL13B | 0.01925 | -0.737022 | 0.074 | 0.126 | 1 |
| VWA3B | 1.04E-10 | -0.739859 | 0.014 | 0.107 | 1.76E-06 |
| IFT57 | 1.38E-06 | -0.739989 | 0.162 | 0.302 | 0.023405 |
| HEATR5A | 0.063171 | -0.740562 | 0.063 | 0.101 | 1 |
| NDFIP2 | 0.164638 | -0.741165 | 0.153 | 0.189 | 1 |
| ZNF440 | 2.19E-05 | -0.742109 | 0.063 | 0.157 | 0.371939 |
| SERPINA6 | 2.75E-19 | -0.7425 | 0.004 | 0.119 | 4.68E-15 |
| MORN1 | 0.000193 | -0.744053 | 0.035 | 0.101 | 1 |
| TACC2 | 0.001694 | -0.747711 | 0.084 | 0.157 | 1 |
| SCO2 | 0.001299 | -0.750443 | 0.072 | 0.145 | 1 |
| NME5 | 3.24E-13 | -0.751711 | 0.021 | 0.145 | 5.50E-09 |
| SMPD2 | 2.12E-07 | -0.753222 | 0.046 | 0.151 | 0.003607 |
| LOC100506990 | 2.99E-16 | -0.753637 | 0.016 | 0.151 | 5.08E-12 |
| AKAP9 | 0.002137 | -0.755294 | 0.138 | 0.22 | 1 |
| NDE1 | 5.56E-07 | -0.757961 | 0.025 | 0.107 | 0.009448 |
| DFNB31 | 2.31E-06 | -0.758221 | 0.031 | 0.113 | 0.039181 |
| SAPCD1.AS1 | 1.57E-16 | -0.758403 | 0.014 | 0.145 | 2.66E-12 |
| MORF4L1 | 0.011307 | -0.758584 | 0.301 | 0.352 | 1 |
| GAS8 | 0.000472 | -0.758766 | 0.062 | 0.138 | 1 |
| EFHC2 | 1.15E-09 | -0.760093 | 0.021 | 0.119 | 1.95E-05 |
| CCDC24 | 0.002043 | -0.760868 | 0.109 | 0.189 | 1 |
| HRASLS2 | 2.89E-09 | -0.762695 | 0.025 | 0.126 | 4.90E-05 |
| TDRP | 0.000788 | -0.762847 | 0.099 | 0.182 | 1 |
| CRIP1 | 7.60E-09 | -0.76412 | 0.562 | 0.692 | 0.000129 |
| CCDC151 | 1.68E-10 | -0.764351 | 0.023 | 0.132 | 2.85E-06 |
| TUSC3 | 0.000337 | -0.765489 | 0.169 | 0.27 | 1 |
| C15orf65 | 1.69E-08 | -0.765549 | 0.017 | 0.101 | 0.000287 |
| RPGR | 8.28E-08 | -0.765728 | 0.094 | 0.233 | 0.001406 |
| TCTN2 | 1.01E-06 | -0.76603 | 0.077 | 0.195 | 0.017117 |
| DAW1 | 1.78E-20 | -0.766373 | 0.004 | 0.126 | 3.02E-16 |
| ANKRD42 | 0.000333 | -0.768449 | 0.067 | 0.145 | 1 |
| FHAD1 | 8.97E-17 | -0.769847 | 0.01 | 0.132 | 1.52E-12 |
| LBH | 0.038296 | -0.771619 | 0.146 | 0.201 | 1 |
| LRRC71 | 6.76E-16 | -0.772002 | 0.004 | 0.101 | 1.15E-11 |
| SRI | 0.011524 | -0.772068 | 0.357 | 0.39 | 1 |
| PFN2 | 0.004054 | -0.772393 | 0.22 | 0.296 | 1 |
| ZNF664 | 0.000538 | -0.773127 | 0.273 | 0.365 | 1 |
| STOML3 | 1.33E-21 | -0.774606 | 0.004 | 0.132 | 2.25E-17 |
| CCDC153 | 6.28E-19 | -0.774705 | 0.019 | 0.176 | 1.07E-14 |
| CLSTN1 | 0.017983 | -0.777116 | 0.273 | 0.327 | 1 |
| ATP6V1D | 0.001774 | -0.777896 | 0.091 | 0.164 | 1 |
| HAGHL | 0.014102 | -0.77937 | 0.083 | 0.138 | 1 |
| TCF7 | 1.97E-13 | -0.77949 | 0.032 | 0.176 | 3.35E-09 |
| PTGES3 | 0.018581 | -0.779716 | 0.432 | 0.44 | 1 |
| ADPRHL2 | 0.51077 | -0.779944 | 0.111 | 0.119 | 1 |
| SPATA33 | 6.79E-14 | -0.781268 | 0.023 | 0.157 | 1.15E-09 |
| GOLGA2P5 | 7.70E-05 | -0.781522 | 0.056 | 0.138 | 1 |
| FBXW9 | 3.05E-07 | -0.783743 | 0.069 | 0.189 | 0.005179 |
| EVI5 | 0.002074 | -0.784323 | 0.052 | 0.113 | 1 |
| STEAP3 | 0.000233 | -0.786286 | 0.056 | 0.132 | 1 |
| DALRD3 | 3.61E-05 | -0.787166 | 0.147 | 0.264 | 0.613936 |
| CD59 | 0.000284 | -0.788803 | 0.338 | 0.421 | 1 |
| WDR34 | 0.001556 | -0.789776 | 0.221 | 0.308 | 1 |
| GPR162 | 4.21E-31 | -0.790408 | 0.011 | 0.22 | 7.15E-27 |
| SORBS2 | 6.34E-06 | -0.792435 | 0.105 | 0.226 | 0.10777 |
| KATNB1 | 9.33E-07 | -0.793206 | 0.048 | 0.151 | 0.015842 |
| SUN1 | 1.10E-05 | -0.794277 | 0.243 | 0.377 | 0.186224 |
| TEKT4 | 4.52E-17 | -0.794391 | 0.011 | 0.138 | 7.68E-13 |
| SOCS3 | 0.022695 | -0.795216 | 0.438 | 0.459 | 1 |
| CCDC65 | 2.02E-17 | -0.79651 | 0.007 | 0.126 | 3.42E-13 |
| SPAG17 | 1.66E-12 | -0.798393 | 0.016 | 0.126 | 2.82E-08 |
| CMIP | 1.21E-06 | -0.798531 | 0.096 | 0.22 | 0.020519 |
| KIF3A | 1.75E-06 | -0.799618 | 0.041 | 0.132 | 0.029743 |
| SPEF1 | 2.29E-20 | -0.799879 | 0.03 | 0.22 | 3.89E-16 |
| LRRC6 | 2.14E-09 | -0.800017 | 0.027 | 0.132 | 3.63E-05 |
| C6orf165 | 5.36E-18 | -0.800268 | 0.015 | 0.157 | 9.10E-14 |
| KRT80 | 0.000309 | -0.801104 | 0.065 | 0.145 | 1 |
| MAP1A | 8.26E-10 | -0.801877 | 0.014 | 0.101 | 1.40E-05 |
| CFAP44 | 0.000267 | -0.804921 | 0.047 | 0.119 | 1 |
| AGBL2 | 3.13E-10 | -0.805273 | 0.015 | 0.107 | 5.32E-06 |
| TNFAIP8L1 | 1.31E-17 | -0.805504 | 0.023 | 0.182 | 2.23E-13 |
| UNC119B | 1.60E-08 | -0.808194 | 0.049 | 0.17 | 0.000271 |
| KCNN3 | 3.92E-19 | -0.808222 | 0.022 | 0.189 | 6.66E-15 |
| CYB5R1 | 1.75E-09 | -0.81054 | 0.126 | 0.302 | 2.97E-05 |
| LRTOMT | 5.90E-15 | -0.811605 | 0.028 | 0.176 | 1.00E-10 |
| CFAP57 | 1.58E-18 | -0.812782 | 0.007 | 0.132 | 2.68E-14 |
| SYNE1 | 1.49E-10 | -0.813786 | 0.062 | 0.214 | 2.52E-06 |
| MAP9 | 1.38E-06 | -0.814331 | 0.062 | 0.17 | 0.023367 |
| MGST3 | 1.02E-06 | -0.817918 | 0.278 | 0.421 | 0.017375 |
| APH1B | 5.46E-06 | -0.817989 | 0.04 | 0.126 | 0.092725 |
| CLMN | 2.07E-05 | -0.818884 | 0.079 | 0.182 | 0.351462 |
| MAP1B | 0.001026 | -0.818885 | 0.053 | 0.119 | 1 |
| DNAJA4 | 4.79E-05 | -0.821404 | 0.159 | 0.277 | 0.8142 |
| WDR19 | 0.003366 | -0.821733 | 0.088 | 0.157 | 1 |
| CCDC108 | 9.17E-20 | -0.824335 | 0.009 | 0.145 | 1.56E-15 |
| TSNAXIP1 | 1.15E-09 | -0.824697 | 0.019 | 0.113 | 1.95E-05 |
| DTX3 | 6.89E-05 | -0.826443 | 0.084 | 0.182 | 1 |
| CASZ1 | 0.182645 | -0.827347 | 0.098 | 0.126 | 1 |
| NQO1 | 0.000172 | -0.83176 | 0.116 | 0.214 | 1 |
| TMEM173 | 1.90E-06 | -0.832504 | 0.209 | 0.352 | 0.032323 |
| FAM65B | 9.41E-10 | -0.833118 | 0.023 | 0.126 | 1.60E-05 |
| RGS14 | 1.44E-06 | -0.834331 | 0.047 | 0.145 | 0.024509 |
| C22orf15 | 3.87E-18 | -0.838717 | 0.004 | 0.113 | 6.57E-14 |
| TAX1BP1 | 0.000356 | -0.840403 | 0.196 | 0.302 | 1 |
| IFT88 | 2.51E-05 | -0.841221 | 0.047 | 0.132 | 0.426977 |
| NPHP1 | 8.93E-06 | -0.841959 | 0.041 | 0.126 | 0.151741 |
| ODF2 | 4.35E-07 | -0.843887 | 0.069 | 0.189 | 0.007393 |
| DNAI2 | 2.07E-28 | -0.845474 | 0.005 | 0.176 | 3.51E-24 |
| CTSD | 2.45E-08 | -0.846256 | 0.578 | 0.667 | 0.000415 |
| IFT172 | 5.22E-09 | -0.846595 | 0.085 | 0.233 | 8.87E-05 |
| C17orf97 | 2.86E-11 | -0.847336 | 0.026 | 0.145 | 4.85E-07 |
| KIAA0556 | 1.72E-06 | -0.848957 | 0.051 | 0.151 | 0.029138 |
| STOM | 2.11E-06 | -0.849523 | 0.075 | 0.189 | 0.035831 |
| KIAA1407 | 2.36E-07 | -0.849996 | 0.044 | 0.151 | 0.00401 |
| ATP2A2 | 4.46E-06 | -0.855434 | 0.28 | 0.415 | 0.075826 |
| DPCD | 1.63E-10 | -0.85621 | 0.117 | 0.296 | 2.78E-06 |
| DNAL1 | 0.000173 | -0.858517 | 0.069 | 0.151 | 1 |
| KRT5 | 0.000547 | -0.85981 | 0.054 | 0.126 | 1 |
| SARAF | 0.000102 | -0.861033 | 0.363 | 0.453 | 1 |
| CASC1 | 2.00E-13 | -0.862223 | 0.025 | 0.157 | 3.40E-09 |
| SLC12A7 | 0.041623 | -0.864068 | 0.149 | 0.201 | 1 |
| CCDC81 | 1.01E-22 | -0.864495 | 0.005 | 0.145 | 1.71E-18 |
| CCNDBP1 | 0.004853 | -0.864536 | 0.132 | 0.208 | 1 |
| CHST6 | 6.70E-08 | -0.866874 | 0.046 | 0.157 | 0.001137 |
| GAS2L2 | 1.31E-29 | -0.867311 | 0.005 | 0.182 | 2.22E-25 |
| ARHGAP18 | 8.40E-07 | -0.868257 | 0.042 | 0.138 | 0.014266 |
| TM9SF2 | 0.000603 | -0.870397 | 0.274 | 0.358 | 1 |
| ANKMY1 | 6.85E-10 | -0.8706 | 0.046 | 0.176 | 1.16E-05 |
| DUSP18 | 2.86E-09 | -0.871688 | 0.033 | 0.145 | 4.85E-05 |
| MAPK15 | 6.36E-11 | -0.876153 | 0.135 | 0.327 | 1.08E-06 |
| C9orf9 | 8.55E-13 | -0.877518 | 0.02 | 0.138 | 1.45E-08 |
| HMGN3 | 1.08E-08 | -0.877867 | 0.362 | 0.522 | 0.000184 |
| SHROOM3 | 2.67E-05 | -0.878863 | 0.163 | 0.283 | 0.452696 |
| IFT22 | 1.83E-06 | -0.87985 | 0.132 | 0.264 | 0.03111 |
| DZIP3 | 1.69E-10 | -0.881533 | 0.043 | 0.176 | 2.87E-06 |
| PIH1D2 | 1.67E-15 | -0.88197 | 0.009 | 0.119 | 2.84E-11 |
| ENKUR | 1.06E-21 | -0.883795 | 0.006 | 0.145 | 1.79E-17 |
| PPAP2C | 0.041806 | -0.884644 | 0.211 | 0.258 | 1 |
| DNAH5 | 2.85E-13 | -0.890789 | 0.012 | 0.119 | 4.84E-09 |
| C11orf74 | 9.61E-13 | -0.891607 | 0.04 | 0.189 | 1.63E-08 |
| DYNC2H1 | 2.34E-13 | -0.891916 | 0.027 | 0.164 | 3.98E-09 |
| TRAF3IP1 | 8.71E-10 | -0.892711 | 0.053 | 0.189 | 1.48E-05 |
| ERGIC3 | 0.000121 | -0.897699 | 0.389 | 0.459 | 1 |
| IQCK | 0.000912 | -0.900806 | 0.085 | 0.164 | 1 |
| HYDIN | 6.20E-19 | -0.902051 | 0.015 | 0.164 | 1.05E-14 |
| TMEM110 | 2.66E-12 | -0.902509 | 0.026 | 0.151 | 4.52E-08 |
| ENKD1 | 2.67E-13 | -0.908965 | 0.069 | 0.252 | 4.53E-09 |
| CARS | 1.63E-05 | -0.910714 | 0.084 | 0.189 | 0.276959 |
| LRRIQ1 | 1.33E-10 | -0.912283 | 0.016 | 0.113 | 2.25E-06 |
| FAIM | 3.29E-10 | -0.915649 | 0.084 | 0.245 | 5.59E-06 |
| CCDC69 | 3.07E-22 | -0.917658 | 0.015 | 0.182 | 5.21E-18 |
| GSN | 0.077249 | -0.919387 | 0.419 | 0.415 | 1 |
| HES6 | 2.93E-05 | -0.923724 | 0.12 | 0.239 | 0.498456 |
| ARMC4 | 8.54E-31 | -0.924129 | 0.005 | 0.189 | 1.45E-26 |
| FAM229B | 7.26E-17 | -0.924835 | 0.046 | 0.233 | 1.23E-12 |
| STK33 | 4.31E-11 | -0.925048 | 0.032 | 0.157 | 7.32E-07 |
| MOK | 5.28E-08 | -0.926179 | 0.079 | 0.214 | 0.000897 |
| MGLL | 0.040263 | -0.927667 | 0.2 | 0.252 | 1 |
| MDH1B | 5.41E-13 | -0.927983 | 0.031 | 0.17 | 9.19E-09 |
| PACRG | 6.69E-19 | -0.931102 | 0.01 | 0.145 | 1.14E-14 |
| IL5RA | 2.39E-19 | -0.931271 | 0.005 | 0.126 | 4.05E-15 |
| DNAJB13 | 5.09E-11 | -0.932711 | 0.099 | 0.277 | 8.64E-07 |
| TTC21A | 1.85E-12 | -0.935229 | 0.022 | 0.145 | 3.14E-08 |
| OCEL1 | 1.09E-07 | -0.93713 | 0.062 | 0.182 | 0.001853 |
| DUOX1 | 3.64E-15 | -0.938979 | 0.019 | 0.151 | 6.18E-11 |
| GLT8D1 | 0.006727 | -0.939487 | 0.105 | 0.17 | 1 |
| P4HTM | 0.000462 | -0.943016 | 0.207 | 0.302 | 1 |
| VDAC3 | 0.09797 | -0.94376 | 0.211 | 0.239 | 1 |
| CCDC176 | 1.09E-16 | -0.944886 | 0.026 | 0.182 | 1.86E-12 |
| GLB1L | 9.06E-14 | -0.948119 | 0.017 | 0.138 | 1.54E-09 |
| WDR78 | 4.35E-11 | -0.948723 | 0.03 | 0.151 | 7.39E-07 |
| ARHGAP39 | 8.83E-09 | -0.950639 | 0.078 | 0.22 | 0.00015 |
| ANAPC4 | 4.25E-06 | -0.95436 | 0.091 | 0.208 | 0.072147 |
| ATG9B | 1.06E-10 | -0.95472 | 0.036 | 0.164 | 1.81E-06 |
| ALDH3B1 | 0.000928 | -0.958112 | 0.147 | 0.233 | 1 |
| CEL | 1.31E-12 | -0.958235 | 0.014 | 0.119 | 2.22E-08 |
| SIK1 | 1.41E-05 | -0.959726 | 0.336 | 0.459 | 0.240237 |
| ODF2L | 3.82E-07 | -0.963587 | 0.091 | 0.22 | 0.006487 |
| CCDC28A | 9.93E-10 | -0.965995 | 0.057 | 0.195 | 1.69E-05 |
| FAM83H.AS1 | 1.16E-07 | -0.969565 | 0.074 | 0.201 | 0.001969 |
| PPP1R14C | 9.09E-10 | -0.970321 | 0.041 | 0.164 | 1.54E-05 |
| UCHL1 | 0.001757 | -0.970465 | 0.074 | 0.145 | 1 |
| MLF1 | 3.90E-06 | -0.972421 | 0.132 | 0.258 | 0.066268 |
| BLCAP | 1.07E-06 | -0.97288 | 0.116 | 0.245 | 0.018202 |
| C16orf93 | 1.40E-17 | -0.97887 | 0.026 | 0.189 | 2.38E-13 |
| RSPH4A | 1.44E-28 | -0.979436 | 0.004 | 0.17 | 2.44E-24 |
| MAP3K19 | 1.65E-20 | -0.980552 | 0.002 | 0.119 | 2.80E-16 |
| HIPK1 | 1.75E-09 | -0.980989 | 0.088 | 0.245 | 2.97E-05 |
| IQCG | 4.99E-21 | -0.98189 | 0.048 | 0.27 | 8.47E-17 |
| IRF6 | 3.31E-07 | -0.982071 | 0.12 | 0.258 | 0.00562 |
| EFCAB2 | 2.57E-12 | -0.983566 | 0.044 | 0.195 | 4.36E-08 |
| CCNA1 | 0.005284 | -0.983851 | 0.056 | 0.113 | 1 |
| PYCR2 | 0.000109 | -0.984988 | 0.195 | 0.302 | 1 |
| IQCD | 2.16E-24 | -0.988997 | 0.02 | 0.214 | 3.67E-20 |
| ORC4 | 0.013344 | -0.991439 | 0.125 | 0.189 | 1 |
| C11orf70 | 3.76E-18 | -0.994116 | 0.019 | 0.17 | 6.39E-14 |
| C1orf158 | 3.60E-33 | -0.994204 | 0.005 | 0.201 | 6.12E-29 |
| TP53AIP1 | 4.17E-16 | -0.995225 | 0.006 | 0.113 | 7.08E-12 |
| RFX3 | 1.46E-11 | -0.995694 | 0.058 | 0.214 | 2.49E-07 |
| SSBP4 | 2.92E-06 | -0.997374 | 0.265 | 0.403 | 0.049674 |
| ANKRD37 | 2.38E-08 | -0.998691 | 0.121 | 0.277 | 0.000405 |
| CYSTM1 | 1.31E-07 | -1.000666 | 0.294 | 0.44 | 0.002233 |
| CROCC | 5.24E-08 | -1.008437 | 0.075 | 0.208 | 0.00089 |
| C14orf142 | 2.72E-09 | -1.01145 | 0.084 | 0.233 | 4.62E-05 |
| NEK11 | 7.03E-15 | -1.011686 | 0.041 | 0.208 | 1.19E-10 |
| KIF19 | 4.72E-32 | -1.013039 | 0.005 | 0.195 | 8.02E-28 |
| PDCD6IP | 1.32E-05 | -1.016736 | 0.226 | 0.352 | 0.223872 |
| RFX2 | 2.55E-14 | -1.019197 | 0.012 | 0.126 | 4.33E-10 |
| HN1L | 0.000243 | -1.021795 | 0.18 | 0.277 | 1 |
| SPEF2 | 3.48E-18 | -1.024796 | 0.019 | 0.17 | 5.92E-14 |
| C14orf79 | 0.000543 | -1.038105 | 0.068 | 0.145 | 1 |
| TMEM67 | 5.13E-14 | -1.040365 | 0.053 | 0.226 | 8.72E-10 |
| COBL | 3.54E-07 | -1.041954 | 0.054 | 0.164 | 0.006005 |
| UFC1 | 6.98E-09 | -1.046492 | 0.272 | 0.434 | 0.000119 |
| ELK3 | 2.25E-12 | -1.048171 | 0.042 | 0.189 | 3.82E-08 |
| C2orf81 | 1.48E-15 | -1.048949 | 0.031 | 0.189 | 2.51E-11 |
| CC2D2A | 1.11E-08 | -1.048966 | 0.036 | 0.145 | 0.000188 |
| WDR66 | 7.28E-26 | -1.049559 | 0.036 | 0.27 | 1.24E-21 |
| TYMP | 1.75E-08 | -1.051169 | 0.06 | 0.189 | 0.000297 |
| PITPNM1 | 8.30E-10 | -1.05284 | 0.077 | 0.226 | 1.41E-05 |
| DRC1 | 6.97E-29 | -1.053613 | 0.01 | 0.201 | 1.18E-24 |
| GSTA3 | 9.81E-23 | -1.059711 | 0.004 | 0.138 | 1.67E-18 |
| MNS1 | 4.50E-20 | -1.061921 | 0.019 | 0.182 | 7.64E-16 |
| PPIL6 | 9.64E-19 | -1.064573 | 0.035 | 0.22 | 1.64E-14 |
| C9orf117 | 6.70E-27 | -1.068141 | 0.012 | 0.201 | 1.14E-22 |
| CCDC96 | 2.70E-14 | -1.068794 | 0.016 | 0.138 | 4.59E-10 |
| TCTEX1D2 | 5.10E-16 | -1.069146 | 0.093 | 0.314 | 8.65E-12 |
| DPY30 | 2.95E-06 | -1.070245 | 0.284 | 0.409 | 0.050175 |
| CCDC146 | 7.71E-09 | -1.071032 | 0.133 | 0.302 | 0.000131 |
| GRAMD2 | 9.02E-14 | -1.074092 | 0.054 | 0.226 | 1.53E-09 |
| CDKN1A | 3.73E-10 | -1.07817 | 0.278 | 0.478 | 6.34E-06 |
| DZIP1L | 1.80E-20 | -1.078357 | 0.012 | 0.164 | 3.05E-16 |
| ERICH3 | 7.03E-27 | -1.082802 | 0.012 | 0.201 | 1.19E-22 |
| DYNLRB2 | 9.29E-26 | -1.093382 | 0.011 | 0.189 | 1.58E-21 |
| TCTEX1D4 | 4.48E-17 | -1.103295 | 0.011 | 0.138 | 7.62E-13 |
| CDS1 | 8.26E-10 | -1.110278 | 0.138 | 0.314 | 1.40E-05 |
| ATPIF1 | 4.45E-09 | -1.110919 | 0.484 | 0.604 | 7.56E-05 |
| DNAH10 | 9.26E-23 | -1.1121 | 0.006 | 0.151 | 1.57E-18 |
| MORN5 | 5.91E-38 | -1.115543 | 0.011 | 0.258 | 1.00E-33 |
| GDF15 | 3.09E-09 | -1.116444 | 0.23 | 0.415 | 5.25E-05 |
| LRRC48 | 1.06E-19 | -1.118882 | 0.021 | 0.189 | 1.80E-15 |
| RBM38 | 9.04E-09 | -1.118902 | 0.279 | 0.459 | 0.000153 |
| DYNLL1 | 1.54E-19 | -1.121854 | 0.621 | 0.755 | 2.61E-15 |
| TEKT1 | 4.12E-32 | -1.123006 | 0.006 | 0.201 | 6.99E-28 |
| NUDC | 4.90E-10 | -1.123906 | 0.219 | 0.403 | 8.32E-06 |
| CALM1 | 8.45E-15 | -1.12505 | 0.557 | 0.673 | 1.44E-10 |
| KCNRG | 2.34E-25 | -1.125377 | 0.009 | 0.176 | 3.97E-21 |
| PDK4 | 6.39E-07 | -1.128768 | 0.143 | 0.289 | 0.010846 |
| SPAG8 | 1.12E-32 | -1.132383 | 0.014 | 0.239 | 1.90E-28 |
| SAA1 | 1.03E-05 | -1.134067 | 0.065 | 0.164 | 0.174621 |
| CFAP52 | 1.65E-33 | -1.13621 | 0.011 | 0.233 | 2.80E-29 |
| CCDC113 | 7.75E-32 | -1.141012 | 0.026 | 0.277 | 1.32E-27 |
| C2orf40 | 6.70E-08 | -1.141937 | 0.027 | 0.119 | 0.001139 |
| FANK1 | 1.87E-18 | -1.142997 | 0.04 | 0.233 | 3.17E-14 |
| JAG1 | 3.64E-13 | -1.147092 | 0.094 | 0.289 | 6.19E-09 |
| SPA17 | 8.08E-23 | -1.147236 | 0.027 | 0.226 | 1.37E-18 |
| CENPM | 7.92E-16 | -1.148052 | 0.038 | 0.208 | 1.35E-11 |
| DNAH6 | 1.82E-14 | -1.149643 | 0.009 | 0.113 | 3.09E-10 |
| LDLRAD1 | 3.76E-26 | -1.15591 | 0.033 | 0.264 | 6.38E-22 |
| CTSS | 2.08E-07 | -1.160344 | 0.077 | 0.201 | 0.003535 |
| CIB1 | 5.72E-14 | -1.168277 | 0.296 | 0.528 | 9.72E-10 |
| IFT46 | 4.17E-18 | -1.172414 | 0.047 | 0.245 | 7.09E-14 |
| CKB | 4.23E-19 | -1.173665 | 0.436 | 0.679 | 7.18E-15 |
| C11orf16 | 5.75E-24 | -1.179689 | 0.007 | 0.164 | 9.76E-20 |
| MGAT5 | 2.36E-07 | -1.183149 | 0.069 | 0.189 | 0.004003 |
| TRIP13 | 8.52E-08 | -1.190271 | 0.037 | 0.138 | 0.001447 |
| ARMC3 | 3.80E-21 | -1.190522 | 0.022 | 0.201 | 6.45E-17 |
| TSPAN1 | 7.64E-11 | -1.192745 | 0.215 | 0.421 | 1.30E-06 |
| CSPP1 | 1.18E-10 | -1.193908 | 0.063 | 0.214 | 2.00E-06 |
| TP73 | 2.93E-20 | -1.211465 | 0.011 | 0.157 | 4.97E-16 |
| MAP6 | 3.58E-32 | -1.215991 | 0.009 | 0.214 | 6.08E-28 |
| DNAH12 | 1.50E-28 | -1.219848 | 0.005 | 0.176 | 2.54E-24 |
| ADGRE5 | 1.72E-19 | -1.22471 | 0.107 | 0.371 | 2.92E-15 |
| CCDC74A | 6.96E-26 | -1.229299 | 0.022 | 0.233 | 1.18E-21 |
| TXN | 6.54E-13 | -1.241291 | 0.459 | 0.604 | 1.11E-08 |
| C9orf135 | 8.73E-30 | -1.24772 | 0.006 | 0.189 | 1.48E-25 |
| SPATA17 | 5.04E-28 | -1.248755 | 0.017 | 0.226 | 8.56E-24 |
| C21orf59 | 6.17E-17 | -1.252975 | 0.183 | 0.447 | 1.05E-12 |
| FAM174A | 3.21E-14 | -1.253718 | 0.078 | 0.27 | 5.45E-10 |
| FABP6 | 1.36E-36 | -1.269201 | 0.009 | 0.239 | 2.32E-32 |
| EFCAB12 | 5.43E-27 | -1.271101 | 0.012 | 0.201 | 9.23E-23 |
| LRRC23 | 4.98E-41 | -1.279554 | 0.031 | 0.346 | 8.46E-37 |
| CCDC114 | 1.51E-33 | -1.280718 | 0.025 | 0.283 | 2.57E-29 |
| LRRC10B | 1.02E-37 | -1.290475 | 0.02 | 0.289 | 1.74E-33 |
| AK1 | 1.27E-12 | -1.297293 | 0.112 | 0.308 | 2.15E-08 |
| SPATA18 | 1.30E-29 | -1.309097 | 0.037 | 0.296 | 2.21E-25 |
| RUVBL1 | 4.35E-13 | -1.31504 | 0.159 | 0.371 | 7.38E-09 |
| PPOX | 8.41E-18 | -1.316478 | 0.064 | 0.277 | 1.43E-13 |
| MS4A8 | 2.29E-26 | -1.319024 | 0.022 | 0.233 | 3.88E-22 |
| CCDC42B | 6.14E-51 | -1.32305 | 0.02 | 0.365 | 1.04E-46 |
| DYDC2 | 1.83E-34 | -1.340723 | 0.015 | 0.252 | 3.11E-30 |
| CD164L2 | 2.79E-26 | -1.34976 | 0.014 | 0.201 | 4.74E-22 |
| CFAP70 | 2.20E-21 | -1.355439 | 0.049 | 0.277 | 3.73E-17 |
| WDR54 | 5.86E-29 | -1.361884 | 0.056 | 0.34 | 9.96E-25 |
| LRRC36 | 3.78E-16 | -1.365591 | 0.006 | 0.113 | 6.42E-12 |
| WDR86.AS1 | 7.86E-37 | -1.367652 | 0.016 | 0.27 | 1.33E-32 |
| LRP11 | 2.53E-11 | -1.368354 | 0.075 | 0.239 | 4.30E-07 |
| SAXO2 | 1.31E-21 | -1.377266 | 0.004 | 0.132 | 2.23E-17 |
| C9orf171 | 8.17E-43 | -1.378459 | 0.004 | 0.245 | 1.39E-38 |
| KCNH3 | 4.01E-10 | -1.382779 | 0.038 | 0.164 | 6.82E-06 |
| DNALI1 | 1.73E-29 | -1.385026 | 0.107 | 0.44 | 2.94E-25 |
| UBXN10 | 4.02E-21 | -1.391459 | 0.056 | 0.283 | 6.83E-17 |
| RSPH9 | 1.25E-33 | -1.391553 | 0.011 | 0.233 | 2.12E-29 |
| ROPN1L | 1.66E-41 | -1.393593 | 0.006 | 0.252 | 2.82E-37 |
| AKNA | 4.92E-14 | -1.396858 | 0.048 | 0.214 | 8.36E-10 |
| CEP126 | 8.78E-25 | -1.404279 | 0.03 | 0.245 | 1.49E-20 |
| PRDX5 | 5.70E-26 | -1.418622 | 0.622 | 0.774 | 9.68E-22 |
| VWA3A | 1.07E-16 | -1.420016 | 0.091 | 0.321 | 1.82E-12 |
| DNAI1 | 9.00E-43 | -1.424254 | 0.006 | 0.258 | 1.53E-38 |
| C11orf97 | 6.04E-44 | -1.426777 | 0.004 | 0.252 | 1.03E-39 |
| PIFO | 1.75E-41 | -1.435183 | 0.035 | 0.358 | 2.97E-37 |
| STX2 | 7.50E-19 | -1.438334 | 0.023 | 0.189 | 1.27E-14 |
| IK | 4.36E-20 | -1.441316 | 0.16 | 0.44 | 7.40E-16 |
| MORN2 | 7.04E-28 | -1.459697 | 0.084 | 0.39 | 1.20E-23 |
| IGFBP7 | 6.37E-32 | -1.471161 | 0.311 | 0.717 | 1.08E-27 |
| CRLF1 | 5.26E-24 | -1.483471 | 0.027 | 0.233 | 8.93E-20 |
| FAM216B | 1.62E-46 | -1.491853 | 0.002 | 0.258 | 2.76E-42 |
| SLC44A4 | 9.87E-19 | -1.498719 | 0.219 | 0.503 | 1.68E-14 |
| CFAP43 | 1.53E-30 | -1.501818 | 0.019 | 0.245 | 2.60E-26 |
| MDM2 | 4.93E-15 | -1.522188 | 0.116 | 0.34 | 8.38E-11 |
| DLEC1 | 4.81E-30 | -1.531306 | 0.011 | 0.214 | 8.17E-26 |
| EFHC1 | 6.40E-23 | -1.534617 | 0.117 | 0.409 | 1.09E-18 |
| NWD1 | 3.31E-25 | -1.54784 | 0.025 | 0.233 | 5.62E-21 |
| ODF3B | 1.27E-39 | -1.549368 | 0.049 | 0.39 | 2.16E-35 |
| TUBA4B | 2.65E-68 | -1.5522 | 0.007 | 0.396 | 4.50E-64 |
| TUBB4B | 5.03E-34 | -1.572917 | 0.509 | 0.805 | 8.54E-30 |
| C6orf118 | 2.40E-37 | -1.574531 | 0.012 | 0.258 | 4.07E-33 |
| IDS | 9.68E-14 | -1.575116 | 0.175 | 0.403 | 1.64E-09 |
| RRAD | 6.96E-23 | -1.575686 | 0.205 | 0.528 | 1.18E-18 |
| PERP | 4.61E-24 | -1.575959 | 0.412 | 0.679 | 7.83E-20 |
| FAM92B | 1.65E-63 | -1.578209 | 0.005 | 0.358 | 2.80E-59 |
| TUBA1A | 1.70E-19 | -1.583928 | 0.293 | 0.56 | 2.89E-15 |
| CFAP45 | 1.22E-36 | -1.631403 | 0.02 | 0.283 | 2.08E-32 |
| PRR29 | 8.38E-46 | -1.63172 | 0.021 | 0.34 | 1.42E-41 |
| C21orf58 | 6.97E-32 | -1.682983 | 0.072 | 0.396 | 1.18E-27 |
| CETN2 | 2.65E-22 | -1.693066 | 0.186 | 0.478 | 4.50E-18 |
| CCDC170 | 1.04E-46 | -1.694981 | 0.036 | 0.39 | 1.77E-42 |
| TMEM231 | 1.12E-45 | -1.719029 | 0.065 | 0.465 | 1.90E-41 |
| CCDC37 | 5.31E-50 | -1.737453 | 0.009 | 0.308 | 9.03E-46 |
| DNAAF1 | 2.58E-53 | -1.778047 | 0.016 | 0.358 | 4.39E-49 |
| C9orf116 | 1.48E-36 | -1.796953 | 0.089 | 0.453 | 2.52E-32 |
| FAM81B | 8.30E-37 | -1.804154 | 0.023 | 0.296 | 1.41E-32 |
| TMEM190 | 4.36E-71 | -1.836597 | 0.01 | 0.421 | 7.40E-67 |
| C7orf57 | 5.95E-42 | -1.882238 | 0.012 | 0.283 | 1.01E-37 |
| CAPSL | 1.66E-58 | -1.88355 | 0.007 | 0.346 | 2.81E-54 |
| C1orf194 | 1.53E-61 | -1.88835 | 0.019 | 0.409 | 2.59E-57 |
| C5orf49 | 8.04E-55 | -1.888533 | 0.06 | 0.503 | 1.37E-50 |
| ZBBX | 2.18E-46 | -1.917406 | 0.022 | 0.346 | 3.70E-42 |
| FAM179A | 4.30E-54 | -1.948759 | 0.012 | 0.346 | 7.30E-50 |
| BAIAP3 | 1.02E-50 | -1.961749 | 0.032 | 0.403 | 1.73E-46 |
| EFCAB1 | 6.59E-64 | -1.968625 | 0.012 | 0.396 | 1.12E-59 |
| AGR3 | 6.85E-43 | -1.996741 | 0.111 | 0.535 | 1.16E-38 |
| CFAP126 | 3.66E-73 | -2.056475 | 0.027 | 0.497 | 6.21E-69 |
| CAPS | 1.66E-48 | -2.061779 | 0.285 | 0.742 | 2.82E-44 |
| AQP3 | 3.80E-34 | -2.089677 | 0.09 | 0.44 | 6.46E-30 |
| PSENEN | 1.55E-32 | -2.116751 | 0.264 | 0.629 | 2.63E-28 |
| CCDC17 | 1.62E-65 | -2.120267 | 0.021 | 0.44 | 2.75E-61 |
| LRRC46 | 1.69E-67 | -2.128705 | 0.011 | 0.409 | 2.88E-63 |
| CDHR3 | 4.05E-72 | -2.170713 | 0.015 | 0.447 | 6.89E-68 |
| ZMYND10 | 1.02E-95 | -2.1946 | 0.023 | 0.597 | 1.74E-91 |
| SNTN | 3.06E-84 | -2.217026 | 0.011 | 0.491 | 5.20E-80 |
| C9orf24 | 3.85E-81 | -2.247221 | 0.014 | 0.484 | 6.54E-77 |
| FAM183A | 2.03E-80 | -2.28493 | 0.017 | 0.497 | 3.45E-76 |
| CDHR4 | 9.74E-88 | -2.28804 | 0.005 | 0.478 | 1.66E-83 |
| RSPH1 | 1.92E-79 | -2.339335 | 0.043 | 0.579 | 3.25E-75 |
| C11orf88 | 7.36E-78 | -2.34704 | 0.01 | 0.453 | 1.25E-73 |
| FOXJ1 | 2.17E-80 | -2.577625 | 0.286 | 0.906 | 3.69E-76 |
| C20orf85 | 1.31E-129 | -3.02358 | 0.012 | 0.704 | 2.22E-125 |
| TPPP3 | 9.35E-113 | -3.102159 | 0.1 | 0.849 | 1.59E-108 |
